# Precision Stealth Nanofibers via PET‐RAFT Polymerisation: Synthesis, Crystallization‐driven Self‐assembly and Cellular Uptake Studies

**DOI:** 10.1002/chem.202500108

**Published:** 2025-03-12

**Authors:** Steven T. G. Street, Ekaterina Shteinberg, Juan Diego Garcia Hernandez, Hayley C. Parkin, Robert L. Harniman, Stephanie Willerth, Ian Manners

**Affiliations:** ^1^ School of Chemistry University of Bristol Cantock's Close Bristol BS8 1TS United Kingdom; ^2^ Department of Chemistry University of Victoria 3800 Finnerty Rd Victoria, BC V8W 3V6 Canada; ^3^ Centre for Advanced Materials and Related Technology (CAMTEC) University of Victoria 3800 Finnerty Rd Victoria, BC V8P 5C2 Canada; ^4^ School of Chemistry University of Birmingham Edgbaston B15 2TT United Kingdom; ^5^ Department of Mechanical Engineering Division of Medical Sciences University of Victoria 3800 Finnerty Rd Victoria, BC V8W 2Y2 Canada; ^6^ School of Biomedical Engineering University of British Columbia 2222 Health Sciences Mall Vancouver, BC V6T 1Z4 Canada

**Keywords:** Self-assembly, Polymers, Oxygen-tolerant polymerization, Micelles, Nanomedicine

## Abstract

Stealth precision polymer nanofibers show great promise as therapeutic delivery systems. However, existing systems are largely limited to poly(ethylene glycol) (PEG) and suffer from challenging functionalization, hampering their translation. This work develops a modular, easily functionalizable platform for biocompatible stealth nanofibers based on a combination of ring‐opening polymerisation (ROP), photoinduced electron/energy transfer reversible addition–fragmentation chain transfer (PET‐RAFT) polymerisation, and crystallization‐driven self‐assembly (CDSA). Low length‐dispersity poly(fluorenetrimethylenecarbonate)‐*b*‐poly(N‐(2‐hydroxypropyl) methacrylamide) (PFTMC‐*b*‐PHPMA) nanofibers may be produced in a single‐step via CDSA, with a length that is dependent on the PHPMA DP_n_. Separately, living CDSA leads to nanofibers with length control between 30 nm and ca. 700 nm. Incorporation of fluorescein into the PET‐RAFT polymerization results in fluorescent PFTMC‐*b*‐PHPMA block copolymers that can undergo CDSA, forming fluorescent nanoparticles for preliminary cell studies. PFTMC‐*b*‐PHPMA nanofibers exhibited minimal toxicity to cells as well as limited cellular association, in line with previous studies on neutral polymer nanofibers. In comparison, PFTMC‐*b*‐PHPMA nanospheres exhibited no cellular association. These results indicate that the unique shape and core‐crystallinity of PFTMC‐*b*‐PHPMA nanofibers ideally positions them for use as therapeutic delivery systems. Overall, the results described herein provide the basis for a modular, easily functionalizable platform for precision stealth polymer nanofibers for a variety of prospective biomedical applications.

## Graphical abstract

Stealth polymer nanofibers are promising therapeutic delivery systems that suffer from challenging functionalization. In this work we harness photoinduced electron/energy transfer reversible addition–fragmentation chain transfer (PET‐RAFT) polymerisation and crystallization‐driven self‐assembly (CDSA) to produce low length‐dispersity poly(fluorenetrimethylenecarbonate)‐b‐poly(N‐(2‐hydroxypropyl) methacrylamide) (PFTMC‐b‐PHPMA) precision nanofibers. Fluorescent PFTMC‐b‐PHPMA nanofibers exhibited minimal cytotoxicity and limited cellular association, in keeping with stealth materials.

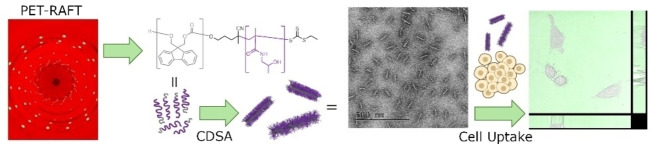

## Introduction

Polymer‐based nanoparticles represent an important component of emerging nanomedicines for disease treatment.^[1]^ Whilst the history of polymeric nanomedicine dates back to the 1950s,^[2]^ to date only a limited number have been commercialized.^[3]^ Amongst polymeric nanomedicines, ‘stealth’ polymers that can avoid immune recognition show great promise as therapeutic delivery systems with long circulation times. However, they often require targeting groups for active transport into the target cell type.[^4^, ^5^] Several classes of stealth polymer have been reported, including most famously poly(ethylene glycol) (PEG) as well as poly(2‐oxazoline) (POx), poly(zwitterions) and poly(N‐(2‐hydroxypropyl) methacrylamide) (PHPMA).[^6^, ^7^] A common feature of these polymers is a neutral overall charge and hydrophilicity which minimises opsonisation compared to hydrophobic or charged polymers.^[8]^

PEG remains the most widely studied stealth polymer; ‘PEGylation’ or the covalent attachment of PEG to biomolecules is a widely employed strategy to improve the pharmaceutical properties of potential therapeutics.^[10]^ Whilst PEG is a useful stealth polymer, it suffers from several drawbacks including anti‐PEG antibodies and accelerated blood clearance as well as a difficult synthesis that makes producing functionalized PEG very challenging.[^6^, ^11^] Thus, alternative stealth polymers with improved properties are highly desirable for biomedical applications.

Neutral polymers such as PEG have been used extensively in the preparation of self‐assembled polymeric nanomedicines. Crystallization‐driven self‐assembly (CDSA) has emerged as a promising method for the preparation of non‐spherical polymeric nanomaterials with tuneable, precisely defined dimensions and low size dispersity.[^12^, ^13^, ^14^] CDSA is a promising technology for producing polymeric nanomedicines as particle shape is known to have an important effect on their biological fate.[^1^, ^4^, ^14^, ^15^, ^16^] Anisotropic nanoparticles such as ‘1D’ nanofibers are promising nanomedicine candidates because they exhibit improved circulation,[^17^, ^18^] extravasation,[^19^, ^20^, ^21^] biodistribution,^[22]^ tissue penetration,[^23^, ^24^] and cellular uptake[^25^, ^26^, ^27^] when compared to spherical systems. Polymer nanofibers produced via CDSA are a promising class of materials for biomedical applications that include therapeutic delivery,[^27^, ^28^, ^29^, ^30^, ^31^, ^32^] antimicrobial activity,[^29^, ^33^, ^34^] bioimaging[^35^, ^36^, ^37^] and tissue engineering[^38^, ^39^] due to their anisotropic shape and core‐crystallinity. Despite this, producing functional neutral polymeric nanofibers remains challenging. For such systems, PEG remains the most widely studied solubilizing coronal block. PEG has been used in the preparation of many CDSA systems including those based upon poly(fluorenetrimethylenecarbonate) (PFTMC),[^9^, ^40^] poly(lactic acid) (PLA),[^41^, ^42^] poly(dihexylfluorene) (PDHF),^[43]^ poly(3‐hexylthiophene),^[44]^ and more.[^36^, ^45^] Other stealth polymers used for CDSA include poly(oligo(ethylene‐ glycol methacrylate)) (POEGMA),[^24^, ^46^] POx derivatives,[^18^, ^25^, ^30^, ^47^] and PHPMA.^[48]^ PHPMA offers several advantages as a stealth polymer over PEG including the ability to functionalize the alcohol side‐chain with therapeutic cargo or imaging agents as well as a versatile synthesis via living radical polymerization techniques such as reversible addition‐fragmentation chain transfer (RAFT) polymerization.[^49^, ^50^, ^51^, ^52^, ^53^] For these reasons, functionalized PHPMA materials have been widely used in biomedicine with particular focus on their use as polymer‐drug conjugates and as hydrogels for tissue engineering.[^49^, ^51^, ^54^]

**Figure 1:**
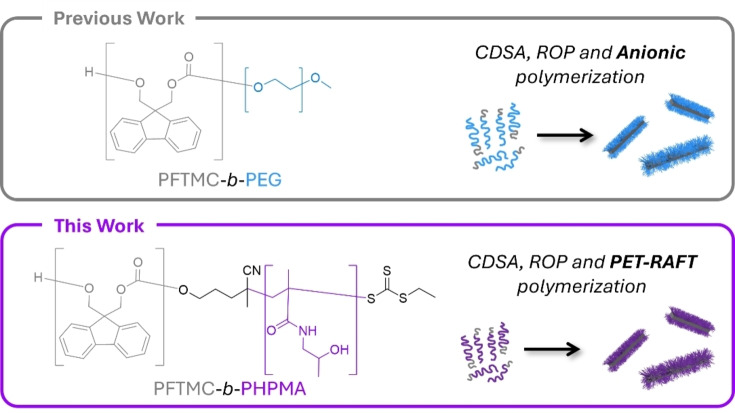
Previously we demonstrated that PFTMC‐*b*‐PEG can self‐assemble into biocompatible nanofibers.^[9]^ In this work we describe the synthesis and self‐assembly of a new block copolymer, PFTMC‐*b*‐PHPMA, into biocompatible nanofibers. Incorporating a fluorescent dye enables us to demonstrate their stealth properties via preliminary cellular studies.

Our previous work demonstrated that PFTMC is an excellent biocompatible and degradable core‐forming block that is capable of robust CDSA with a wide range of coronal chemistries.[^9^, ^37^, ^55^] PFTMC‐based nanofibers show great promise for nanomedicine applications, especially in drug delivery,[^9^, ^32^, ^56^, ^57^] nucleic acid delivery,[^27^, ^28^, ^55^] and as antimicrobials.[^29^, ^33^] PFTMC‐*b*‐PEG nanofibers can be readily prepared via CDSA and are cytocompatible yet exhibit no antimicrobial or anticancer activity.[^9^, ^33^] Furthermore, hydrophobic cargo can be non‐covalently complexed to the core‐corona interface of the nanofibers.[^56^, ^57^] However, the cellular uptake and localization of PFTMC‐*b*‐PEG nanofibers is unknown, largely due to the difficulty of functionalizing such materials with fluorescent dyes or imaging agents. We have also demonstrated that stealth PDHF‐*b*‐PEG nanofibers do not undergo cellular internalization after 1 hour incubation unless a targeting group was employed for active transport into the cell.^[35]^ We envisioned that PFTMC‐*b*‐PHPMA nanofibers would be a useful tool to study the cellular uptake and localization of neutral, stealth nanofibers with a biodegradable core, and that such materials would be highly desirable for a range of applications due to their ease of modification. Here, we address the challenge of producing functional stealth nanofibers via CDSA by synthesising precision PFTMC‐*b*‐PHPMA nanofibers via photoinduced electron/energy transfer reversible addition–fragmentation chain transfer (PET‐RAFT) polymerization (Figure 1). After functionalisation with an imaging agent, preliminary cell studies demonstrated their biocompatibility and stealth characteristics.

## Results and Discussion

### Synthesis and Characterization of PFTMC‐*b*‐PHPMA

**Scheme 1:**
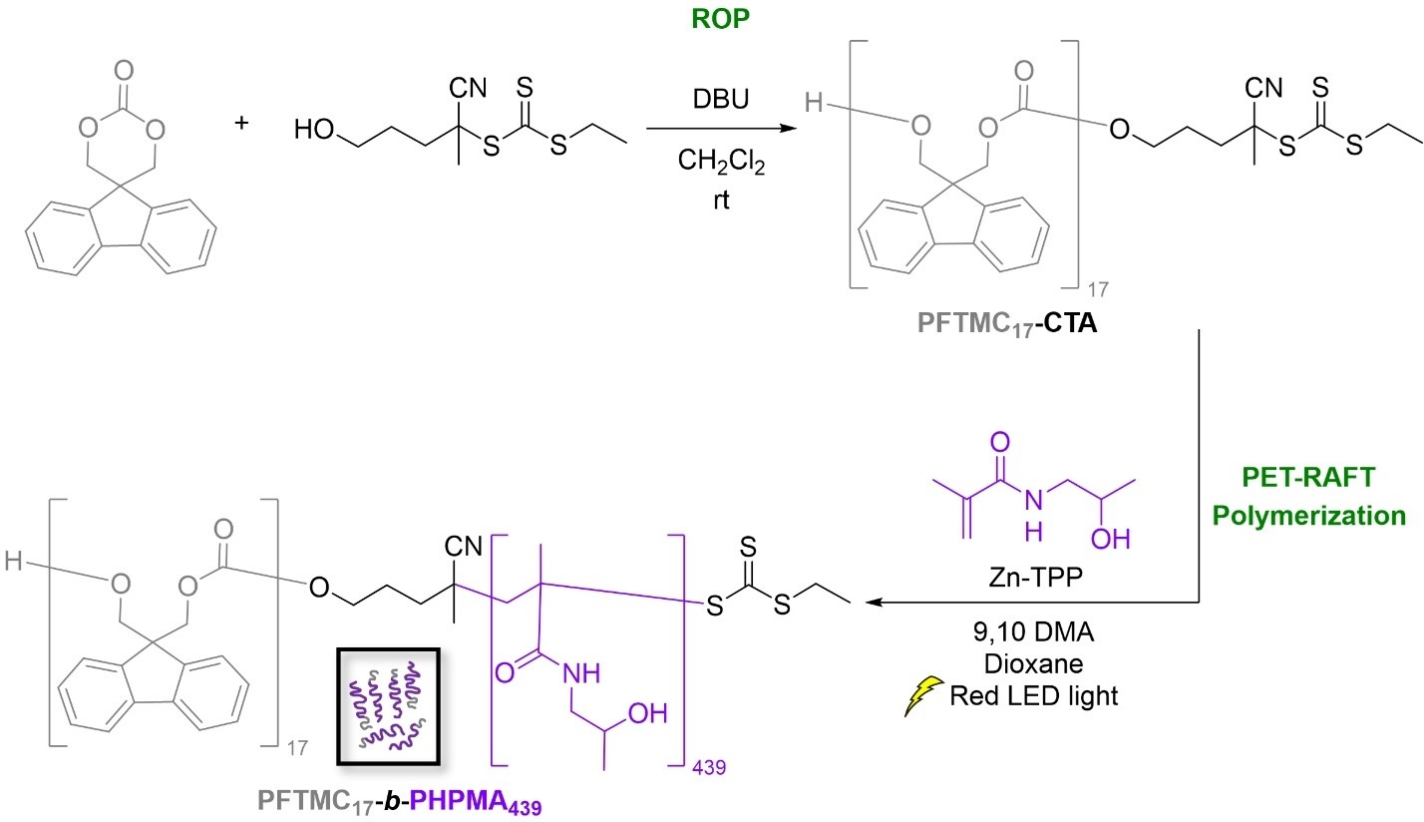
Synthesis of PFTMC_17_‐*b*‐PHPMA_439_ via ROP and PET‐RAFT polymerization.

PFTMC‐*b*‐PHPMA was synthesized via a combination of ring‐opening polymerization (ROP) and PET‐RAFT polymerization in a two‐step procedure (Scheme 1). Firstly, ROP of FTMC with a dual‐headed RAFT/ROP chain transfer agent (CTA) yielded a PFTMC macromolecular chain‐transfer agent (PFTMC_17_‐CTA) according to our previous procedure.^[55]^ The resulting PFTMC_17_‐CTA was characterized via gel‐permeation chromatography (GPC, number‐average molecular weight, *M*_n_=4,400 Da; dispersity, *Đ*_M_=1.21, Figure S1), matrix‐assisted laser desorption/ionization time‐of‐flight mass spectrometry (MALDI‐TOF MS, *M*_n_=4,500 g mol^−1^, DP_n_=17, Figure S2), as well as ^1^H, ^13^C and DOSY NMR spectroscopy (*M*_n_=5,300 Da, DP_n_=20, diffusion coefficient, *D*,=1.11×10^−5^ cm^2^/sec, Figure S3). As sustainability is a key consideration in modern polymer science, we were keen to synthesize the PHPMA block of PFTMC‐*b*‐PHPMA via visible light‐induced PET‐RAFT polymerization. In comparison to conventional RAFT polymerization, PET‐RAFT polymerization is oxygen tolerant, cheaper, more sustainable, and has increased spatiotemporal control.[^58^, ^59^] It is also amenable to high‐throughput experimentation.[^60^, ^61^] To ensure controlled, efficient PET‐RAFT polymerization, we constructed a PET‐RAFT reactor to the specifications of Corrigan et. al. (Figure S4) with minor adaptations to use it as a batch photoreactor as it fitted perfectly over a conventional hotplate (∅ =15 cm).^[62]^ We explored the synthesis of PFTMC‐*b*‐PHPMA from PFTMC_17_‐CTA via PET‐RAFT using red LEDs as a light source, 5,10,15,20‐tetraphenyl‐21H,23H‐porphine zinc (ZnTPP) as a photoredox catalyst, and 9,10‐dimethylanthracene as a singlet oxygen quencher in dioxane.[^63^, ^64^] Under these conditions, the polymerization of PFTMC‐*b*‐PHPMA at 13 wt % solids resulted in the formation of an organogel in a polymerization‐induced self‐assembly (PISA) type process (Figure S5A). This organogel was found to dissolve upon the addition of MeOH, yielding PFTMC_17_‐*b*‐PHPMA_168_ which contained PFTMC homopolymer as an impurity. Removal of the homopolymer was challenging due to the limited solubility of the system (Table S1). MeOH was found to be the best solvent, though dissolution was slow. A second PET‐RAFT reaction under analogous conditions yielded crude PFTMC_16_‐*b*‐PHPMA_160_, which was diluted in MeOH and assessed via Transmission electron microscopy (TEM). TEM analysis of this solution revealed anisotropic nanofiber and nanoplatelet structures (length, *L*_n_,=102±58 nm, length dispersity, *Đ*_L_,=1.32), indicating that the PISA process and slow dissolution in MeOH is likely due to polymer self‐assembly (Figure S5B). The anisotropic shape of the resulting species indicates that this is likely a polymerization‐induced, crystallization‐driven self‐assembly (PI‐CDSA) process.[^40^, ^65^, ^66^, ^67^] Producing ca. 100 nm polymer nanofibers in a one‐pot, single‐step process via oxygen‐tolerant, visible light polymerization‐induced self‐assembly represents a useful method of producing biologically relevant polymeric nanomaterials, even despite their increased length‐dispersity.

Prior work has illustrated that the removal of PFTMC homopolymer from PFTMC block copolymers can be highly challenging.[^9^, ^55^] This was also the case for PFTMC‐*b*‐PHPMA. This issue was addressed via precipitation of the insoluble PFTMC_17_‐*b*‐PHPMA_168_ and PFTMC_16_‐*b*‐PHPMA_160_ from MeOH into THF or a THF/MeCN mixture, which facilitated the removal of the soluble PFTMC homopolymer. The removal of PFTMC homopolymer was confirmed via the disappearance of PFTMC homopolymer signals in the MALDI‐TOF MS spectrum (Figure S6). This yielded PFTMC_17_‐*b*‐PHPMA_439_ (*M*_n_=5,700 Da; *Đ*_M_=1.83 via GPC, *M*_n_=67,400 Da via ^1^H‐NMR, *D* 7.09×10^−6^ cm^2^/sec, Figure S7) and PFTMC_16_‐*b*‐PHPMA_73_ (*M*_n_=1,900 Da; *Đ*_M_=2.81 via GPC, *M*_n_=14,700 Da via ^1^H‐NMR, *D*,=4.59×10^−7^ cm^2^/sec, Figure S8), with changes in the DP_n_ due to fractionation during purification. As the polymerization of HPMA in aprotic solvents is known to be challenging due to hydrogen bonding interactions,^[68]^ the higher dispersities obtained for PFTMC‐*b*‐PHPMA in this work likely results from the use of dioxane, which is a poor solvent for the polymerization of HPMA. Furthermore, dioxane is also known to form complexes with Zinc porphyrins such as ZnTPP.^[69]^ This likely competes with energy transfer to the RAFT CTA, leading to reduced polymerization rates and lower initiation.[^63^, ^70^] Nonetheless, yields of crude PFTMC‐PHPMA were >70 % after 24 h and close to the expected DP_n_, indicating the polymerization proceeded to a high conversion. The addition of MeCN to the selective precipitation of PFTMC_16_‐*b*‐PHPMA_160_ led to increased fractionation and hence the increased dispersity observed for PFTMC_16_‐*b*‐PHPMA_73_. A detailed investigation of the polymerization process will be the subject of future work. Whilst the limited solubility of PFTMC‐*b*‐PHPMA hampered the polymerization, it also provided opportunities for PI‐CDSA and the facile removal of homopolymer, enabling further self‐assembly and biological studies to take place. Characterization data for all the polymers prepared in this work is found in Table S2.

### Crystallization‐Driven Self‐Assembly of PFTMC‐*b*‐PHPMA

**Figure 2:**
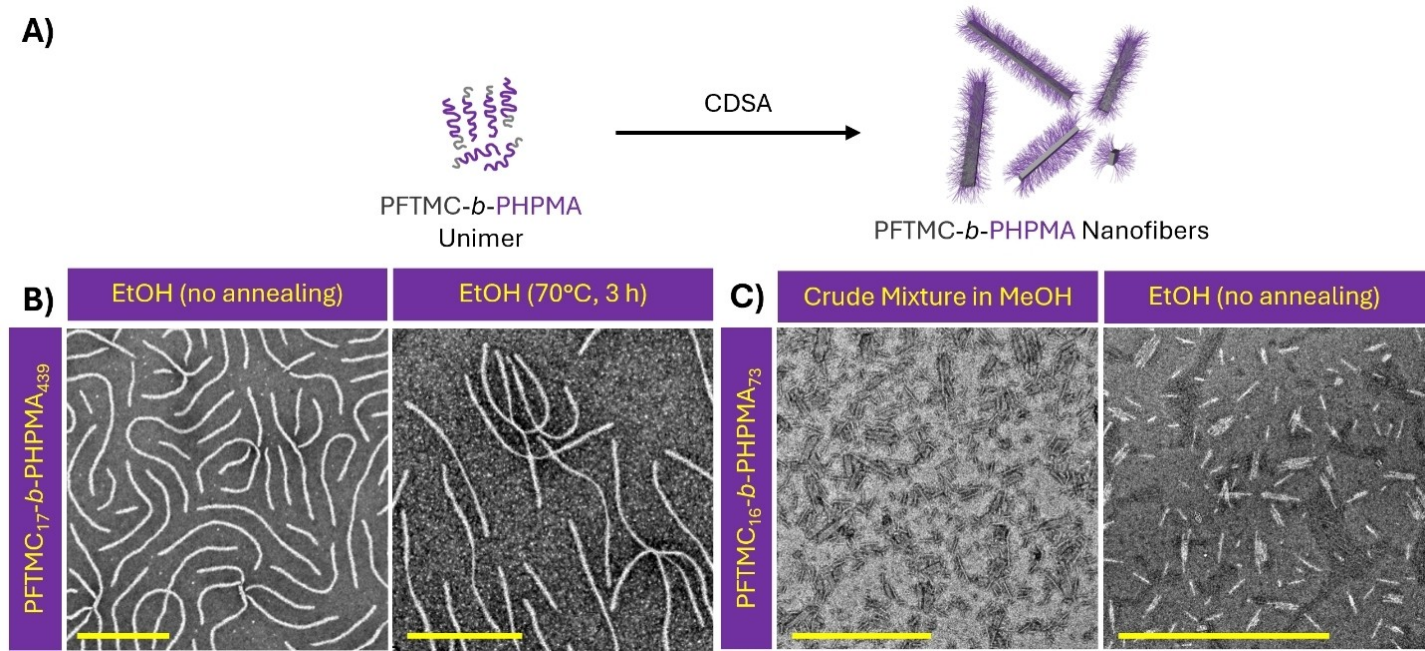
Self‐assembly screening for PFTMC‐*b*‐PHPMA with (A) the general procedure for CDSA; (B–C) TEM micrographs of the assemblies of (B) PFTMC_17_‐*b*‐PHPMA_439_ in DMSO/EtOH (5 : 95 *v*/*v*) after ageing at 23 °C for 24 h or after annealing at 70 °C for 3 h followed by ageing at room temperature; and of (C) PFTMC_16_‐*b*‐PHPMA_73_ in MeOH after dilution from the crude reaction mixture, and (D) low length‐dispersity nanofibers after purification and ageing at 23 °C for 24 h in DMSO/EtOH (5 : 95 *v*/*v*). Scale=1 μm. All samples were stained with uranyl acetate (3 wt % in EtOH).

Previous PFTMC‐based block copolymers have exhibited robust CDSA into morphologically pure nanofibers using either DMSO or THF as a common solvent for both blocks and alcohols as a selective solvent for the coronal block. PFTMC_17_‐*b*‐PHPMA_439_ exhibited limited solubility in many solvents including THF, however DMSO was found to be an acceptable common solvent for self‐assembly. Preliminary studies examined the self‐ assembly of PFTMC_17_‐*b*‐PHPMA_439_ upon addition of a DMSO stock solution (20 mg/mL) to EtOH (5 % *v*/*v* DMSO, 1 mg/mL) and either ageing at room temperature (23 °C) for 24 h or annealing at 70 °C for 3 h and then ageing for 21 h (Figure 2A and B). Under these conditions, morphologically pure nanofibers were formed for both samples. The observed nanofibers were much shorter than those obtained from previous PFTMC systems with a length of 1,149±534 nm (*Đ*_L_=1.22) after aging at 23 °C and 1,541±316 nm (*Đ*_L_=1.04) for the sample annealed at 70 °C for 3 h. The shorter nanofiber lengths obtained for this system indicates that the rate of self‐nucleation is very high, presumably due to poor solubility in the selected solvents. Annealing the sample at 70 °C prior to ageing is sufficient to increase the nanofiber length and lower the *Đ*_L_, indicative of self‐seeding during the annealing process.^[71]^ The high rate of self‐nucleation observed for this system was sufficient to result in low length‐dispersity nanofibers (*Đ*_L_=1.04) directly after annealing, providing a facile route for the production of precision PFTMC‐*b*‐PHPMA nanofibers via a single‐step process. This represents a promising development as to date there have been limited reports of low size‐dispersity nanoparticles via a single‐step CDSA process.^[72]^ The width (number‐average width, *W*_n_) of the nanofibers was 39±10 nm (width dispersity, *Đ*_W,_=1.06) which is also larger and more disperse than previous PFTMC systems. The cause of this increased width appears to be due to the fusion of multiple individual nanofibers into a larger multi‐nanofiber fibril, however less chain‐folding of PFTMC within the core and the inclusion of the PHPMA coronal block in width measurements cannot be discounted. Individual nanofibers also displayed two distinct width populations, in accordance with prior observations that PFTMC nanofibers have a rectangular core cross‐section and may lie on either face on the TEM grid.^[9]^ Intriguingly, PFTMC_17_‐*b*‐PHPMA_439_ nanofibers exhibited a clear tendency to fuse together, with scarf‐like micelles,^[73]^ spherical supermicelles^[74]^ (Figure S9) and adjacently‐fused nanofibers (Figure S10) all present in minor quantities. These structures are presumably formed through hydrogen bonding interactions between the PHPMA corona of adjacent nanofibers in a process analogous to that observed for poly(ferrocenyldimethylsilane)‐*b*‐poly(hydroxyl‐functionalized methylvinylsiloxane) cylindrical micelles.^[75]^

The CDSA of PFTMC_16_‐*b*‐PHPMA_73_ was also examined to probe the effects of HPMA block length on self‐assembly (Figure 2C and Figure S11). Most interestingly, the shorter HPMA block resulted in the formation of low‐dispersity 99±30 nm nanofibers (*Đ*_L_=1.10) in DMSO/EtOH (5 : 95 *v*/*v*, 10 mg/mL) after ageing at 23 °C for 24 h (Figure 2C). These nanofibers were found to fuse together into scarf‐like micelles. After transfer into water via dialysis, these scarf‐like micelles were disrupted, forming predominantly individual nanofibers (*L*_n_=106±29 nm, *Đ*_L_=1.07, Figure S11B). This represents a facile route to low length‐dispersity PFTMC‐*b*‐PHPMA nanofibers of biologically relevant lengths via a single‐step assembly process. The shorter HPMA block presumably leads to shorter nanofibers because of faster self‐nucleation due to its reduced solubility. It is important to note that whilst the rate of self‐nucleation appears very high for PFTMC‐*b*‐PHPMA systems, it is nonetheless sufficient to enable core‐crystallization (and hence CDSA) to take place.

**Figure 3:**
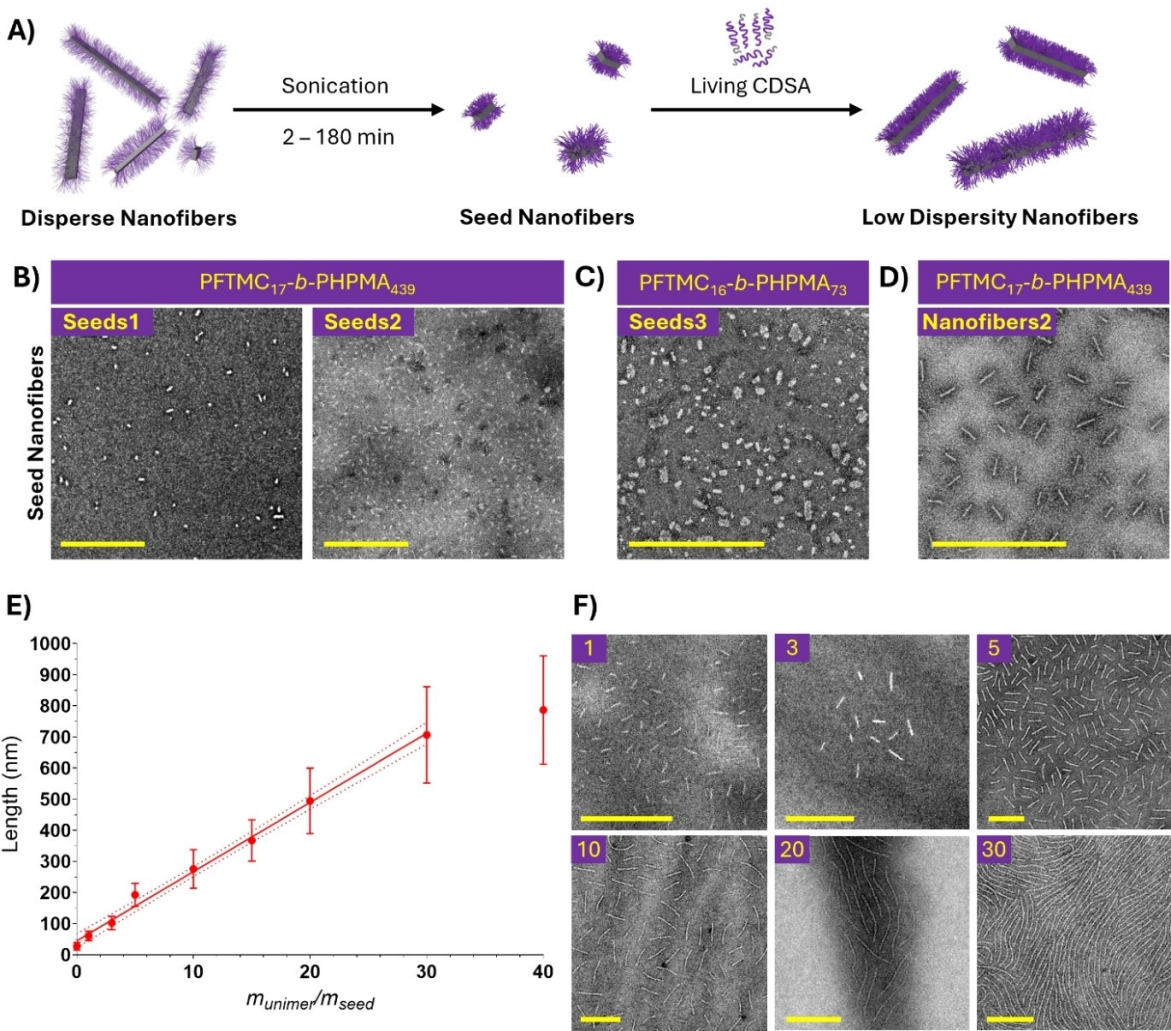
(A) Living CDSA schematic; (B–D) TEM micrographs of (B–C) seed PFTMC‐*b*‐PHPMA nanofibers in DMSO/EtOH (5 : 95 *v*/*v*) and (D) low length‐dispersity nanofibers2 used for cytotoxicity studies after transfer into water; (E) plot of *m*_unimer_/*m*_seed_ vs nanofiber length demonstrating living CDSA and (F) TEM micrographs of length‐controlled PFTMC_17_‐*b*‐PHPMA_439_ nanofibers. Scale=500 nm. All samples were stained with uranyl acetate (3 wt % in EtOH).

**Figure 4:**
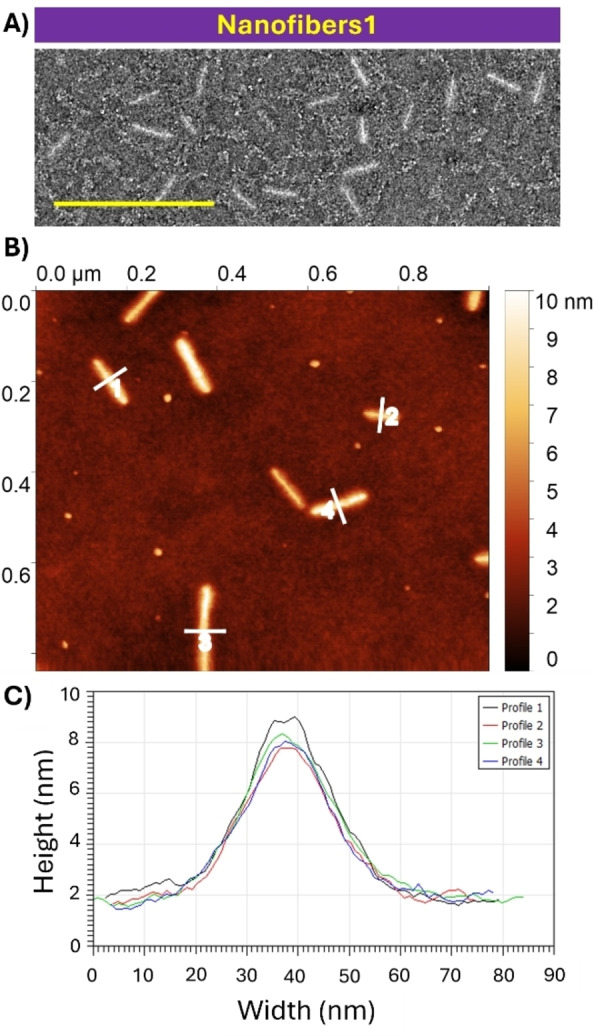
Assessing the height of 103 nm±20 nm PFTMC_17_‐*b*‐PHPMA_439_ nanofibers1 via AFM using a carbon‐coated copper TEM grid as the substrate. (A) TEM micrograph; (B) AFM height profile; (C) linear height profiles of numbered lines in A. The TEM sample was stained with uranyl acetate (3 wt % in EtOH). Scale=500 nm. The AFM sample used a TEM grid as the substrate, but was not stained with uranyl acetate.

Seed PFTMC_17_‐*b*‐PHPMA_439_ nanofibers were formed from sonication of disperse nanofibers in DMSO/EtOH (5 : 95 *v*/*v*, 1 mg/mL). This yielded 27 nm±12 nm (*Đ*_L_=1.19) nanofiber **seeds1** (Figure 3B). A detailed study of the sonication kinetics for this sample is in the supporting information (Figure S12), corroborating prior studies on the kinetics of sonication.^[76]^ This process was extended to produce seed nanofibers in a single step via synchronous sonication and CDSA. A solution of PFTMC_17_‐*b*‐PHPMA_439_ in DMSO/EtOH (5 : 95 *v*/*v*, 10 mg/mL, 1 wt %) was prepared and immediately sonicated for 4 h, yielding 30 nm±8 nm seed nanofibers **seeds2** (*Đ*_L_=1.07, Figure 3B). Synchronous sonication and CDSA of PFTMC_16_‐*b*‐PHPMA_73_ in DMSO/EtOH (5 : 95 *v*/*v*, 10 mg/mL, 1 wt %) for 4 hours also yielded 25 nm±9 nm seed nanofibers **seeds3** (*Đ*_L_=1.11, Figure 3C). Low‐dispersity length controlled PFTMC_17_‐*b*‐PHPMA_439_ nanofibers were then produced from seed nanofibers via living CDSA (Figure 3D–F, Figure S13, and Table S3–S4). As seed nanofibers retain active termini that can undergo epitaxial growth, further addition of PFTMC_17_‐*b*‐PHPMA_439_ unimer (20 mg/mL) to nanofiber **seeds1** (0.5 mg/mL) resulted in low‐dispersity nanofibers with a tuneable length that is related to the *m*_unimer_/*m*_seed_ (u/s) ratio (the mass ratio of added unimer vs seed nanofiber). Low dispersity morphologically pure nanofibers were observed for u/s ratios of up to 30 : 1, which correlated to length control between 27 nm±12 nm (*Đ*_L_=1.19) and 707 nm±155 nm (*Đ*_L_=1.05). A linear relationship was observed between the u/s ratio and the nanofiber length, indicative of a living CDSA process (*L*_n_=22.24(u/s) +44.98, R^2^=0.995). Above a u/s of 30 : 1, the nanofiber length deviated from linearity and little change in nanofiber length was observed. At a u/s of 40 : 1, 787 nm±175 nm nanofibers (*Đ*_L_=1.05) were obtained that exhibited a higher degree of fusion alongside the presence of nanospheres (Figure S14). At this concentration (10 mg/mL), nanofiber side‐to‐side fusion is favoured whilst the added unimer also undergoes self‐nucleation. To probe the height of the obtained nanofibers, atomic‐force microscopy (AFM) was conducted on 103 nm±20 nm PFTMC_17_‐*b*‐PHPMA_439_ **nanofibers1** (u/s=2, *Đ*_L_=1.04, Figure 4). A mean height of 8.2±0.5 nm was obtained from height analysis of four distinct nanofibers in the image. This value is between the two average values reported for the rectangular core of PFTMC‐*b*‐PEG nanofibers with a similar PFTMC DP_n_ and on average one chain‐fold per chain.^[9]^ For cytotoxicity studies, a larger quantity of PFTMC_17_‐*b*‐PHPMA_439_ nanofibers was produced from the living CDSA of **seeds2** yielding 70±14 nm **nanofibers2** (*Đ*_L_=1.04, Figure 3D). These nanofibers were transferred into water for cellular studies via dialysis, resulting in 15 mg of **nanofibers2** in 2.3 mL of water without any observable change in length (*L*_n_=70±14 nm, *Đ*_L_=1.04, Figure S15). Whilst stability and degradation studies are ongoing, other PFTMC nanomaterials have exhibited good stability in water for several years and were capable of enzymatic and hydrolytic degradation.[^55^, ^77^]

### Cytotoxicity Studies of PFTMC‐*b*‐PHPMA Nanofibers

PFTMC nanomaterials may be hydrolytically and enzymatically degraded, however it is imperative that any nanomaterials used in biomedical applications are non‐toxic. To ascertain whether PFTMC_17_‐*b*‐PHPMA_439_ nanofibers are cytocompatible, preliminary cell viability studies were conducted on 70±14 nm **nanofibers2**. This length of nanofiber was selected as it is an ideal length for nanomedicine applications[^4^, ^27^, ^78^] and is similar to the length of the precision nanofibers used in previous studies.[^9^, ^35^, ^55^] Our previous work demonstrated that neutral PFTMC_20_‐*b*‐PEG_490_ and PDHF‐*b*‐PEG nanofibers exhibited no cytotoxicity towards primary WI‐38 female foetal lung fibroblasts or HeLa female cervical carcinoma cells as assessed via 72 h alamarBlue™ and Calcein AM assays that assess reductive metabolism and cell viability respectively.[^9^, ^35^] In contrast, basic PFTMC_16_‐*b*‐PDMAEMA_131_ (PDMAEMA=poly(2‐(dimethylamino)ethyl methacrylate)) nanofibers were cytotoxic to the same cell lines in the same assays, with EC_50_ values between 497 and 876 nM.^[55]^ These results are in line with broader studies which suggest that neutral species exhibit minimal toxicity whilst basic species enable active cellular uptake and are more cytotoxic.[^6^, ^79^] We sought to ascertain whether neutral PFTMC_17_‐*b*‐PHPMA_439_ nanofibers behaved similarly to their PFTMC_20_‐*b*‐PEG_490_ counterparts. To ensure data is comparable with prior studies, experiments with PFTMC_17_‐*b*‐PHPMA_439_ nanofibers utilized identical cells and assays to those previous experiments.

**Figure 5:**
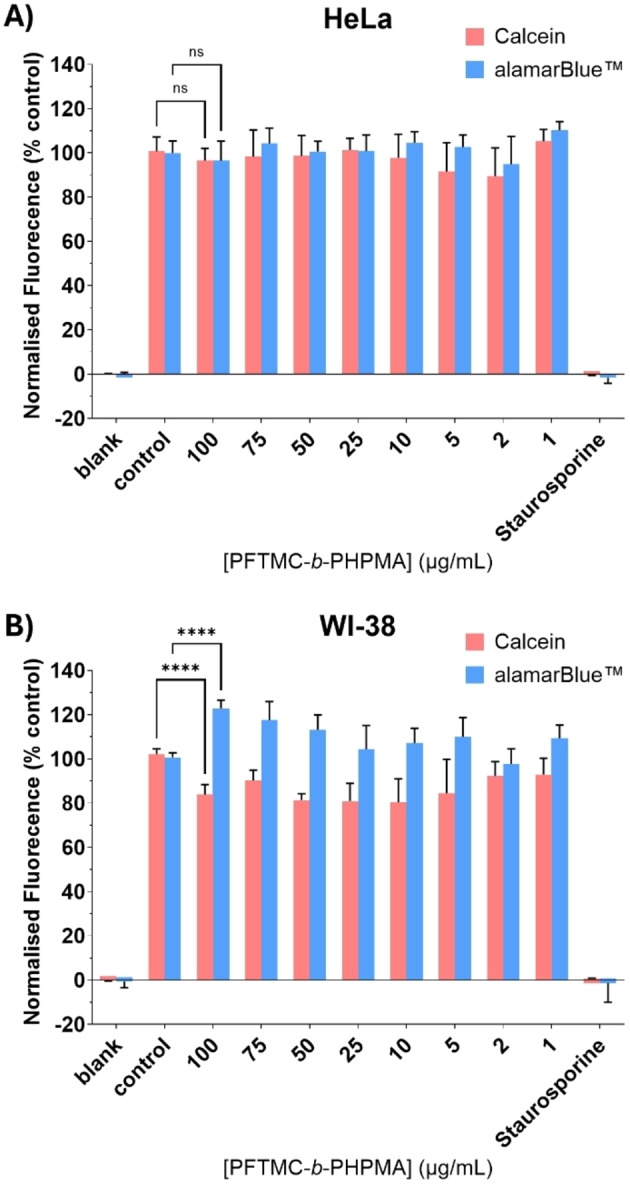
Cell viability studies of 70±14 nm PFTMC_17_‐*b*‐PHPMA_439_ nanofibers2 after 72 h incubation with (A) HeLa cervical carcinoma cells and (B) WI‐38 foetal lung fibroblasts. Cell viability was assessed via Calcein AM whilst reductive metabolism was assessed via alamarBlue™. The median value is plotted, with the 95 % confidence interval as error (n=4 for HeLa and 2 for WI‐38, 24 and 12 individual replicates respectively). ns and **** indicate no significance and a significance of p<0.0001 respectively as determined by two‐way analysis of variance with multiple comparisons (Dunnett correction).

HeLa cells were selected as an example of a rapidly dividing cancer cell line whilst WI‐38 cells were selected as an example of healthy primary cells. These cells were exposed to 0–100 μg/mL of 70±14 nm **nanofibers2** over a 72 h period after which reductive metabolism and cell viability were assessed via a combined alamarBlue™ and calcein AM assay. The results are displayed in Figure 5. For HeLa cells, no observable cytotoxicity was detected (Figure 5A) whilst for WI‐38 cells, some minor cytotoxic effects were observed with reduced cell viability and increased reductive metabolism, though cell viability (assessed via Calcein AM) remained above 80 % and reductive metabolism (assessed via alamarBlue™) remained between 100 % and 120 % of the control cells (Figure 5B and Tables S5–S8). Whilst these results indicate that PFTMC_17_‐*b*‐PHPMA_439_ nanofibers are more cytotoxic to cells than PFTMC_20_‐*b*‐PEG_490_ nanofibers, they remain far less toxic than PFTMC_16_‐*b*‐PDMAEMA_131_ nanofibers and exhibit minimal cytotoxicity at therapeutically relevant concentrations. Cell viability is just one aspect of understanding how nanomaterials interact with biological systems. For a more complete picture, we must also consider their cellular uptake and localization as non‐toxic materials may or may not undergo cellular association and internalization. Our prior studies on the cellular uptake of neutral PDHF‐*b*‐PEG nanofibers demonstrated that cellular uptake does not occur unless a targeting group for active transport is present.^[35]^ In contrast, the cellular localization and uptake of PFTMC‐*b*‐PEG nanofibers is unknown at present due to the challenges of functionalizing PEG. To overcome this issue, we were keen to harness the benefits of PET‐RAFT polymerization to produce functionalized PFTMC‐*b*‐PHPMA nanofibers that could be used as tools to study the cellular uptake and localization of neutral polycarbonate nanofibers.

### Preparation of Fluorescein‐Functionalized PFTMC‐*b*‐PHPMA Nanofibers and Nanospheres

We incorporated a fluorophore to enable confocal laser scanning microscopy (CLSM) and flow cytometry studies on the cellular uptake and localization of PFTMC‐*b*‐PHPMA. Several different strategies have been used to functionalize CDSA nanoparticles with fluorescent dyes. This typically involves the post‐polymerization functionalization of block copolymers either containing reactive side chains in the coronal block,[^31^, ^80^, ^81^] or via modification of the polymer end‐groups.[^18^, ^29^, ^35^, ^38^, ^55^] Fluorophores may be included either pre or post self‐assembly in these strategies. In this work, we sought to include the fluorophore within the RAFT polymerization of the corona as a simple, versatile method of producing fluorescent block copolymers that is complementary to the other methods. For this purpose, we utilized fluorescein O‐methacrylate (FlMA) as a relatively affordable fluorophore that costs much less than the bespoke dyes typically employed, however its emission is also more pH sensitive than other systems.

**Figure 6:**
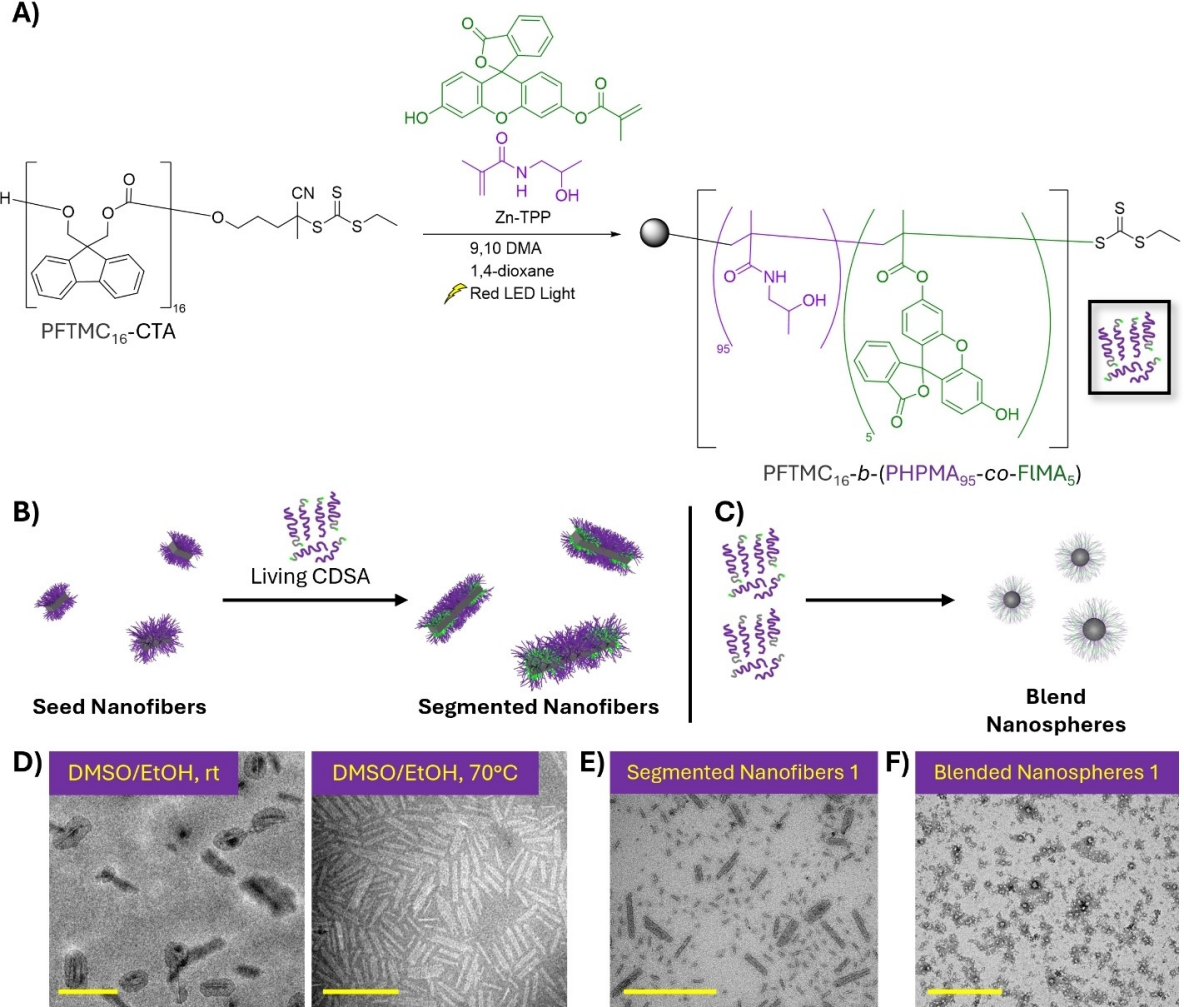
(A) Synthesis and (B–C) self‐assembly scheme of fluorescent PFTMC_16_‐*b*‐(PHPMA_95_‐*co*‐PFlMA_5_) into (B) nanofibers and (C) nanospheres. (D–F) TEM micrographs of (D) self‐nucleated PFTMC_16_‐*b*‐(PHPMA_95_‐*co*‐PFlMA_5_), and (E) segmented nanofibers 1 or (F) blended nanospheres 1 from co‐assembly with PFTMC_17_‐*b*‐PHPMA_439_. Scale=500 nm. All TEM samples were stained with uranyl acetate (3 wt % in EtOH).

PET‐RAFT of PFTMC_16_‐CTA with HPMA and fluorescein O‐methacrylate furnished PFTMC_16_‐*b*‐(PHPMA_95_‐*co*‐PFlMA_5_) (Figure 6). Whilst the dye inclusion and corona DP_n_ was lower than desired, this nonetheless resulted in fluorescent unimer (*M*_n_=1,600 Da; *Đ*_M_=6.58 via GPC, *M*_n_=16,100 Da via ^1^H‐NMR, *D*,=3.13×10^−7^ cm^2^/sec, Figure S16). Self‐assembly studies with PFTMC_16_‐*b*‐(PHPMA_95_‐*co*‐PFlMA_5_) in DMSO/EtOH (95 : 5 *v*/*v*) aged at 23 °C for 24 h led to fused nanofibers surrounded by unimer film, indicative of incomplete self‐assembly (Figure 6D).^[82]^ Annealing a separate sample at 70 °C for 15 minutes before aging for 24 h led to the formation of nanofibers that were primarily fused into 190±54 × 39±20 nm nanoplatelets (*Đ*_L_=1.08, *Đ*_W_=1.26, Figure 6D). These results indicate that the inclusion of a fluorescent dye typically has a large effect on the CDSA of block copolymers, regardless of the location of the fluorophore. We attempted to circumvent this issue by using PFTMC_17_‐*b*‐PHPMA_439_ nanofiber **seeds2** to produce 55±40 nm [PFTMC_16_‐*b*‐(PHPMA_95_‐*co*‐PFlMA_5_)]‐*m*‐[PFTMC_17_‐*b*‐PHPMA_439_]‐*m*‐[PFTMC_16_‐*b*‐(PHPMA_95_‐*co*‐PFlMA_5_)] triblock **segmented nanofibers 1** with an average of 30 nm PFTMC_17_‐*b*‐PHPMA_439_ central segments and 25 nm PFTMC_16_‐*b*‐(PHPMA_95_‐*co*‐PFlMA_5_) terminal segments (1 : 1 mass ratio, *Đ*_L_=1.52, *W*_n_=15±9 nm, *Đ*_W_=1.36). After transfer into water, TEM analysis revealed the presence of individual nanofibers as well as small nanoplatelets that consisted of fused nanofibers (Figure 6E). A *D*_h_ of 208±5 nm was obtained via DLS in NaCl (5 mM) that is consistent with non‐aggregated platelets and small clusters of nanofibers (Figure S17A).^[83]^ ζ‐potential values for **segmented nanofibers 1** in NaCl (5 mM) were negative at −9.3±0.4 mV, consistent with the presence of anionic fluorescein species (Figure S17B).^[84]^

To enable comparisons between nanofibers and nanospheres in cellular uptake studies, a mixture of PFTMC_17_‐*b*‐PHPMA_439_ and PFTMC_16_‐*b*‐(PHPMA_95_‐*co*‐PFlMA_5_) unimer (1 : 2 ratio) were mixed in DMSO and dialysed into water, yielding **blended nanospheres 1** (number‐average diameter, *D*_n_=16±4 nm, *Đ*_D_=1.06, Figure 6F) which also contained a small number of nanofibers (Figure S18). The **blended nanospheres 1** exhibited a *D*_h_ of 179±13 nm and a ζ‐potential value of −6.5±0.8 mV which is consistent with small clusters of aggregated nanospheres that contain anionic fluorescein species (Figure S17C–D).

### Cellular Uptake of Fluorescein‐Functionalized PFTMC‐*b*‐PHPMA Nanofibers and Nanospheres

**Figure 7:**
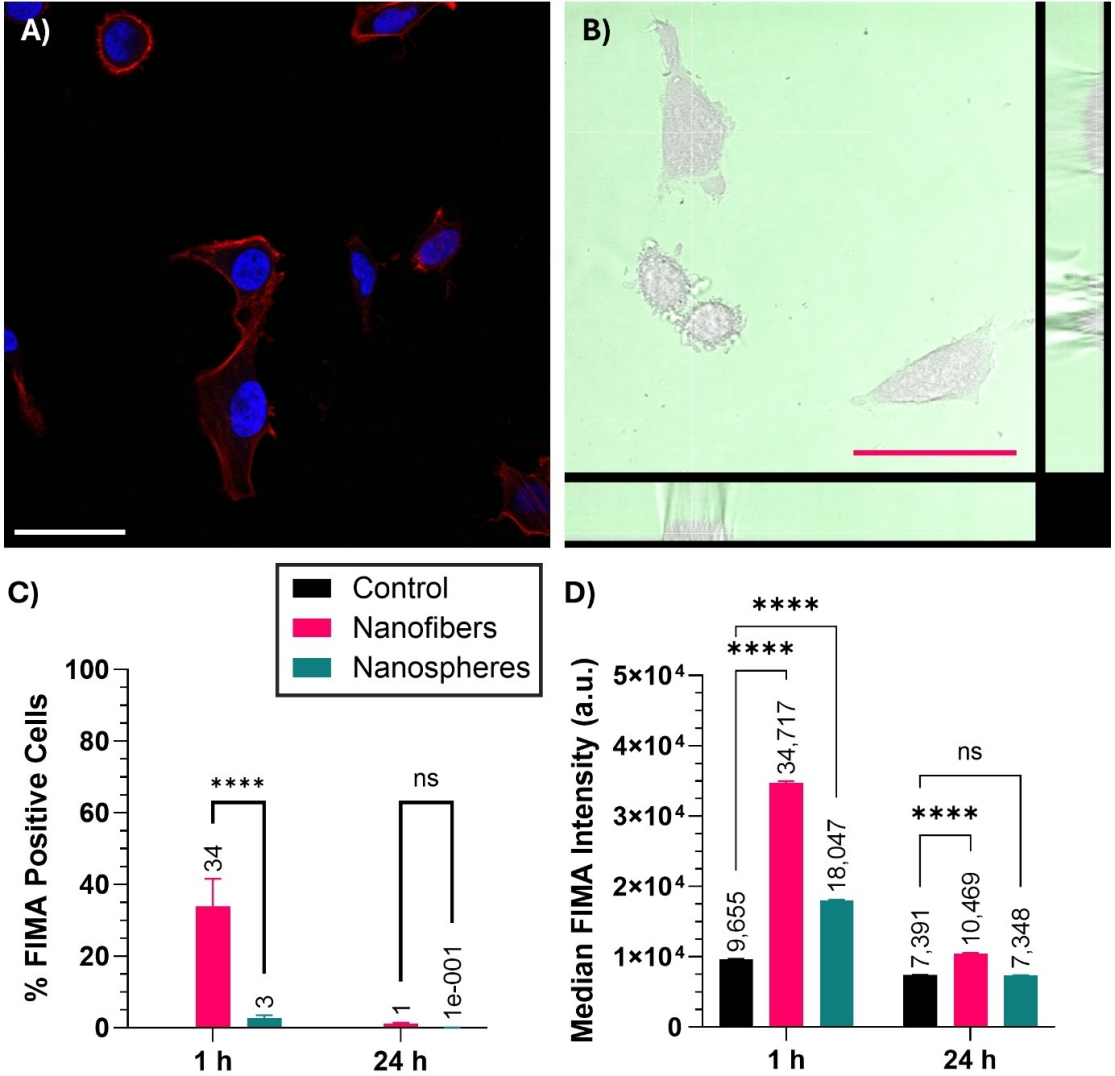
Cellular uptake of fluorescent PFTMC‐*b*‐PHPMA segmented nanofibers 1 assessed via CLSM with (A) fixed HeLa cells incubated with segmented nanofibers 1 (green channel) and labelled with 4′,6‐diamidino‐2‐phenylindole (DAPI, nuclear stain, blue channel) and Alexa Fluor™ 594 Phalloidin (F‐actin stain, red channel), and (B) live HeLa cells (brightfield) with the segmented nanofibers 1 supernatant present (green channel). Scale=50 μm. (C–D) Flow cytometry of U‐87 MG cells as (C) the % of fluorescein positive cells and (D) the median fluorescence intensity upon exposure to segmented nanofibers 1 and blended nanospheres 1. Error=95 % confidence interval. ns and **** indicate no significance and a significance of p <0.0001 respectively as determined by two‐way analysis of variance with multiple comparisons (Tukey correction).

As cytotoxicity studies were conducted using HeLa cells, initial experiments studied the cellular uptake of fluorescent PFTMC‐*b*‐PHPMA **segmented nanofibers 1** into HeLa cells via CLSM (Figure 7A and B). HeLa cells were incubated with 100 μg/mL of **segmented nanofibers 1** for 1 hour before the cells were fixed, the nucleus was stained with DAPI (40,6‐diamidino‐2‐ phenylindole), the F‐actin was stained with Alexa Fluor™ 633 Phalloidin and the cells were imaged via CLSM. No intracellular fluorescence was observed for the fluorescein fluorophore (λ_ex_=488 nm, λ_em_=498–551 nm) compared to control cells, which indicated that PFTMC‐*b*‐PHPMA **segmented nanofibers 1** were not internalized into HeLa cells after a 1 hour exposure (Figure 7A). These results were confirmed by live cell imaging studies of HeLa cells after 45 minutes incubation with **segmented nanofibers 1** (50 μg/mL) with the fluorescent nanofiber supernatant left in place (Figure 7B). Distinct, uniform fluorescence was observed in all z‐stack images around the cells, however no fluorescence was observed within the cells. Live cell imaging of HeLa cells exposed to **segmented nanofibers 1** (100 μg/mL, 45 minutes exposure) after the supernatant had been replaced also contained no observable fluorescence over background cellular autofluorescence. Together, these results indicate that neutral PFTMC‐*b*‐PHPMA nanofibers do not undergo internalization into HeLa cells. This result is in agreement with our results from neutral 85 nm PDHF‐*b*‐PEG nanofibers^[35]^ and results from Feng and coworkers, who demonstrated that neutral 156 nm oligo(p‐phenylenevinylene)‐*b*‐PEG nanofibers did not undergo internalization into HeLa cells after 5 hours incubation.^[36]^

To study whether this phenomenon was specific to the nanofiber morphology or to HeLa cells as opposed to a more broadly applicable phenomenon, we incubated 10 μg/mL of fluorescent PFTMC‐*b*‐PHPMA **segmented nanofibers 1** and **blended nanospheres 1** with U‐87 MG glioblastoma cells for 1 or 24 hours before analysing their cellular association via flow cytometry. We selected U‐87 MG human male epithelial glioblastoma cells as our prior work on DNA delivery has demonstrated that short (<100 nm) PFTMC‐*b*‐PDMAEMA nanofibers are capable of potent DNA delivery and hence cellular uptake with this cell type. After incubation for the desired time, dead cells were stained with propidium iodide and the cells were analysed via flow cytometry. The results were gated for cells, single cells, live cells and then for fluorescein fluorescence (λ_ex_=488 nm, λ_em_=525/40 nm) using the open‐source Cytoflow software (Figures S19–S20 and Tables S9–S12).^[85]^

Propidium iodide labelling of dead cells indicated that cell viability remained above 99 % for all samples. After 1 h incubation, a modest increase in fluorescein positive cells was observed for **segmented nanofibers 1**, with 34 % of cells being fluorescein positive compared to the control. This equated to a 351 % increase in median fluorescence intensity (MFI) compared to control cells. In contrast, only 3 % of cells were positive for **blended nanospheres 1**, equating to a 185 % increase in MFI. After 24 hours incubation, limited cellular uptake was observed for both **segmented nanofibers 1** and **blended nanospheres 1**. Only 3 % of cells were fluorescein positive for **segmented nanofibers 1**, equating to a 144 % increase in MFI. In contrast, no observable difference in MFI or % fluorescein positive cells was observed for **blended nanospheres 1** compared to the control. These results demonstrate that whilst both PFTMC‐*b*‐PHPMA nanofibers and nanospheres do not interact with U‐87 MG cells, nanofibers nonetheless exhibited a modest cellular association within 1 hour of incubation. As the fluorescein MFI decreased over time, both nanofibers and nanospheres appear to be degraded or eliminated from cells within 24 hours of addition.

The increased interaction of PFTMC‐*b*‐PHPMA nanofibers with cells is in agreement with literature studies that suggest that anisotropic nanoparticles with rigid cores are able to more effectively undergo cellular internalization.^[26]^ Thus, the nanofiber shape uniquely enables neutral stealth nanoparticles to associate with cells, albeit in limited quantities, whilst the equivalent spherical nanoparticles do not interact with the cells at all. It is important to note that flow cytometry does not differentiate between nanoparticles that are associated with the membrane externally and nanoparticles that have been internalized into the cell. Therefore, the present study contains no evidence to suggest that PFTMC‐*b*‐PHPMA nanoparticles are internalized into cells, they may merely be associated with the cell surface. A limitation of the present study is that PFTMC‐*b*‐PHPMA **segmented nanofibers 1** contain a mixture of nanoplatelets and nanofibers, which whilst similar in overall size and relatively low dispersity, prevent definitive conclusions about which morphology is responsible for the observed cellular association. Furthermore, whilst FlMA is an effective and affordable fluorescent tag, the pH sensitivity of the fluorescein emission complicates quantitative comparisons between FlMA labelled samples. Further studies are therefore required to probe the differences between nanoplatelets, nanofibers, and nanospheres prepared via CDSA using alternative fluorophores to eliminate potential sources of error. Nevertheless, these results demonstrate that PFTMC‐*b*‐PHPMA nanofibers, nanoplatelets and nanospheres exhibit stealth properties, ideally positioning them for use in targeted biomedical applications. Furthermore, these results confirm the general trend that neutral polymer nanofibers with short lengths (<200 nm) and crystalline cores do not undergo significant cellular internalization unless a targeting group for active transport is present.[^18^, ^25^, ^35^, ^36^]

## Conclusions

In this work, we have synthesized a new class of stealth polymer nanofibers via a combination of ROP, PET‐RAFT polymerization and CDSA. This work demonstrates that PFTMC is a robust core‐forming block for CDSA that can generate precision nanofibers from an ever‐expanding repertoire of block copolymers. We have brought the benefits of PET‐RAFT polymerization to neutral precision nanofibers by incorporating the stealth polymer PHPMA into PFTMC block copolymers, enabling their versatile functionalization in contrast to PFTMC‐*b*‐PEG. As a proof of concept, we have incorporated a fluorescent monomer, FlMA into the coronal PHPMA block via PET‐RAFT, generating a versatile and simple route to fluorescent polymer nanofibers.

PFTMC‐*b*‐PHPMA is capable of self‐assembling into precision nanofibers under a broad range of conditions via CDSA. Low length‐dispersity nanofibers were obtained in a single‐step process due to the rapid rate of self‐nucleation, with the coronal block length found to affect the resulting nanofiber length. This is a new strategy for the precise synthesis of low length‐dispersity nanofibers via CDSA and enables access to precision stealth nanofibers with biologically relevant lengths. Sonication led to the formation of seed nanofibers within 30 minutes that reached a critical length after 6 hours and these seeds were used for living CDSA studies with length control from ca. 30 nm to over 700 nm. Low‐length‐dispersity nanofibers were transferred into water and used for preliminary studies on their cytotoxicity and cellular uptake.

PFTMC‐*b*‐PHPMA nanofibers exhibited little cytotoxicity as well as limited cell uptake and were degraded or excreted from cells within 24 hours. In contrast, PFTMC‐*b*‐PHPMA nanospheres exhibited no interactions with cells in the same experiments. PFTMC‐*b*‐PHPMA nanofibers and nanospheres therefore retained the stealth properties of PHPMA and serve as complementary systems depending on the end application. PFTMC‐*b*‐PHPMA nanofibers show promise as therapeutic delivery systems due to their increased cellular association compared to nanospheres that stems from the unique combination of their 1D shape and core crystallinity. To this end, future work will focus on the functionalization of PFTMC‐*b*‐PHPMA nanofibers with therapeutic cargo and targeting groups for active transport into cells. Overall, this work highlights the importance of considering the shape‐mediated interactions with cells when designing nanomaterials for biomedical applications and reinforces the important role that precision stealth nanofibers can play in driving the cellular association of stealth polymer nanoparticles.

## Conflict of Interests

Dr. Willerth is the C.E.O. and co‐founder of Axolotl Biosciences.

1

## Supporting information

As a service to our authors and readers, this journal provides supporting information supplied by the authors. Such materials are peer reviewed and may be re‐organized for online delivery, but are not copy‐edited or typeset. Technical support issues arising from supporting information (other than missing files) should be addressed to the authors.

Supporting Information

## Data Availability

The data that support the findings of this study are available in the supplementary material of this article.
