# Supplementary material for: Precision Stealth Nanofibers via PET‐RAFT Polymerisation: Synthesis, Crystallization‐driven Self‐assembly and Cellular Uptake Studies
Source: Chemistry. 2025 Mar 12;31(22):e202500108. doi: 10.1002/chem.202500108 (PMC12015387; doi:10.1002/chem.202500108)
Supplement: Supplementary file 1 — Supporting Information [file CHEM-31-e202500108-s001.pdf]

Chemistry–A European Journal

Supporting Information

Precision Stealth Nanofibers via PET-RAFT Polymerisation: Synthesis, Crystallization-driven Self-assembly and Cellular Uptake Studies

Steven T. G. Street,* Ekaterina Shteinberg, Juan Diego Garcia Hernandez, Hayley C. Parkin, Robert L. Harniman, Stephanie Willerth,* and Ian Manners

Supplementary Information

Precision Stealth Nanofibers via PET-RAFT Polymerisation: Synthesis, Crystallization-Driven Self-Assembly and Cellular Uptake Studies

Steven T. G. Street,[a-d] Ekaterina Shteinberg,[e] Juan Diego Garcia Hernandez,[a] Hayley C. Parkin,[b-c] Robert L. Harniman,[a] Stephanie Willerth[c,e-f]* and Ian Manners[a-c]†*

In memory of Prof. Ian Manners, an exceptional scientist, collaborator, and mentor who is sorely missed.

[a] S. T. G. Street, J. D. Garcia Hernandez, R. L. Harniman, I. Manners: School of Chemistry, University of Bristol, Bristol BS8 1TS, United Kingdom

[b] S. T. G. Street, H. C. Parkin, I. Manners: Department of Chemistry, University of Victoria, Victoria, BC V8W 3V6, Canada

[c] S. T. G. Street, H. C. Parkin, S. Willerth, I. Manners: Centre for Advanced Materials and Related Technology (CAMTEC), University of Victoria, 3800 Finnerty Rd, Victoria, BC, V8P 5C2, Canada

[d] S. T. G. Street (Current Address): School of Chemistry, University of Birmingham, Edgbaston B15 2TT, United Kingdom

[e] E. Shteinberg, S. Willerth: Department of Mechanical Engineering, Division of Medical Sciences, University of Victoria, Victoria, BC V8W 2Y2, Canada

[f] S. Willerth: School of Biomedical Engineering, University of British Columbia, Vancouver, BC V6T 1Z4, Canada

† Deceased 2023-12-03.

* Corresponding Author: s.t.g.street@bham.ac.uk
willerth@uvic.ca

Contents

Contents S1
General Experimental Considerations S3
Instrumentation S4
Matrix-assisted laser desorption/ionization time of flight mass spectrometry (MALDI-TOF MS) S4

Size exclusion chromatography (SEC) S4
NMR Spectroscopy S4
Ultrasonication S5
Transfer of samples into water S5
Transmission electron microscopy (TEM) S5
Atomic Force Microscopy S6
Dynamic Light Scattering (DLS) S6
ζ-Potential Measurements S7
Confocal Laser Scanning Microscopy (CLSM) S7
Flow Cytometry S8
Synthetic Procedures S9
$PFTMC_{17}$-CTA S9
$PFTMC_{17}$-*b*-$PHPMA_{439}$ S10
$PFTMC_{16}$-*b*-$PHPMA_{73}$ S11
$PFTMC_{16}$-*b*-($PHPMA_{95}$-*co*-$PFlMA_{5}$) S12
Self-assembly procedures S13
General procedure for the CDSA of PFTMC-*b*-PHPMA S13
Example procedure for the self-assembly of $PFTMC_{17}$-*b*-$PHPMA_{439}$ S14
General Procedure for the preparation of seed nanofibers from disperse nanofibers S14
Example procedure for the preparation of seed nanofibers from disperse nanofibers S14
General Procedure for the one-step preparation of seed nanofibers S14
Example Procedure for the one-step preparation of seed nanofibers S15
General procedure for the preparation of low dispersity nanofibers from seed nanofibers using the seeded-growth method (living CDSA) S15
Example procedure for the preparation of low dispersity nanofibers from seed nanofibers S15
General procedure for the transfer of low dispersity nanofibers into water S15
Example procedure for the transfer of low dispersity nanofibers into water S16
General procedure for the preparation of segmented nanofibers S16
Procedure for the preparation of segmented nanofibers 1 .... S16
General procedure for the preparation of nanospheres via dialysis S17
Example procedure for the preparation of blend nanospheres via dialysis S17
Characterization data for individual samples used in this work S18
Cell culture protocols S19
Cell Viability Assays S19
Cell Uptake Assays S19

Sonication Kinetics Study S20
Supplementary Figures S21
References S55

General Experimental Considerations

All reagents and solvents were purchased from Sigma-Aldrich, Acros, Fluka, Fisher Chemical and Alfa Aesar, and used as received unless otherwise noted. All reactions were carried out in an MBraun MB150B-G glove box under nitrogen atmosphere or using standard Schlenk line techniques. Solvents used for self-assembly were HPLC grade and were filtered through PTFE, nylon or cellulose membranes with a pore size of 200 nm before use. Anhydrous solvents were obtained using a modified Grubbs system of alumina columns manufactured by Anhydrous Engineering. RAFT polymerizations were performed in custom-made Schlenk-vials to fit dry heating blocks. Ring-opening polymerization (ROP) reactions were conducted in oven-dried glass vials. 1,8-Diazabicyclo[5.4.0]undec-7-ene (DBU) was dried over CaH_2, and purified by distillation under reduced pressure. Reagents for ROP were dried via vacuum desiccation over phosphorus pentoxide for ~2 days before being brought into the glovebox. Reactions were monitored by thin layer chromatography (TLC) on Kieselgel 60 F_{254} (Merck). Aromatic compounds were detected with UV light (254 or 365 nm), and amines were detected by staining with ninhydrin. HeLa and WI-38 cells were purchased from the American Type Culture Collection (ATCC) through LGC Standards (UK). U-87 MG cells were purchased from were purchased from the ATCC through Cedarlane Corporation (Canada). Cell culture media and additives were purchased from Gibco (Thermo Fischer Scientific). The Dulbecco's Minimal Essential Medium (DMEM) formulation contained high glucose (4.5 g/L), Sodium Pyruvate (0.11 g/L), GlutaMAX™, and Phenol Red (15 mg/L), and was missing HEPES (catalogue number: 10569044). The Minimal Essential Medium (MEM) formulation contained GlutaMAX™, and Phenol Red (10 mg/L), and was missing HEPES (catalogue number: 41090101). Phosphate Buffered Saline (PBS) contained NaCl (9 g/L), KH_2PO_4 (144 mg/L) and Na_2HPO_4-7.H2O (795 mg/L, catalogue number: 10010049). TrypLE Express™ was provided with EDTA (458 mg/L) and without Phenol Red (catalogue number: 12604021).

Instrumentation

Matrix-assisted laser desorption/ionization time of flight mass spectrometry (MALDI-TOF MS)

MALDI-TOF MS measurements were performed using a Bruker Ultraflextreme running in reflector mode. MALDI-TOF samples were prepared by depositing approximately 1 μL of the sample (2 mg/mL in THF) onto a stainless-steel sample plate, followed by the deposition of approximately 2 μL of *trans*-2-[3-(4-*tert*-butylphenyl)-2-methyl-2-propenylidene]malononitrile matrix (20 mg/mL in THF), and the sample was allowed to dry in air. If the crystalline matrix could not be observed on the plate, a further aliquot of matrix was added, and the sample dried in air until crystallization was observed. For all samples, a second spot was also prepared with the addition of sodium trifluoroacetate (20 mg/mL in THF) to supress K^+ adducts. The best spectrum was selected for each sample (with/without sodium trifluoroacetate). This was with sodium trifluoroacetate for $PFTMC_{17}$-CTA.

Size exclusion chromatography (SEC)

Size exclusion chromatography (SEC) was performed in **DMF** on an Agilent 1260 Infinity II LC system equipped with a Wyatt DAWN HELEOS II multi-angle laser light scattering (MALLS) detector, a Wyatt Optilab T-rEX differential refractive index detector, an Agilent 1260 Infinity II WR diode array detector, an Agilent guard column (PLGel 5 μM, 50 × 7.5 mm) and two Agilent Mixed-C columns (PLGel 5 μM, 300 × 7.5 mm). The mobile phase was DMF (HPLC grade) containing 5 mM NH_4BF_4 at 50 °C at flow rate of 1.0 mL min^{-1}. Number average molecular weights (M_n), weight average molecular weights (M_w) and dispersities ($Đ_M = M_w/M_n$) were determined using Wyatt ASTRA v7.1.3 software against poly(methyl methacrylate) (PMMA) standards. For molecular weight determination *via* MALLS, dn/dc values were either measured by differential refractometry or calculated from the SEC chromatograms assuming 100% mass elution from the columns. Samples were prepared at 2 mg/mL in eluent and filtered through a Ministart SRP 15 filter (polytetrafluorethylene membrane, pore size = 0.45 µm).

NMR Spectroscopy

^{1}H and ^{13}C NMR spectra were obtained at 25 °C in the solvent specified with Varian or Bruker spectrometers (some equipped with a cryoprobe), operating at the field strengths listed. Chemical

shifts are quoted in parts per million with spectra referenced to the residual solvent peak. Multiplicities are abbreviated as: br (broad), s (singlet), d (doublet), t (triplet), q (quartet), p (pentet), m (multiplet) and *app.* (apparent) or combinations thereof. Assignments of ^{1}H-NMR and ^{13}C-NMR signals were made where possible, using COSY, HSQC and HMBC experiments. DOSY NMR experiments were processed using the 'Bayesian DOSY Transform' function of MestreNova.

Ultrasonication

Micelle sonication was carried out using a Hielscher UP100H sonication probe (100W output power) or a Fisherbrand 112xx series advanced ultrasonic cleaner (FB-11203) (37 MHz at 80 % power).

Transfer of samples into water

Samples were transferred into water through dialysis, using dialysis membranes from Sigma Aldrich with a molecular weight cut-off (MWCO) of 12,000 – 14,000 Da. Samples were manually shaken for ~10 s, and then vortex mixed for ~10 s before transfer into the dialysis membrane. Samples were transferred at 2× the desired final concentration, with the volume being corrected gravimetrically post-dialysis.

Transmission electron microscopy (TEM)

TEM images were obtained on either a JEOL 1400 microscope with a Gatan Orius SC1000 CCD camera, operated at 120 kV or a JEOL 1011 microscope with an 11 Megapixel CCD camera, operated at 80 kV. Samples were prepared by drop casting 1.5 µL of the micelle solution (0.1 – 10 mg/mL) onto a carbon coated copper grid. Negatively stained samples were additionally drop cast with uranyl acetate in EtOH (8 µL, 3 wt%). Copper grids (400 or 500 mesh) were purchased from Agar Scientific and carbon films (ca. 6 nm) were prepared on mica sheets by carbon sputtering with an Agar TEM Turbo Carbon Coater or a Leica ACE 600 carbon coater. The carbon films were deposited onto the copper grids by floatation on water using the Smith Grid Coating Trough (Ladd Research Industries) and the carbon coated grids were allowed to dry in air.

For micelle contour length analysis, a minimum of 200 micelles were traced manually using the Fiji (ImageJ) software package developed at the US National Institute of Health. The images used for the contour length analysis are not necessarily the images presented, but were instead

other images of the same sample (usually at a more optimum magnification for the analysis of 200 particles). For some samples, multiple images were used for contour length analysis. The number average micelle length (L_n), width (W_n) or diameter (D_n) and weight average micelle length (L_w), width (W_w) or diameter (D_w) were calculated using eq. S1-2 from measurements of the contour lengths/widths (L_i) of individual micelles, where Ni is the number of micelles of length Li, and n is the number of micelles examined in each sample. The distribution of micelle lengths/widths (termed $Đ$) is characterized by both L_w/L_n ($Đ_L$) or W_w/W_n ($Đ_W$) or D_w/D_n ($Đ_D$) and σ (standard deviation, σ_L, σ_W and σ_D).

$$L_n = \frac{\sum_{i=1}^{n} N_i L_i}{\sum_{i=1}^{n} Ni} \qquad L_w = \frac{\sum_{i=1}^{n} N_i L_i^2}{\sum_{i=1}^{n} NiLi} \qquad \text{(eq. S1-2)}$$

Atomic Force Microscopy

Atomic-force microscopy (AFM) analyses were performed in ambient conditions using a Bruker Multimode VIII atomic force microscope equipped with a ScanAsyst-HR fast scanning module and a ScanAsyst-Air-HR probe (tip radius, 2 nm), utilising peak force feedback control.

Samples for AFM were prepared by either drop casting 8 µL of micelle colloidal solution onto freshly cleaved mica and drying with a gentle stream of nitrogen, or prepared on carbon-coated TEM grids using the procedure mentioned above for TEM sample preparation. For PFTMC-*b*-HPMA, superior results were obtained using TEM grids, so this is the data presented. Data was processed and visualized using Gwyddion (http://gwyddion.net/), using the levelling and align rows tools to subtract the background.

Dynamic Light Scattering (DLS)

Dynamic light scattering (DLS) experiments were carried out using a Zetasizer Pro (Malvern Panalytical). Samples were diluted to 100 µg/mL in 5 mM NaCl buffer, with each cuvette containing 700 µL of micelle solution. The cuvette used was a Malvern folded capillary zeta cell (DTS1070, 700 µL volume). A minimum of five measurements per sample were taken. The correlation function was acquired in real time and analysed with a function capable of modelling multiple exponentials (Cumulant analysis). This process enabled the diffusion coefficients for the component particles to be extracted, and these were subsequently expressed as effective hydrodynamic diameter using the Stokes-Einstein relationship for coated nanospheres in H_2O,

with core properties of polystyrene latex (RI = 1.590, Absorption = 0.010, dispersant RI = 1.33, dispersant viscosity = 0.887, dispersant dielectric constant 78.5). As these measurements assume that the particles are spherical, measurements of nanofiber size via DLS are not absolute, but still provide a useful method for monitoring the colloidal stability of the samples and providing relative comparisons.

ζ-Potential Measurements

ζ-potential measurements were recorded on a Zetasizer Pro (Malvern Panalytical), following the Smoluchowski approximation at 25 °C. Samples were diluted to 100 µg/mL in 5 mM NaCl buffer, with each cuvette (DTS1070) containing 700 µL of micelle solution. A minimum of five measurements per sample were taken, each consisting of between 10 and 100 cycles per run. The average *ζ*-potential was calculated from the individual measurements taken, with error represented as σ.

Confocal Laser Scanning Microscopy (CLSM)

CLSM imaging was performed in the Wolfson Bioimaging Facility at the University of Bristol on a Lecia SP8 AOBS confocal laser scanning microscope attached to a Lecia DM I6000 inverted epifluorescence microscope with 'Adaptive Focus Control' to correct focus drift during time-courses (BBSRC Alert 13 capital grant (BB/L014181/1). All images were taken at 37 °C using a 63 × 1.4 oil-immersion lens. For multi-fluorophore imaging, each fluorphore was analysed independently in a sequential fashion. For cell samples, the nucleus was labelled with 4′,6-diamidino-2-phenylindole (DAPI) and the F-actin was labelled with Alexa Fluor™ 594 Phalloidin. For DAPI, the excitation laser was operated at 405 nm, and confocal images were obtained using a digital detector with an observation window between 415-478 nm. For fluorescein O-methacrylate, the excitation laser was operated at 488 nm, and confocal images were obtained using a digital detector with an observation window between 502-555 nm. For Alexa Fluor™ 594 Phalloidin, the excitation laser was operated at 594 nm, and confocal images were obtained using a digital detector with an observation window between 571-670 nm. Sampes were imaged as a Z-stack with the images shown representing a middle image from the stack. The images and Z-stacks were processed using LAS X (Lecia) and Fiji software (ImageJ).

Cell samples were cultured on microscope coverslips before being labelled with the stains according to their respective protocols and transferred to a clean microscope slide that contains

one drop of Prolong Gold antifade mountant (Thermo Fisher, catalogue number: P36930). The coverslip was sealed in place with clear nail polish.

Flow Cytometry

Flow cytometry was conducted at the University of Victoria in the CAMTEC Biomedical Core, on a Beckman Coulter CytoFLEX flow cytometer (Beckman Coulter Life Sciences) using the plate reader mode. Optical filters were arranged according to instrument specifications to enable Fluorescein O-methacrylate and propidium iodide (PI) analysis. The forward-scatter gain was 114 and the side-scatter gain was 89, with an automatic threshold based on forward-scatter. The 488 nm laser was used to excite Fluorescein O-methacrylate and PI, with the emission being measured using a 525/40 and a 610/20 nm band pass filter (BP) at a gain of 165 and 410 respectively. A total of three independent repeats were conducted for each sample, with a minimum of 10,000 cells counted for each repeat. The cells were gated for cells, then single cells, then live cells, before being gated for fluorescein O-methacrylate positive cells. The resulting data was analysed using the open-source 'Cytoflow' software (https://cytoflow.github.io/, Teague Lab, Trinity University, TX, USA).[1] The raw flow cytometry data is freely available from the authors upon request.

For sample preparation, U-87 MG cells were seeded at a density of 10^5 –10^7 cells/mL (10^4 –10^6 cells/well) in a 96-well plate. The cells were left to attach to the plate overnight, before incubation with the nanoparticles (10 µg/mL in cell media with foetal bovine serum [FBS, 10% *v*/*v*]) for the desired time. After incubation was complete, the supernatant was aspirated and the cells were detached using Trypsin-EDTA (indicator free, 100 µL, incubation 0.25%). After 20 mins incubation with Trypsin-EDTA, complete cell detachment was confirmed via microscopy before PI (0.5 µL of a of 400× solution) was added to each well 1 min prior to flow cytometry analysis.

Synthetic Procedures

PFTMC$_{17}$-CTA

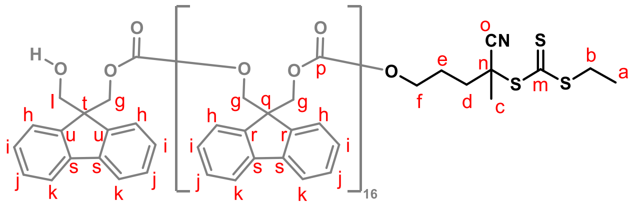

According to the literature procedure,[S[2]] to a solution of 2-cyano-5-hydroxypentan-2-yl ethyl carbonotrithioate (**CTA-OH**, 63.0 mg, 0.253 mmol, 1.0 eq)[S[3]] in anhydrous CH_2Cl_2 (500 µL), 1,8-Diazabicyclo[5.4.0]undec-7-ene (30 µL, 0.202 mmol, 0.8 eq) was added and stirred at rt for 15 mins. In a separate oven dried glass vial, FTMC (1.275 g, 5.05 mmol, 20 eq) and anhydrous CH_2Cl_2 (6 mL) was added and stirred at rt until all of the FTMC had dissolved, at which point it was added to the first solution of initiator and catalyst. The reaction mixture was then stirred at room temperature (23 °C) for 20 mins, until complete consumption of the starting material was observed via NMR. The crude reaction mixture was quenched by the addition of benzoic acid (50 mg) and purified by precipitation into ice-cold diethyl ether three times, before being dried *in vacuo* to yield **PFTMC$_{17}$-CTA** as a yellow solid (1.02 g, 76 %). Analysis of the ^{1}H-NMR integrals of Hf, Hl and Hb revealed a >99 % end capping with the CTA and a 90 % retention of the trithiocarbonate. **^{1}H-NMR** (500 MHz, CD_2Cl_2) δ 7.82 – 7.68 (40H, m, Hh), 7.52 (40H, dd, J = 31.5, 8.4 Hz, Hk), 7.46 – 7.34 (40H, m, Hi), 7.34 – 7.20 (40H, m, Hj), 4.53 – 4.15 (80H, m, Hg), 4.08 (2H, t, J = 6.1 Hz, Hf), 3.71 (2H, d, J = 5.9 Hz, Hl), 3.32 (2H, q, J = 7.6 Hz, Hb), 2.29 – 2.22 (1H, m, Hd), 2.08 – 1.98 (1H, m, Hd), 1.91 – 1.83 (2H, m, He), 1.81 (3H, s, Hc), 1.32 (3H, t, J = 7.4 Hz, Ha) DP_n = 20; **^{13}C-NMR** (126 MHz, CD_2Cl_2) δ 155.0 (Cp), 154.4 (Cp), 144.2 (Cr), 143.9 (Cu), 141.3 (Cs), 129.3 (Ci), 128.1 (Cj), 125.5 (Ck), 120.9 (Ch), 69.5 (Cg); **DOSY-NMR** (500 MHz, CD_2Cl_2) $D = 1.11 \times 10^{-5}$ cm^2/seconds; **MALDI-TOF MS** for $C_{281}H_{219}NO_{52}S_3Na^+$ $[M_{17}+Na]^+$, calculated: 4,557.4; found: 4,557.5, DP_n = 17, $Đ_M$ = 1.05; **GPC** (*n*-Bu_4NBr/THF, PS standard): M_n = 4,700 g/mol, $Đ_M$ = 1.14.

PFTMC$_{17}$-*b*-PHPMA$_{439}$

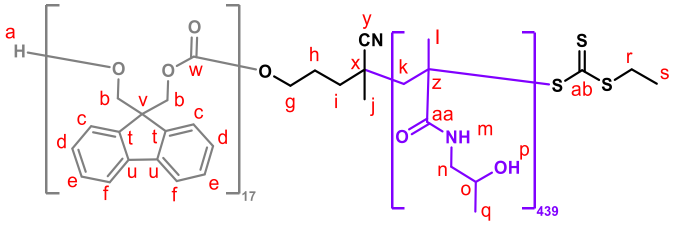

To a solution of 9,10-dimethylanthracene (DMA, 10.0 mg, 0.049 mmol, 3.2 eq) and 2-hydroxypropyl methacrylamide (HPMA, 322 µL, 2.25 mmol, 150 eq) in dioxane (stabilizer-free, 1 mL), **PFTMC$_{17}$-CTA** (63.1 mg, 0.013 mmol, 1.0 eq) in dioxane (stabilizer-free, 1 mL) was added. To this solution, a solution of 5,10,15,20-tetraphenyl-21H,23H-porphine zinc (ZnTPP, 142 µL of a 1 mg/mL solution in dioxane, 0.2 µmol, 0.014 eq) was added. The resulting mixture was stirred in a batch photoreactor prepared according to the designs of Corrigan et. al.[4] under red LED light irradiation. After 24 h, the reaction mixture has solidified into a light pink gel. To the resulting gel, MeOH was added until it dissolved into a light pink solution (5 mL). The crude reaction mixture was purified by precipitation from MeOH into ice-cold diethyl ether three times, at which point it was noted that the supernatant remained pink, indicating the presence of ZnTPP, before the resulting solid was dried *in vacuo* to yield PFTMC$_{17}$-*b*-PHPMA$_{168}$ as an off-white powder (286 mg, 72 %).

Residual PFTMC homopolymer was removed by washing PFTMC$_{17}$-*b*-PHPMA$_{168}$ with THF (3 × 5 mL) and then ice-cold diethyl ether (3 × 5 mL). The remaining solids were dissolved in MeOH (5 mL) and precipitated from MeOH into THF three times, before being washed with ice-cold diethyl ether (3 × 5 mL) and dried. Removal of PFTMC homopolymer was assessed and confirmed via MALDI-TOF MS analysis (PFTMC-*b*-PHPMA was not observable via this technique, but PFTMC homopolymer was when present). This yielded PFTMC$_{17}$-*b*-PHPMA$_{439}$ as an off-white powder (61.9 mg, 22 %, DP$_n$ change due to fractionation). *Notes:* The dissolution of PFTMC-*b*-PHPMA can take some time and may be aided by trituration, sonication or heating; this is believed to be due to polymer self-assembly. Dissolving PFTMC-*b*-PHPMA from a dry solid is challenging and should be avoided when performing precipitations. If a turbid solution is obtained, the solid material may be collected via centrifugation. Whilst the terminal trithiocarbonate group is observable via ^{1}H-NMR (CH_2 $\delta = 3.38$ ppm, CH_3 $\delta = 1.09$ ppm), overlap with water ($\delta = 3.33$) and the PHPMA backbone methyl group ($\delta = 1.28 – 0.36$) prevents quantitative analysis of end-

group retention. **^{1}H-NMR** (500 MHz, DMSO-d_6) δ 7.81 (34H, m, Hc), 7.47 – 7.30 (93H, m, Hd & Hf), 7.18 (416H, br s, He & Hm), 4.70 (439H, br s, Hp), 4.26 (62H, s, Hb), 3.85 – 3.51 (433H, m, Ho), 3.41 – 3.34 (2H, m, Hr), 2.91 (821H, br s, Hn), 2.17 – 1.38 (524H, m, Hk), 1.28 – 0.36 (2552H, m, Hl, Hq & Hs); **^{13}C-NMR** (126 MHz, DMSO-d_6) δ 176.9 (Caa), 153.7 (Cw), 143.3 (Ct), 140.3 (Cu), 128.6 (Cd), 127.4 (Ce), 124.4 (Cf), 120.5 (Cc), 68.7 (Cb), 64.8 (Co), 54.4, 53.3, 47.6 (Cn), 45.0 (Ck), 44.6 (Ck), 21.5 (Cq), 17.9 (Cl), 16.6 (Cl); **DOSY-NMR** (500 MHz, DMSO-d_6) $D = 7.09 \times 10^{-6}$ cm^2/seconds; **GPC** (NH_4BF_4/DMF, PS standard): M_n = 5,700 g/mol, $Đ_M$ = 1.83.

PFTMC$_{16}$-*b*-PHPMA$_{73}$

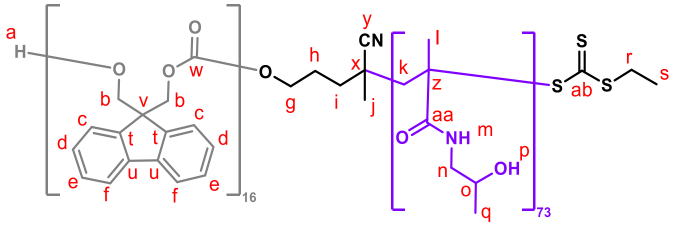

To a solution of DMA (33.4 mg, 0.162 mmol, 3.2 eq), HPMA (1,072 µL, 7.50 mmol, 150 eq), and **PFTMC$_{16}$-CTA** (252 mg, 0.050 mmol, 1.0 eq)[2] in dioxane (stabilizer-free, 10 mL), a solution of ZnTPP (475 µL of a 1 mg/mL solution in dioxane, 0.7 µmol, 0.014 eq) was added. The resulting mixture was stirred in a batch photoreactor prepared according to the designs of Corrigan et. al.[4] under red LED light irradiation. After 24 h, the reaction mixture has solidified into a light pink gel. To the resulting gel, MeOH was added until it dissolved into a light pink solution (10 mL). The crude reaction mixture was precipitated into ice-cold diethyl ether, at which point it was noted that the supernatant remained pink (indicating the presence of ZnTPP), the resulting solid was dried and redissolved in a MeOH/EtOH solution (1:1 *v*/*v*, 100mL), and precipitated twice more into ice-cold diethyl ether, before the resulting solid was dried *in vacuo* to yield PFTMC$_{16}$-*b*-PHPMA$_{160}$ as an off-white powder (1.11 g, 84 %).

Residual PFTMC homopolymer was removed by precipitation from MeOH into a THF/MeCN solution (1:1 *v*/*v*) twice, before being washed with ice-cold diethyl ether (3 × 20 mL) and dried. Removal of PFTMC homopolymer was assessed and confirmed via MALDI-TOF MS analysis (PFTMC-*b*-PHPMA was not observable via this technique, but PFTMC homopolymer was when present). This yielded PFTMC$_{16}$-*b*-PHPMA$_{73}$ as an off-white powder (885 mg, 80 %, DP$_n$ change due to fractionation). *Notes:* The dissolution of PFTMC-*b*-PHPMA can take some

time and may be aided by trituration, sonication or heating; this is believed to be due to polymer self-assembly. Dissolving PFTMC-*b*-PHPMA from a dry solid is challenging and should be avoided when performing precipitations. If a turbid solution is obtained, the solid material may be collected via centrifugation. Whilst the terminal trithiocarbonate group is observable via ^{1}H-NMR (CH_2 δ = 3.38 ppm, CH_3 δ = 1.09 ppm), overlap with water (δ = 3.33) and the PHPMA backbone methyl group (δ = 1.28 – 0.36) prevents quantitative analysis of end-group retention. **^{1}H-NMR** (500 MHz, DMSO-*d*$_6$) δ 7.80 (33H, br s, Hc), 7.36 (72H, br s, Hd & Hf), 7.19 (158H, br s, He & Hm), 4.69 (146H, br s, Hp), 4.26 (62H, br s, Hb), 3.68 (184H, br s, Ho), 3.41 – 3.34 (2H, m, Hr), 2.91 (285H, br s, Hn), 1.59 (281H, br s, Hk), 1.31 – 0.30 (890H, m, Hl, Hq & Hs); **DOSY-NMR** (500 MHz, DMSO-*d*$_6$) $D = 4.59 \times 10^{-7}$ cm^2/seconds; **GPC** (NH_4BF_4/DMF, PS standard): M_n = 1,900 g/mol, $Đ_M$ = 2.81.

PFTMC$_{16}$-*b*-(PHPMA$_{95}$-*co*-PFlMA$_5$)

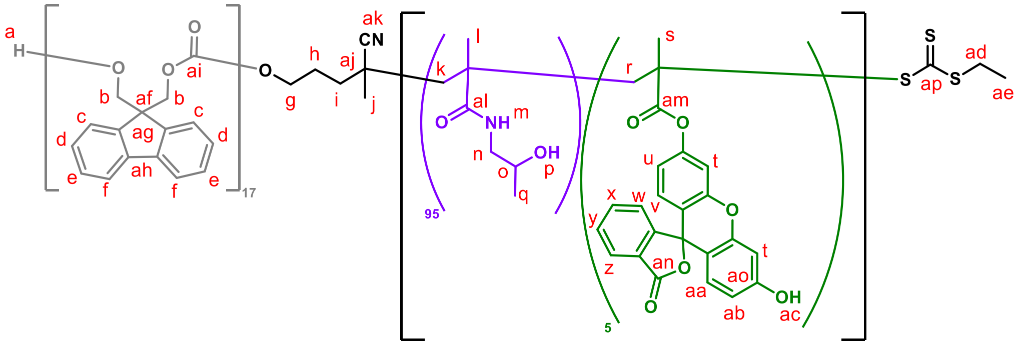

To a solution of DMA (3.3 mg, 0.016 mmol, 3.2 eq), HPMA (107 µL, 0.750 mmol, 150 eq), fluorescein O-methacrylate (FlMA, 10.0 mg, 0.025 mmol, 5 eq) and **PFTMC$_{16}$-CTA** (25.2 mg, 0.005 mmol, 1.0 eq)[2] in dioxane (stabilizer-free, 2 mL), a solution of ZnTPP (70 µL of a 1 mg/mL solution in dioxane, 0.07 µmol, 0.014 eq) was added. The resulting mixture was stirred in a batch photoreactor prepared according to the designs of Corrigan et. al.[4] under red LED light irradiation. After 20 h, the reaction mixture has formed a light pink gum. To the crude reaction mixture, MeOH was added until it dissolved into a light pink solution (5 mL) before being precipitated into ice-cold diethyl ether, at which point it was noted that the supernatant remained pink (indicating the presence of ZnTPP), the resulting fine white powder was dried and redissolved in MeOH (10mL), and precipitated twice more from MeOH into ice-cold diethyl ether, before being precipitated three times from MeOH into a THF/MeCN solution (1:1 *v*/*v*). The resulting fine white powder was dried *in vacuo* to yield PFTMC$_{16}$-*b*-(PHPMA$_{95}$-*co*-PFlMA$_5$) as an off-white powder (42 mg, 28 %).

Notes: The dissolution of PFTMC-*b*-PHPMA can take some time and may be aided by trituration, sonication or heating; this is believed to be due to polymer self-assembly. Dissolving PFTMC-*b*-PHPMA from a dry solid is challenging and should be avoided when performing precipitations. If a turbid solution is obtained, the solid material may be collected via centrifugation. Whilst the terminal trithiocarbonate group is observable via ^{1}H-NMR (CH_2 δ = 3.38 ppm, CH_3 δ = 1.09 ppm), overlap with water (δ = 3.33) and the PHPMA backbone methyl group (δ = 1.28 – 0.36) prevents quantitative analysis of end-group retention. **^{1}H-NMR** (500 MHz, DMSO-d_6) δ 10.21 (5H, br s, Hac), 8.02 (6H, br s, Hz), 7.90 – 7.69 (44H, m, Hc, Hy & Hx), 7.62 – 6.97 (223H, m, Hd, He, Hf, Hm & Hw), 6.86 (9H, br s, Ht), 6.75 (5H, br s, Hu), 6.61 (10H, br s, Hv & Haa), 4.70 (95H, br s, Hp), 4.26 (65H, br s, Hb), 3.67 (93H, br s, Ho), 3.41 – 3.34 (2H, m, Had), 2.91 (187H, br s, Hn), 1.64 (253H, br s, Hk), 1.29 – 0.29 (636H, m, Hl, Hq & Hae); **^{13}C-NMR** (126 MHz, DMSO-d_6) δ 177.0 (Cal), 168.6 (Can), 159.7 (Cao), 153.7 (Cai), 145.4, 143.3 (Cag), 140.3 (Cah), 128.6 (Cd), 127.4 (Ce), 124.4 (Cf), 120.5 (Cc), 68.7 (Cb), 64.8 (Co), 53.3, 47.6 (Cn), 45.0 (Ck & Cr), 44.6 (Ck & Cr), 39.5 (Ck & Cr), 21.5 (Cq), 17.9 (Cl & Cs), 16.6 (Cl & Cs), 15.2 (Cl & Cs); **DOSY-NMR** (500 MHz, DMSO-d_6) $D = 3.13 \times 10^{-7}$ cm^2/seconds; **GPC** (NH_4BF_4/DMF, PS standard): M_n = 1,600 g/mol, $Đ_M$ = 6.58.

Self-assembly procedures

General procedure for the CDSA of PFTMC-*b*-PHPMA

Unimer solutions (either 20 mg/mL or 200 mg/mL) of the appropriate block copolymer were prepared by dissolution in common solvent (DMSO). An aliquot of this solution was then further diluted in an amount of common solvent appropriate to the final concentration of polymer and desired solvent composition. To this solution, selective solvent (EtOH) was added slowly, and the vial was sealed, manually shaken for 10 s, and then vortexed mixed for a further 10 s.

For samples annealed at 70 °C: Where indicated, the sample was then annealed at 70 °C for the indicated time and allowed to cool in the heating block until it reached room temperature (23 °C), before being further aged at 23 °C until the total ageing time was 24 h.

For samples aged at room temperature: Where indicated, the sample was then aged at 23 °C for 24 h.

Example procedure for the self-assembly of $PFTMC_{17}$-*b*-$PHPMA_{439}$

To a unimer solution of $PFTMC_{17}$-*b*-$PHPMA_{439}$ (50 µL, 200 mg/mL in DMSO), MeOH was slowly added (950 µL), and the sample was manually shaken for 10 s, then vortex mixed for 10 s, and annealing at 70 °C for 3 h before cooling to 23 °C and aged at 23 °C for 21 h. The resulting nanofibers were then imaged via TEM.

General Procedure for the preparation of seed nanofibers from disperse nanofibers

Disperse nanofibers (ranging from 1 mg/mL to 10 mg/mL) were sonicated for at least 3 h using either a Hielschur UP100H sonication probe, or a a Fisherbrand 112xx series advanced ultrasonic cleaner (FB-11203) (37 MHz at 80 % power). The temperature was kept between 0 °C and 23 °C using an ice bath. The resulting seed nanofibers were then imaged via TEM. *Note*: If the lab has recirculating chilled water condensers, the sample may be immersed into a larger water bath equipped with a stirrer bar and a submersed condenser, and used in place of the ice bath. This provides consistent temperatures (ca. 18 °C from our experience), negating the need to continually check the levels of ice in the ice bath.

Example procedure for the preparation of seed nanofibers from disperse nanofibers

Disperse $PFTMC_{17}$-*b*-$PHPMA_{439}$ nanofibers (1 mL, 10 mg/mL) were sonicated for 4 h using a Hielschur UP100H sonication probe, with the temperature kept between 0 °C and 23 °C using an ice bath. The resulting seed nanofibers were then imaged via TEM. It was observed that the solution of nanofibers became noticeably less viscous and more transparent after sonication.

General Procedure for the one-step preparation of seed nanofibers

PFTMC-*b*-PHPMA Unimer solution (either 20 mg/mL or 200 mg/mL) was prepared by dissolution in common solvent (DMSO). An aliquot of this solution was then further diluted in an amount of common solvent appropriate to the final concentration of polymer and solvent composition. To this solution, selective solvent (EtOH) was added slowly, and the vial was sealed, manually shaken for 10 s and then vortexed mixed for a further 10 s. This solution was sonicated immediately for at least 3 h using a Hielschur UP100H sonication probe. The temperature was kept between 0 °C and 23 °C using an ice bath. After sonication, the solution was aged at 23 °C for 21 h. The resulting seed nanofibers were then imaged via TEM.

Example Procedure for the one-step preparation of seed nanofibers

To a unimer solution of $PFTMC_{17}$-*b*-$PHPMA_{439}$ (50 µL, 200 mg/mL, DMSO), MeOH was then slowly added (950 µL), and the sample was manually shaken for 10 s, then vortex mixed for 10 s, and sonicated immediately for 4 h using a Hielschur UP100H sonication probe, with the temperature kept between 0 °C and 23 °C using an ice bath. The solution was then aged at 23 °C for 21 h. The resulting seed nanofibers were then imaged via TEM.

General procedure for the preparation of low dispersity nanofibers from seed nanofibers using the seeded-growth method (living CDSA)

A solution of seed nanofibers (between 0.1 mg/mL and 1 mg/mL) were diluted in a volume of selective solvent appropriate to the final concentration of polymer and solvent composition. To this solution, an aliquot of unimer solution (20 mg/mL, DMSO) appropriate to the desired m_{unimer}:m_{seed} ratio was added, the sample was manually shaken for 10 s, then vortex mixed for 10 s, and aged at 23 °C for 24 h. The resulting low dispersity nanofibers were then imaged via TEM. Samples with an m_{unimer}:m_{seed} ratio of above 10 were added iteratively in aliquots of no more than 10 followed by ageing for 24h between the addition of each aliquot.

Example procedure for the preparation of low dispersity nanofibers from seed nanofibers

To a solution of 30 nm ± 8 nm **seeds2** ($Đ_L$ = 1.07, 500 µL, 10 mg/mL), EtOH (1000 µL) was added. To this solution, $PFTMC_{17}$-*b*-$PHPMA_{439}$ unimer solution (100 µL, 100 mg/mL, m_{unimer}:m_{seed} = 2) was added, and the sample was manually shaken for 10 s, then vortex mixed for 10 s, and aged at 23 °C for 24 h. The resulting low dispersity 70 ± 14 nm **nanofibers2** were characterized via TEM (Figure S15, $Đ_L$ = 1.04).

General procedure for the transfer of low dispersity nanofibers into water

Low length-dispersity nanofibers (500 µL – 2 mL, 2 – 10 mg/mL) were manually shaken for 10 s, then vortex mixed for 10 s, before being placed inside a dialysis membrane (Sigma Aldrich, MWCO = 12,000 – 14,000 Da), sealed with clips (Spectrum Chemical), and dialyzed into deionized water (500 mL) for 24 h with a minimum of three dialysate changes. The dialysis membrane was opened, and the nanofiber solution was transferred to a vial. The solution was

weighed, and filtered deionized water was added to make the sample up to 1 mg/mL gravimetrically. The resulting low dispersity nanofibers were characterized via TEM.

Example procedure for the transfer of low dispersity nanofibers into water

70 ± 14 nm **nanofibers2** ($Đ_L$ = 1.04, 1.5 mL, 10 mg/mL) were placed inside a dialysis membrane (Sigma Aldrich, MWCO = 12,000 – 14,000 Da), sealed with clips (Spectrum Chemical), and dialyzed into deionized water (500 mL) for 24 h with a minimum of three dialysate changes. The dialysis membrane was opened, and the nanofiber solution was transferred to a vial. The solution was weighed, and filtered, deionized water was added to make the sample up to 1 mg/mL gravimetrically (2 g). The resulting low dispersity 70 ± 14 nm **nanofibers2** were characterized via TEM ($Đ_L$ = 1.04).

General procedure for the preparation of segmented nanofibers

A solution of seed nanofibers or low dispersity nanofibers (between 0.1 and 5 mg/mL) were diluted in a volume of selective solvent appropriate to the final concentration of polymer and solvent composition. To this solution, an aliquot of a different unimer solution in common solvent (DMSO, 20 mg/mL) appropriate to the desired m_{unimer}:m_{seed} ratio was added, the sample was manually shaken for 10 s, then vortex mixed for 10 s, and aged at 23°C for 24 h. The resulting segmented low dispersity nanofibers were then imaged via TEM.

Procedure for the preparation of segmented nanofibers 1

30 nm ± 8 nm $PFTMC_{17}$-*b*-$PHPMA_{439}$ nanofiber **seeds2** ($Đ_L$ = 1.07, 100 µL, 10 mg/mL) were diluted in MeOH (900 µL). To this solution, $PFTMC_{16}$-*b*-($PHPMA_{95}$-*co*-$PFlMA_5$) unimer solution (50 µL, 20 mg/mL in DMSO, m_{unimer}:m_{seed} = 1) was added, and the sample was manually shaken for 10 s, then vortex mixed for 10 s, and aged at 23 °C in the dark for 24 h. The resulting triblock segmented nanofibers were placed inside a dialysis membrane (Sigma Aldrich, MWCO = 12,000 – 14,000 Da), sealed with clips (Spectrum Chemical), and dialyzed into deionized water (500 mL) in the dark for 24 h with a minimum of three dialysate changes. The dialysis membrane was opened, and the nanofiber solution was transferred to a vial. The solution was weighed, and filtered deionized water was added to make the sample up to 1 mg/mL gravimetrically (2 g). The resulting low dispersity nanofibers were characterized via TEM ($Đ_L$ = 1.52, W_n = 15 nm, $Đ_W$ = 1.36), DLS and ζ-potential and stored in the dark.

General procedure for the preparation of nanospheres via dialysis

Unimer solutions (20 mg/mL) were prepared by dissolution in common solvent (DMSO). Aliquots of these unimer solutions appropriate to the desired composition and concentration of the resulting nanospheres were combined in a vial, and further diluted in an amount of common solvent appropriate to the final concentration of polymer. The sample was manually shaken for 10 s, then vortex mixed for 10 s. This solution (500 µL – 5 mL, 2 – 10 mg/mL) was then placed inside a dialysis membrane (Sigma Aldrich, MWCO = 12,000 – 14,000 Da), sealed with clips (Spectrum Chemical), and dialyzed into deionized water (500 mL) for 24 h in the dark with a minimum of three dialysate changes. The dialysis membrane was opened, and the nanosphere solution was transferred to a vial. The solution was weighed, and filtered deionized water was added to make the sample up to 1 mg/mL gravimetrically. The resulting nanospheres were characterized via TEM, DLS, and ζ-potential and stored in the dark.

Example procedure for the preparation of blend nanospheres via dialysis

An aliquot of $PFTMC_{17}$-*b*-$PHPMA_{439}$ unimer solution (25 µL, 20 mg/mL in DMSO) and $PFTMC_{16}$-*b*-($PHPMA_{95}$-*co*-$PFlMA_{5}$) unimer solution (25 µL, 20 mg/mL in DMSO) was diluted in water (950 µL). The sample was manually shaken for 10 s, then vortex mixed for 10 s, before being placed inside a dialysis membrane (Sigma Aldrich, MWCO = 12,000 – 14,000 Da), sealed with clips (Spectrum Chemical), and dialyzed into deionized water (500 mL) in the dark for 24 h with a minimum of three dialysate changes. The dialysis membrane was opened, and the nanosphere solution was transferred to a vial. The solution was weighed, and filtered deionized water was added to make the sample up to 1 mg/mL gravimetrically (1 g). The resulting **blended nanospheres 1** were characterized via TEM (D_n = 16 ± 4 nm, $Đ_D$ = 1.06) and stored in the dark. N.B. It was observed that the colour and fluorescence emission of $PFTMC_{16}$-*b*-($PHPMA_{95}$-*co*-$PFlMA_{5}$) samples changes depending on the solvent system. In DMSO, $PFTMC_{16}$-*b*-($PHPMA_{95}$-*co*-$PFlMA_{5}$) unimer is colourless with a blue fluorescence upon UV irradiation (TLC box, λ = 368 nm). Upon transfer into water, the solution turns bright green, and a bright green emission is also observed upon UV irradiation.

Characterization data for individual samples used in this work

PFTMC$_{17}$-*b*-PHPMA$_{439}$ nanofiber seeds 1: TEM (DMSO/EtOH, 5:95 *v/v*, 1 mg/mL, 0.1 wt%): Figure3B, L_n = 27 nm, $Đ_L$ = 1.19, σ_L = 12 nm.

PFTMC$_{17}$-*b*-PHPMA$_{439}$ nanofiber seeds 2: TEM (DMSO/EtOH, 5:95 *v/v*, 10 mg/mL, 1 wt%, two-step procedure): Figure3B, L_n = 30 nm, $Đ_L$ = 1.07, σ_L = 8 nm.

PFTMC$_{17}$-*b*-PHPMA$_{439}$ nanofiber seeds 3: TEM (DMSO/EtOH, 5:95 *v/v*, 10 mg/mL, 1 wt%, one-step procedure): Figure3C, L_n = 25 nm, $Đ_L$ = 1.11, σ_L = 9 nm.

PFTMC$_{17}$-*b*-PHPMA$_{439}$ nanofibers1: TEM: FigureS15, u/s = 2, L_n = 103 nm, $Đ_L$ = 1.04, σ_L = 20 nm; AFM: mean height of 8.2 ± 0.5 nm.

PFTMC$_{17}$-*b*-PHPMA$_{439}$ nanofibers2: TEM (DMSO/EtOH, 5:95 *v/v*): FigureS16, L_n =70 nm, $Đ_L$ = 1.04, σ_L = 14 nm; (H_2O): Figure3D, L_n =70 nm, $Đ_L$ = 1.04, σ_L = 14 nm.

PFTMC$_{16}$-*b*-(PHPMA$_{95}$-*co*-PFlMA$_5$)]-*m*-[PFTMC$_{17}$-*b*-PHPMA$_{439}$]-*m*-[PFTMC$_{16}$-*b*-(PHPMA$_{95}$-*co*-PFlMA$_5$)] triblock segmented nanofibers 1: TEM (H_2O): Figure5E, L_n = 55 nm, $Đ_L$ = 1.52, σ_L = 40 nm, W_n = 15 nm, $Đ_W$ = 1.36; DLS: D_h = 208 ± 5 nm in 5 mM NaCl, 0.1 mg/mL; and *app.* ζ-potential = -9.3 ± 0.4 mV.

PFTMC$_{16}$-*b*-(PHPMA$_{95}$-*co*-PFlMA$_5$)]-blend-[PFTMC$_{17}$-*b*-PHPMA$_{439}$] blended nanospheres 1: TEM (H_2O): Figure5F, D_n = 16 nm, $Đ_D$ = 1.06, σ_D = 4 nm; DLS: D_h = 179 ± 13 nm in 5 mM NaCl, 0.1 mg/mL; and *app.* ζ-potential = -6.5 ± 0.8 mV.

Cell culture protocols

HeLa (human female cervical carcinoma cells) were grown in DMEM media with high glucose (4.5 g/L), WI-38 (caucasian female fibroblast-like foetal lung cells) were grown in MEM media, and U-87 MG (human male epithelial glioblastoma cells) were grown in DMEM media with high glucose (4.5 g/L). All cells were cultured in a humidified 5% CO_2 incubator at 37°C. All growth media were supplemented with 10% fetal bovine serum (FBS). Confluent cultures (80% or less) were detached from the surface using trypsin (TrypLE Express™) and plated at 5×10^3 - 1×10^4 cells/well in 96-well plates for cytotoxicity studies.

Cell Viability Assays

The influence of PFTMC-*b*-PHPMA nanofibers and nanospheres on WI-38 and HeLa cells was evaluated after 72 h of exposure and analyzed with a dual calcein / alamarBlue® assay (Figure 4 and Table S5-S8). Cell survival was quantified by measuring calcein AM fluorescence. The fluorescence, retained within live cells only, results from activity of esterases on the (nonfluorescent) calcein AM (Molecular Probes). Changes in cell metabolism were assessed using alamarBlue™ (AB, Life Technologies), a cytosolic substrate for reductive metabolism (resazurin to resorufin) whose fluorescence spectrum changes on reduction by cytosolic enzymes. WI-38 and HeLa cells were incubated with 0-100 µg/mL of sample for 72 h. Each experiment was repeated at least in duplicate in medium with reduced FBS (5%), and each data point was conducted in sextuplicate. Staurosporine (1 µM/mL, Enzo Life Sciences) was used as a positive control. After 72 h, the plates were washed with PBS, and alamarBlue® (5 % solution) and calcein AM (3 µM) were added in medium without FBS. After 1 h incubation, the fluorescence of both dyes was read using a plate reader (BMG Labtech CLARIOstar) (AB λ_{ex} = 545 nm, λ_{em} = 590 nm, calcein λ_{ex} = 494 nm, λ_{em} = 517 nm). Results were expressed as the % of viable cells compared to the control cell population.

Cell Uptake Assays

Cell uptake assays were conducted using either CLSM or flow cytometry. U-87 MG cells were cultured in well plates and incubated with polymer nanoparticles according to the procedures listed. For flow cytometry, cell viability was probed using PI, which is internalized by non-viable cells, thus labelling them as PI positive. For all flow cytometry studies, cells incubated with branched polyethylimine (500 µg/mL, Sigma Aldrich) was used as a positive control as well as a

media blank and cells both with and without PI. Flow cytometry data is presented in Figure 6C-D, S20-S21 and Table S9-S12.

Sonication Kinetics Study

As we observed evidence of nanofiber fusion, sonication kinetics studies were conducted on $PFTMC_{17}$-*b*-$PHPMA_{439}$ nanofibers in DMSO/EtOH (5:95 *v*/*v*, 1 mg/mL) after self-assembly at room temperature (23 °C) to investigate the time required to form short, seed nanofibers via fragmentation and whether any fused species persisted (Figure S12). Fragmentation proceeded quickly with an exponential decay in nanofiber length that reached 62 nm ± 14 nm ($Đ_L$ = 1.05) after 30 minutes, in accordance with prior studies on the sonication of nanofibers.[5] After 6 hours, the length further decreased to 27 nm ± 12 nm ($Đ_L$ = 1.19) yielding **seeds1**. The nanofibers also exhibited a low length-dispersity at all timepoints, with a $Đ_L$ of less than 1.1. This data was fitted to the 'one phase decay' nonlinear regression model in Graphpad Prism yielding an equation that related nanofiber length to time of: L_n=(1,144-29.59)*exp(-0.1182*t)+29.59 (where t is time, R^2 = 0.76). The $t_{½}$ of nanofiber length reduction was found to be 5.9 min. There was no evidence of fused nanofiber species at any timepoint, indicating that these structures are broken up very quickly. Thus, a sonication time of at least 2 hours is sufficient to reproducibly form ca. 30 nm low-dispersity seed nanofibers from samples that can include fused nanofiber species.

Supplementary Figures

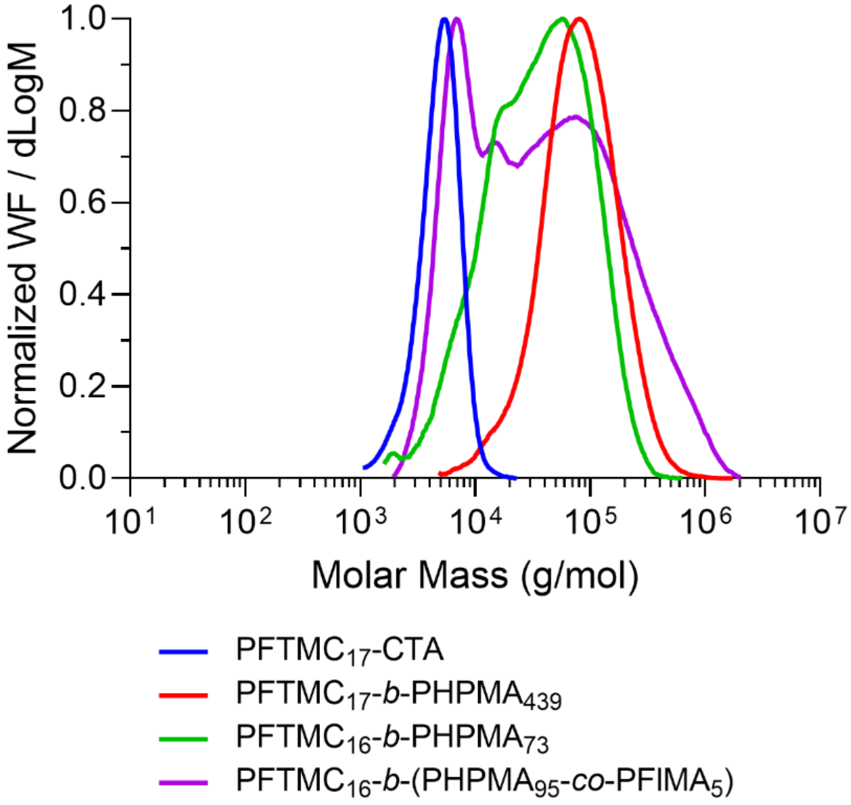

Figure S1. GPC data for $PFTMC_{17}$-CTA, $PFTMC_{17}$-*b*-$PHPMA_{439}$, $PFTMC_{16}$-*b*-$PHPMA_{73}$ and $PFTMC_{16}$-*b*-($PHPMA_{95}$-*co*-$PFlMA_5$) after purification.

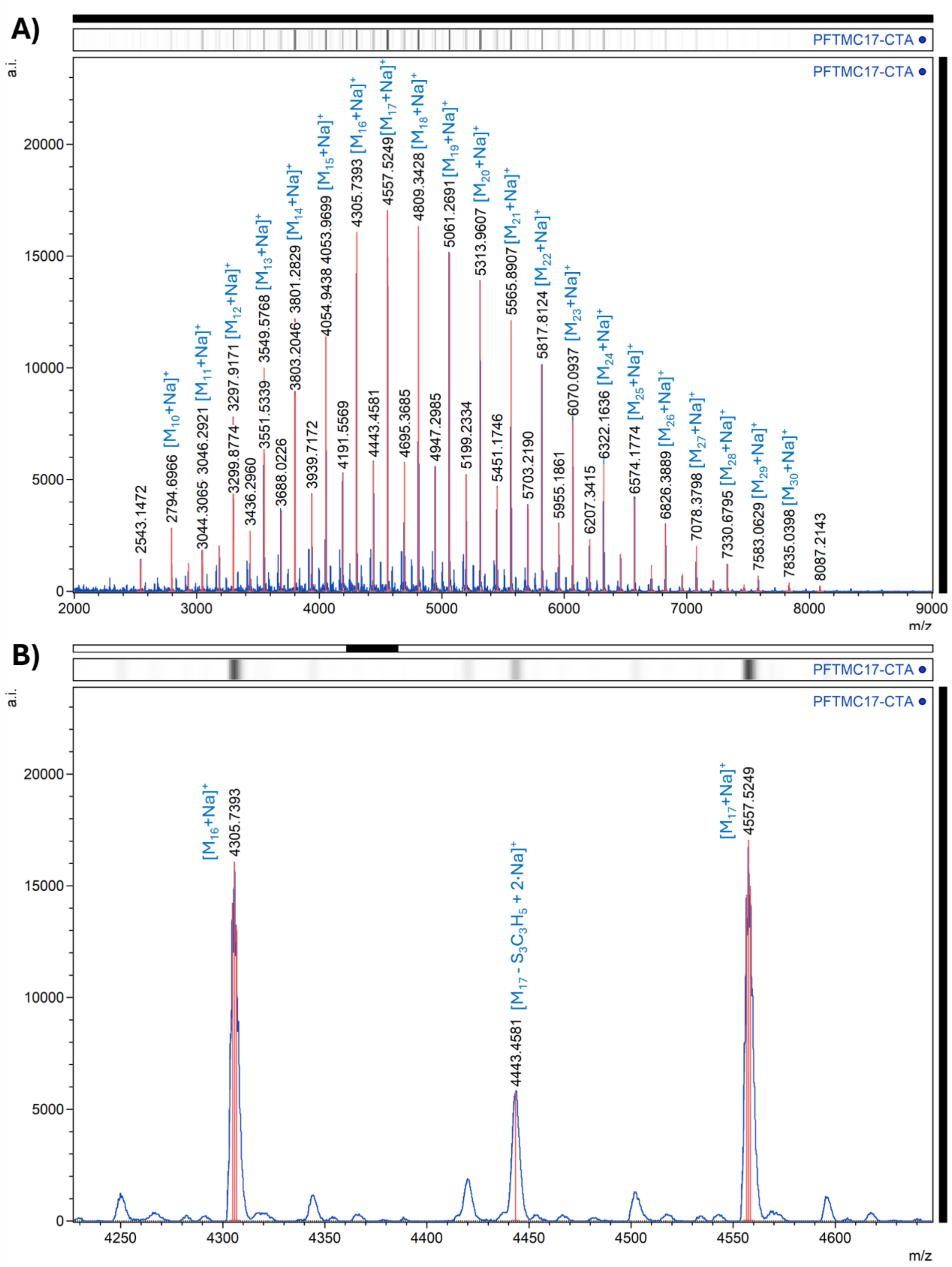

Figure S2. (A) MALDI-TOF MS spectrum of PFTMC$_{17}$-CTA with (B) magnification.

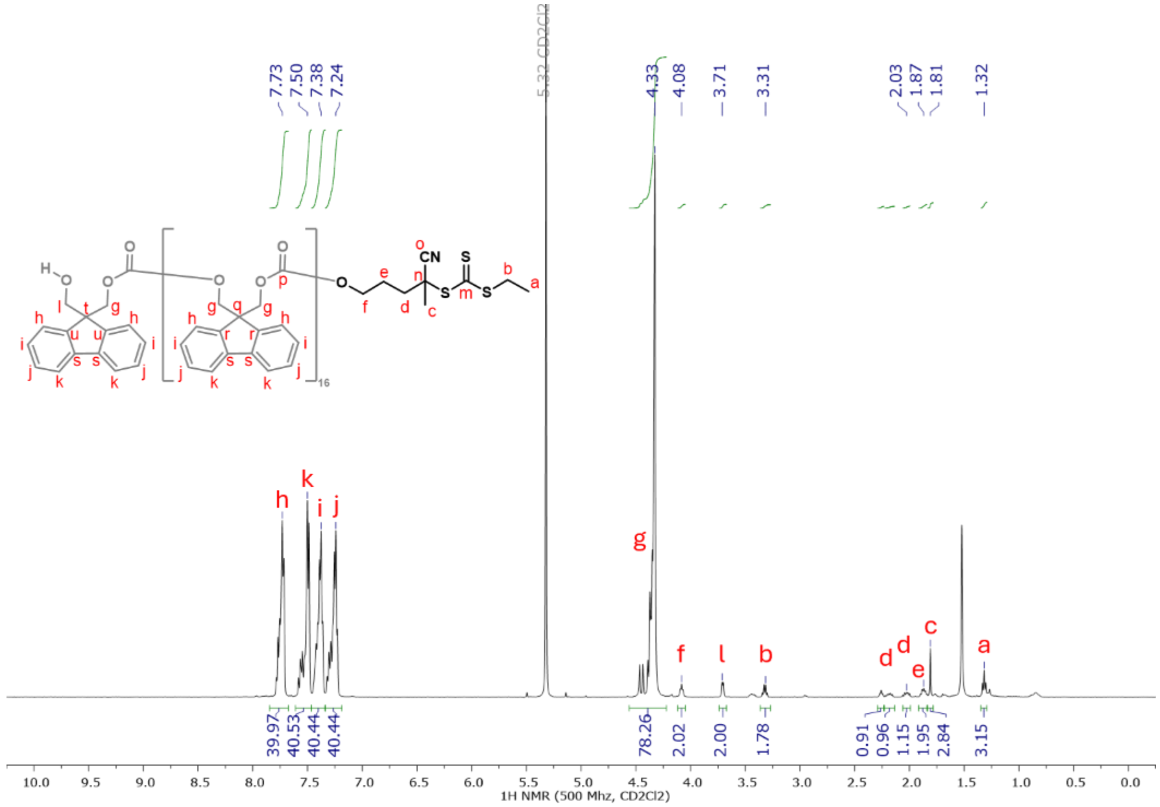
7.73
7.50
7.38
7.24
5.32 CD2Cl2
4.33
4.08
3.71
3.31
2.03
1.87
1.81
1.32
39.97
40.53
40.44
40.44
78.26
2.02
2.00
1.78
0.91
0.96
1.15
1.95
2.84
3.15
1H NMR (500 Mhz, CD2Cl2)

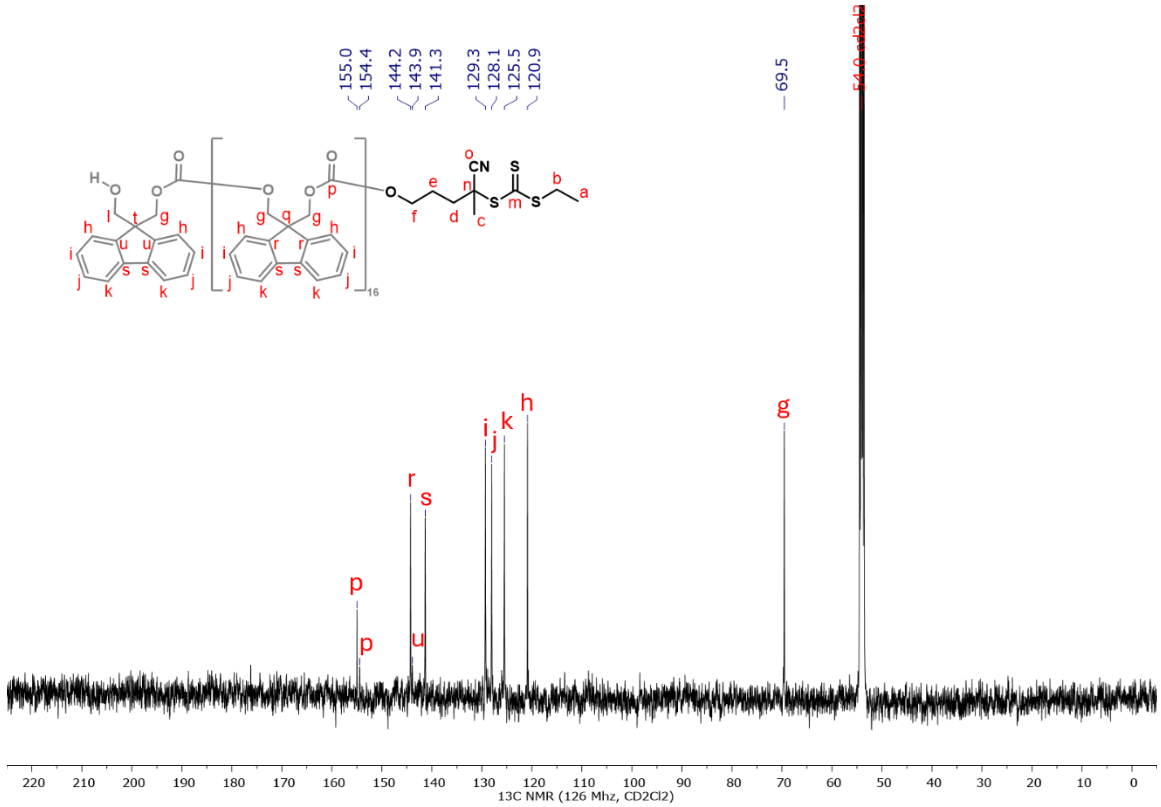
155.0
154.4
144.2
143.9
141.3
129.3
128.1
125.5
120.9
69.5
13C NMR (126 Mhz, CD2Cl2)

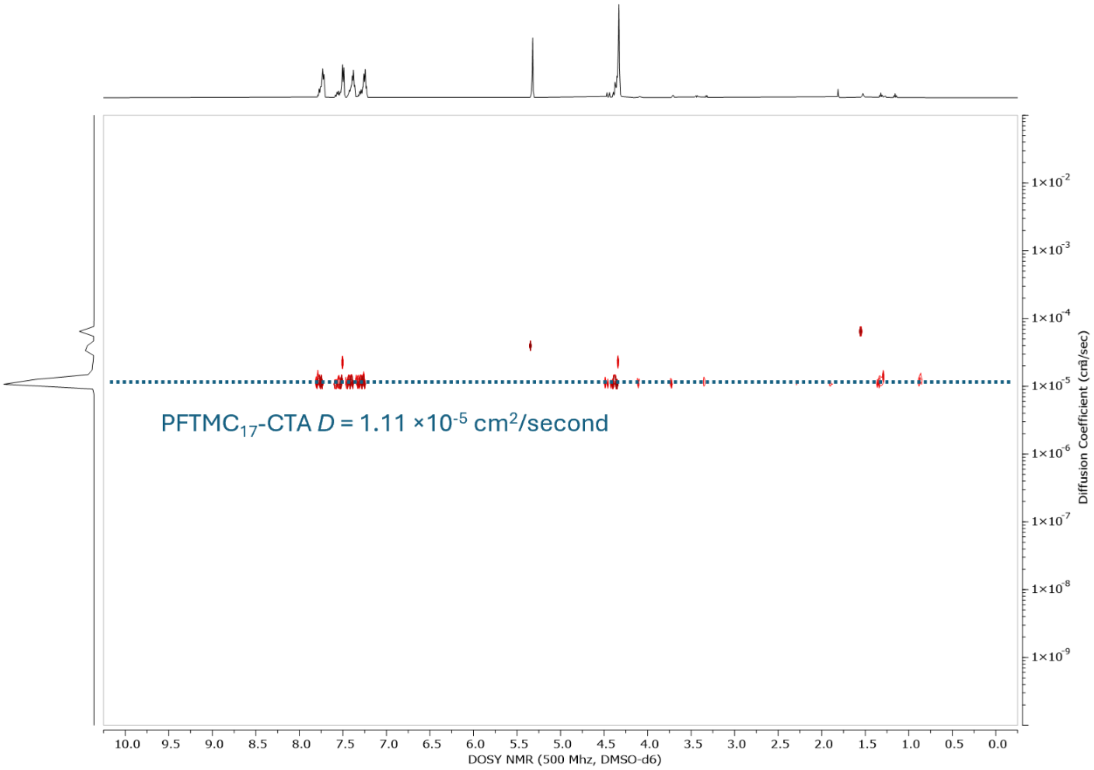

Figure S3. ^{1}H-NMR, ^{13}C-NMR and DOSY-NMR spectra of PFTMC$_{17}$-CTA.

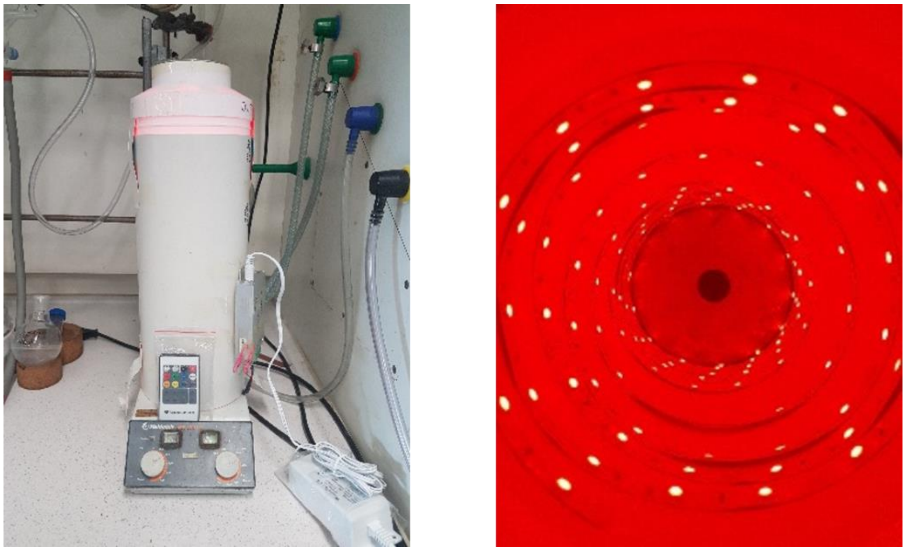

Figure S4. Custom-built visible light photoreactor for PET-RAFT polymerization.[4]

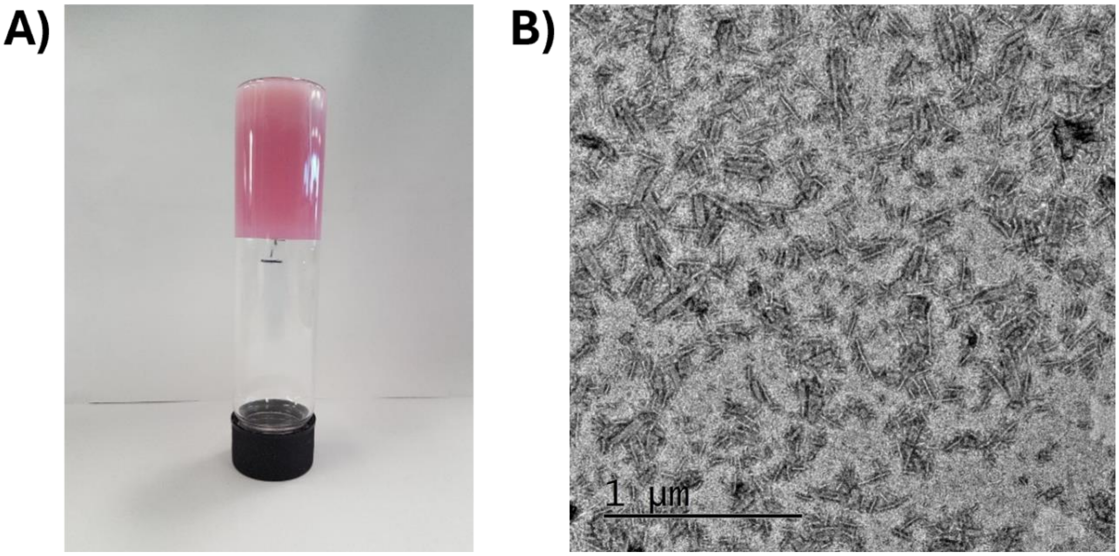

Figure S5. (A) Organogel formed after PET-RAFT polymerization of $PFTMC_{17}$-*b*-$PHPMA_{168}$ in dioxane. (B) TEM micrograph of the crude reaction mixture after dilution in MeOH. Gelation is thought to occur due to polymerization-induced crystallization-driven self-assembly (PI-CDSA). The TEM sample was stained with uranyl acetate (3 wt% in EtOH).

Table S1. Solvent compatibility of $PFTMC_{16}$-*b*-$PHPMA_{160}$ ($PFTMC_{16}$-*b*-$PHPMA_{73}$ pre-purification).

**Solvent**	**Solubility of $PFTMC_{16}$-*b*-$PHPMA_{160}$**
MeOH	Soluble (but limited)[a]
EtOH	Soluble (but limited)[a]
DMSO	Soluble[a]
DMF	Soluble[a]
Water	Soluble[a]
Dioxane	Insoluble / sparingly soluble
THF	Sparingly soluble
MeCN	Insoluble
CH2Cl2	Sparingly soluble
Et2O	Insoluble
Hexane	Insoluble

[a] It is likely that $PFTMC_{16}$-*b*-$PHPMA_{160}$ can self-assemble in these solvents.

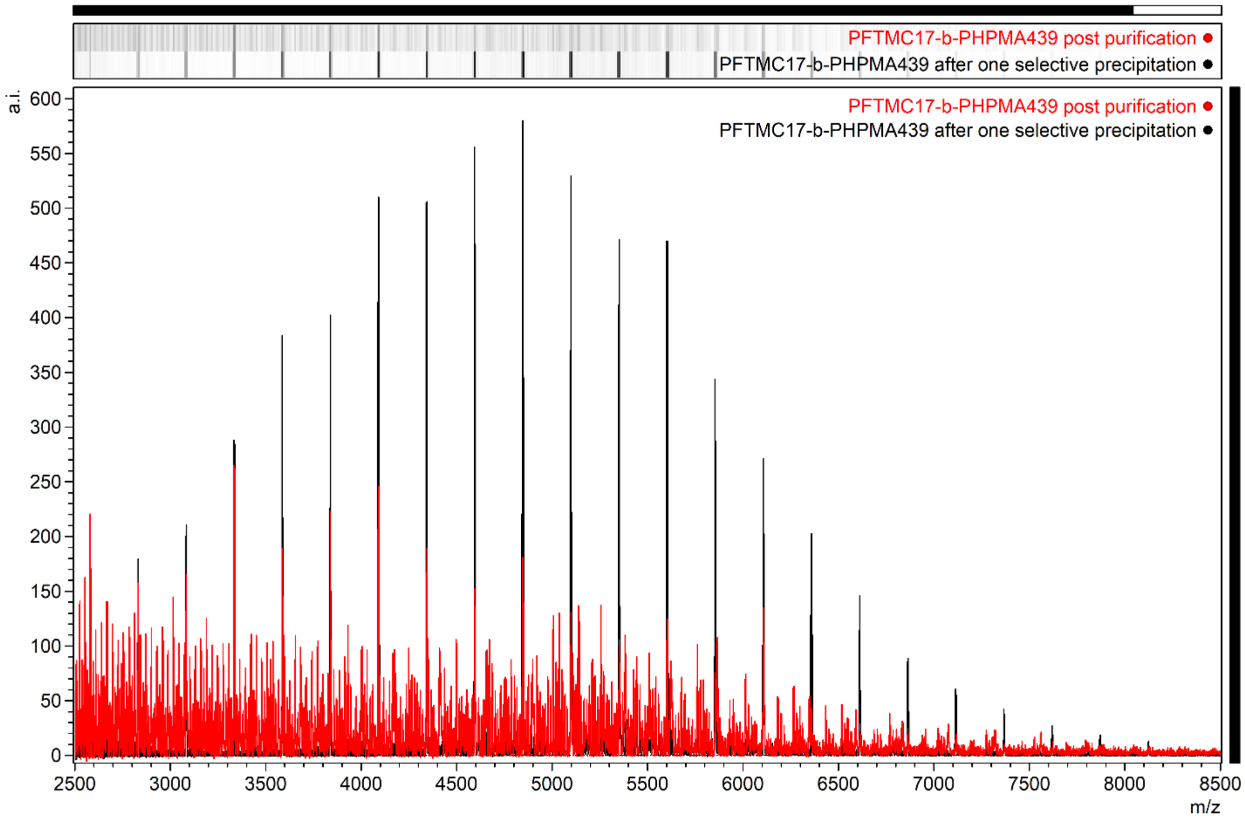

Figure S6. MALDI-TOF MS of PFTMC$_{17}$-*b*-PHPMA$_{439}$ before and after purification by precipitation from MeOH into THF.

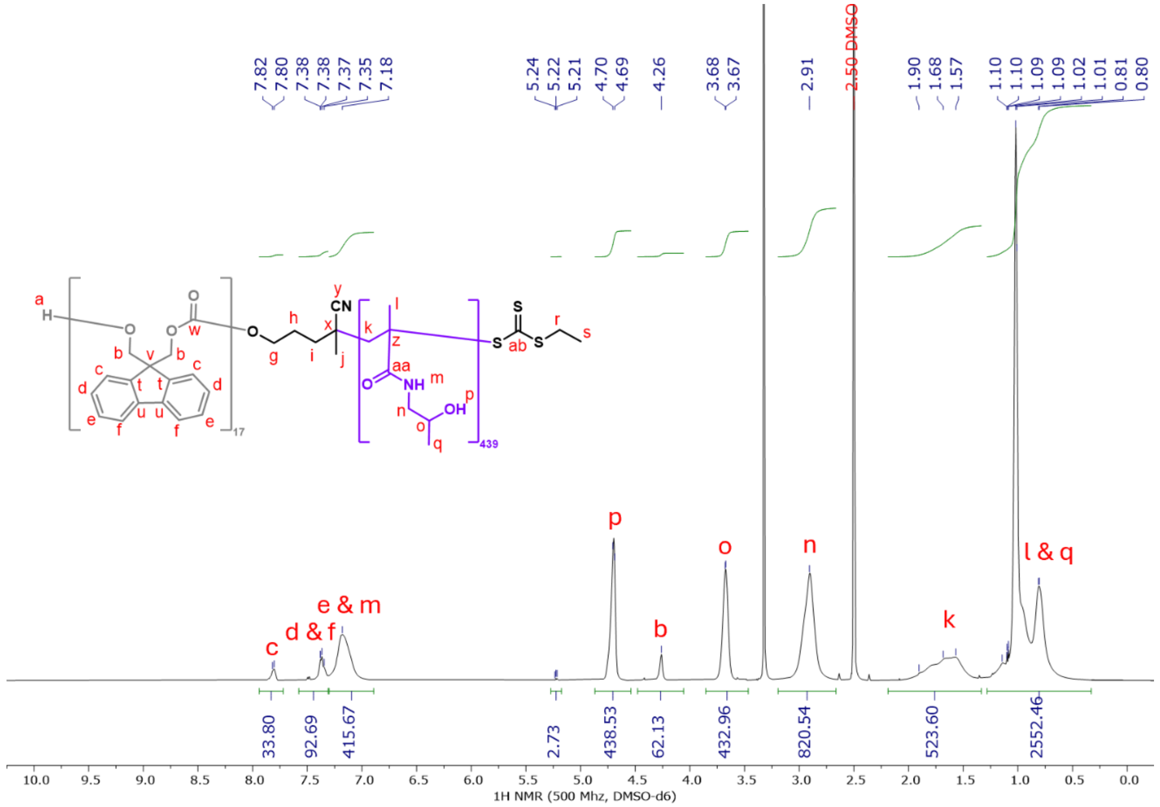
7.82
7.80
7.38
7.38
7.37
7.35
7.18
5.24
5.22
5.21
4.70
4.69
4.26
3.68
3.67
2.91
2.50 DMSO
1.90
1.68
1.57
1.10
1.10
1.09
1.09
1.02
1.01
0.81
0.80
c
d & f
e & m
p
b
o
n
k
l & q
33.80
92.69
415.67
2.73
438.53
62.13
432.96
820.54
523.60
2552.46
1H NMR (500 Mhz, DMSO-d6)

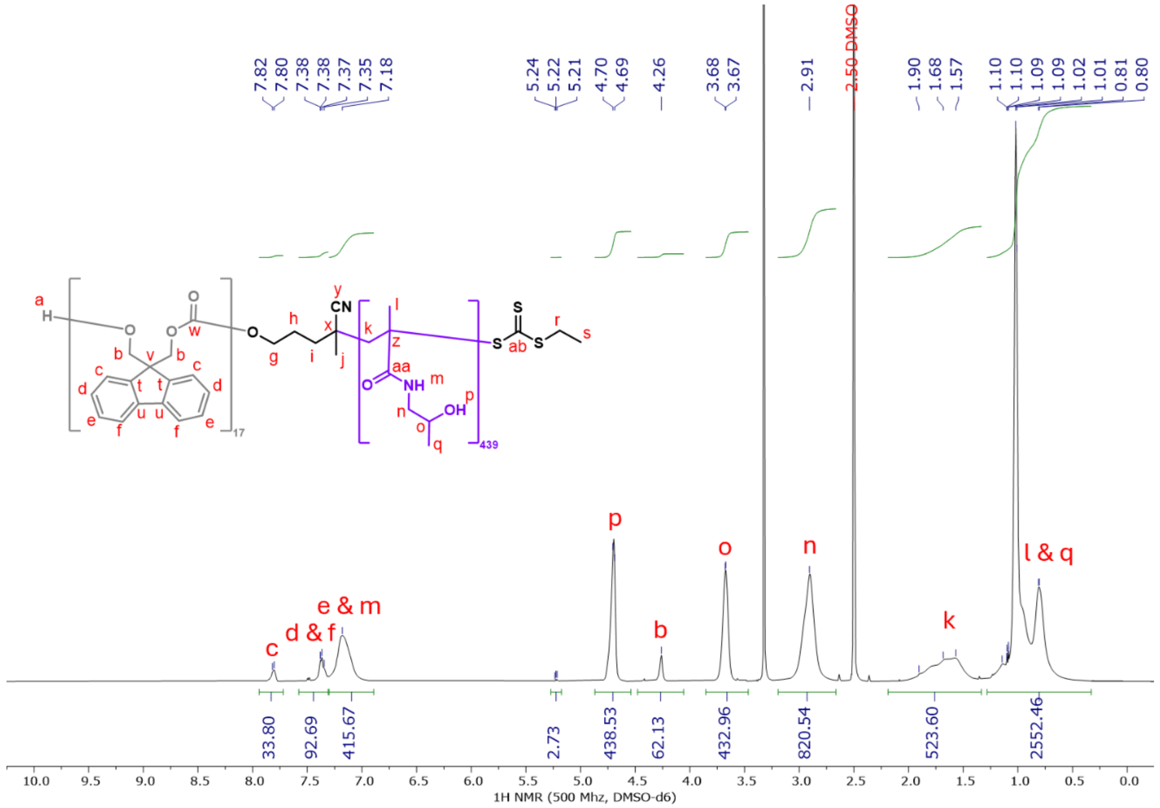
7.82
7.80
7.38
7.38
7.37
7.35
7.18
5.24
5.22
5.21
4.70
4.69
4.26
3.68
3.67
2.91
2.50 DMSO
1.90
1.68
1.57
1.10
1.10
1.09
1.09
1.02
1.01
0.81
0.80
c
d & f
e & m
p
b
o
n
k
l & q
33.80
92.69
415.67
2.73
438.53
62.13
432.96
820.54
523.60
2552.46
1H NMR (500 Mhz, DMSO-d6)

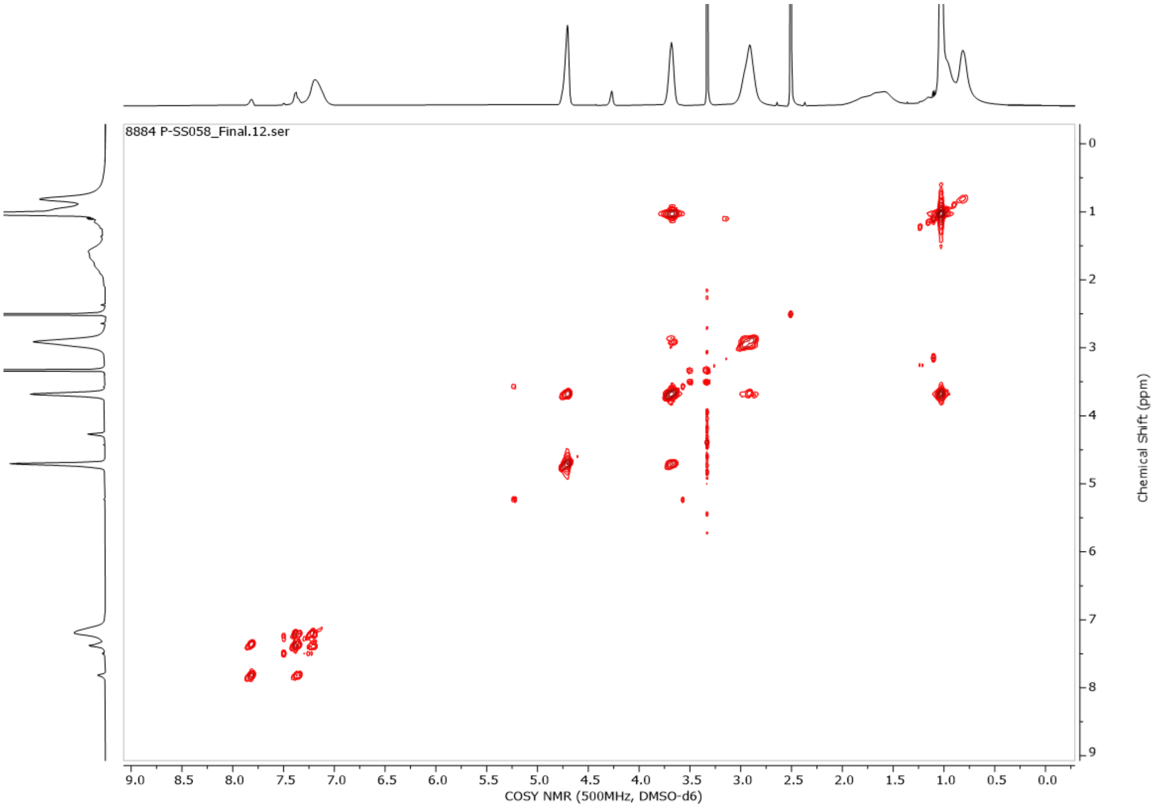
8884 P-SS058_Final.12.ser
COSY NMR (500MHz, DMSO-d6)
Chemical Shift (ppm)

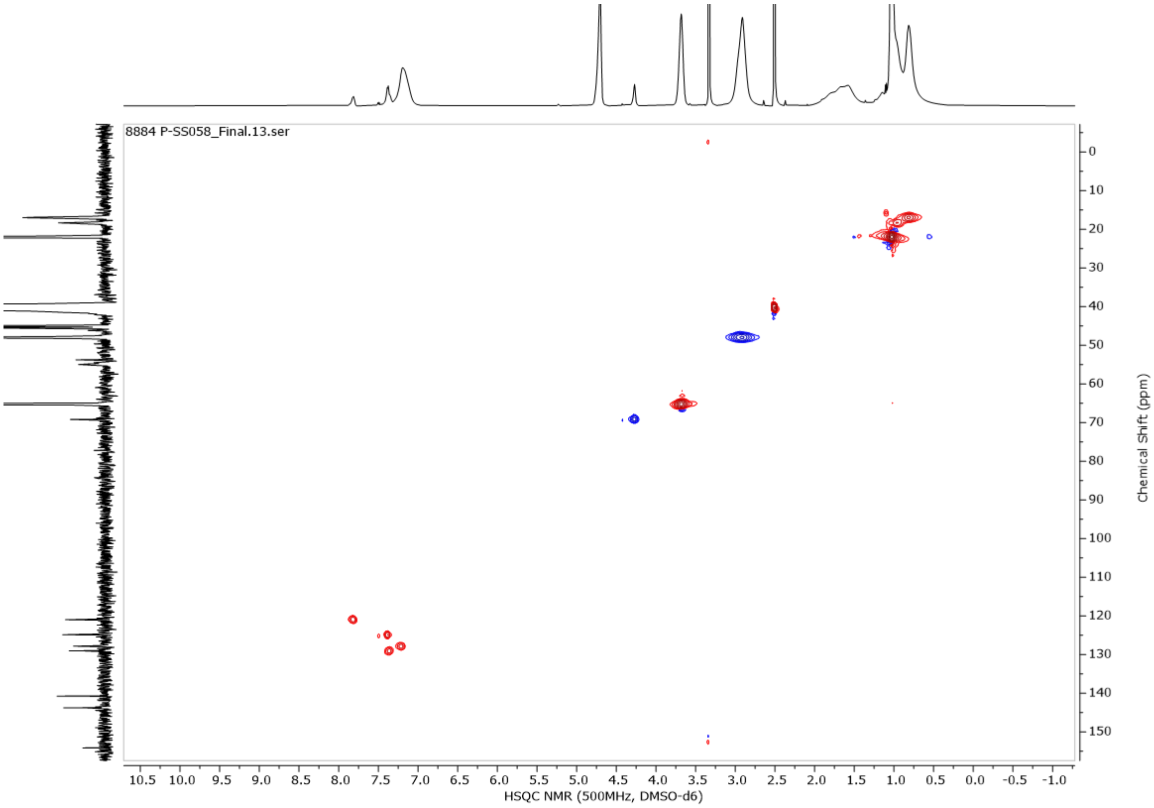
8884 P-SS058_Final.13.ser
HSQC NMR (500MHz, DMSO-d6)
Chemical Shift (ppm)

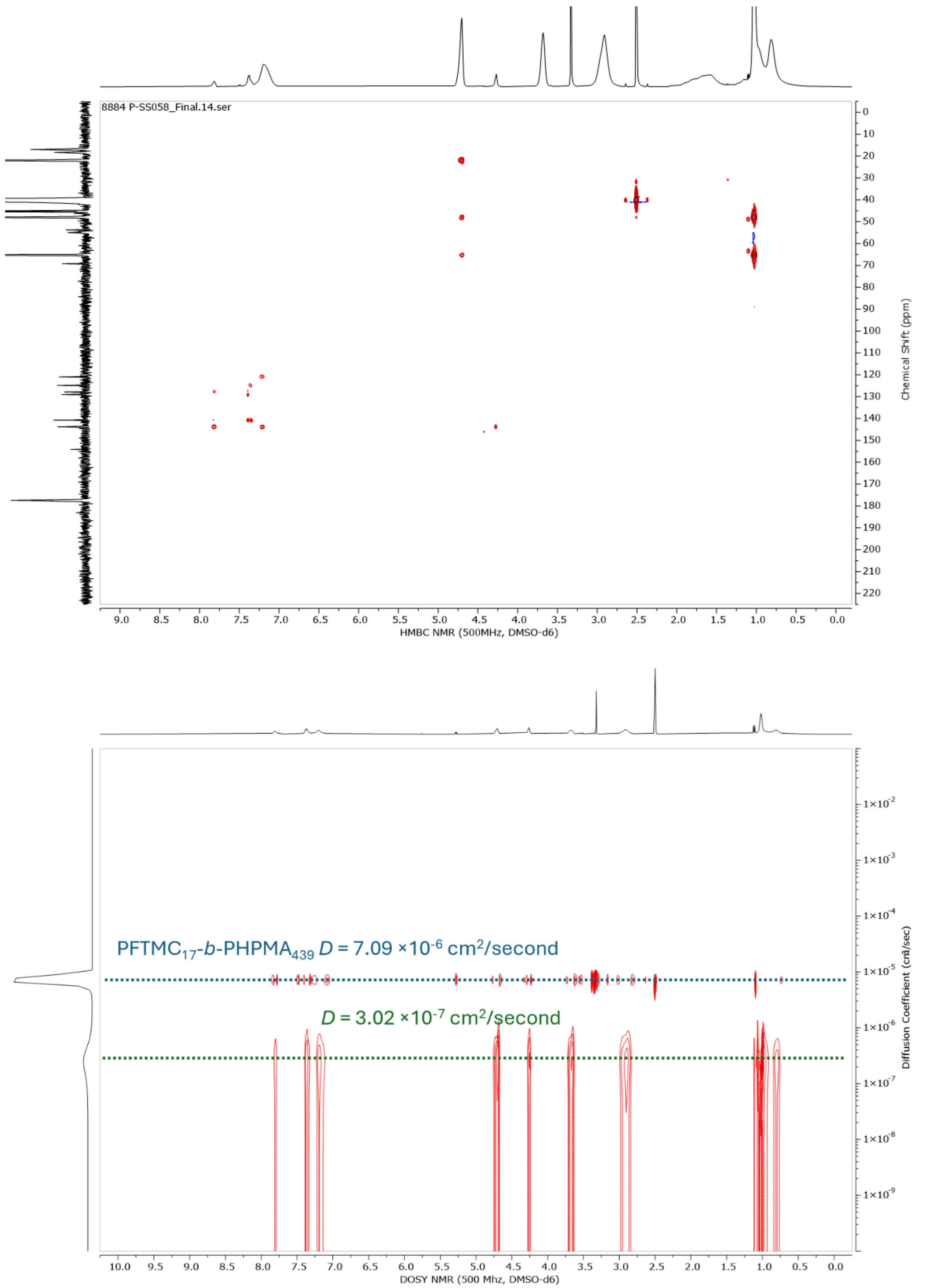

Figure S7. ^{1}H, ^{13}C, COSY, HSQC, HMBC, and DOSY NMR of PFTMC$_{17}$-*b*-PHPMA$_{439}$.

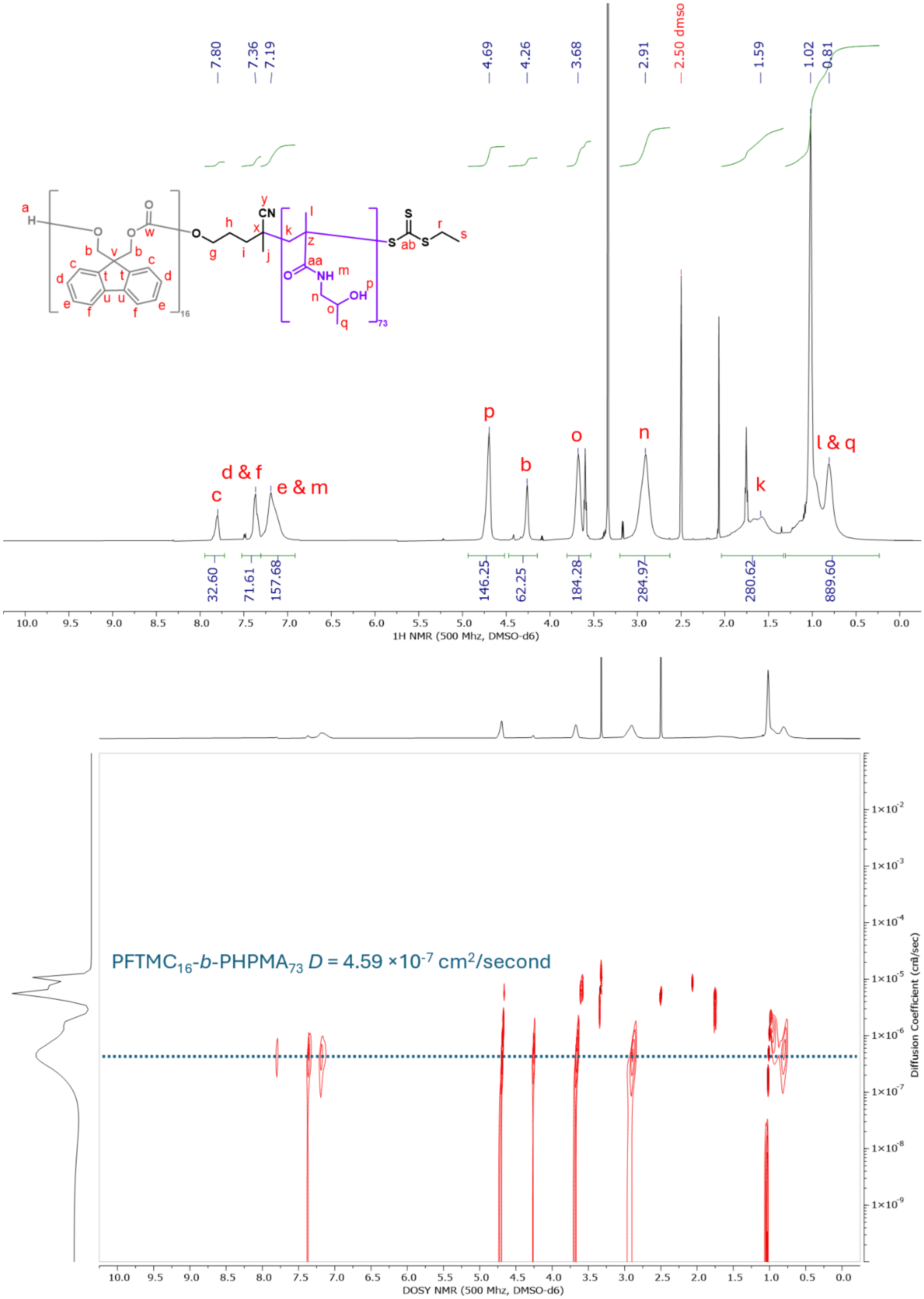

Figure S8. ^{1}H and DOSY NMR of PFTMC$_{16}$-*b*-PHPMA$_{73}$.

Table S2. Molecular weight, composition, and characterization of the polymers studied in this work.

**Polymer**	**M_n (g/mol) via GPC**	**M_w (g/mol) via GPC**	**$Đ_M$ via GPC**	**M_n (g/mol) via NMR**	**PFTMC DP_n via NMR**	**PHPMA DP_n via NMR**	**M_n (g/mol) via MALDI-TOF**	**PFTMC DP_n via MALDI-TOF**	**D (via DOSY NMR, cm^2/sec)**
$PFTMC_{17}$-CTA	4,700	5,700	1.21	5,300	20	-	4,500	17	1.11×10^{-5}
$PFTMC_{17}$-*b*-$PHPMA_{439}$	5,700	10,400	1.83	67,400	17[a]	439	-	-	7.09×10^{-6}
$PFTMC_{16}$-*b*-$PHPMA_{73}$	1,900	5,300	2.81	14,700	16[a]	73	-	-	4.59×10^{-7}
$PFTMC_{16}$-*b*-($PHPMA_{95}$-*co*-$PFlMA_5$)	1,600	10,500	6.58	16,100	16[a]	95	-	-	3.13×10^{-7}

[a] End-groups not visible in DMSO-d_6, so the DP_n of this block is based on the DP_n of $PFTMC_{17}$-CTA.

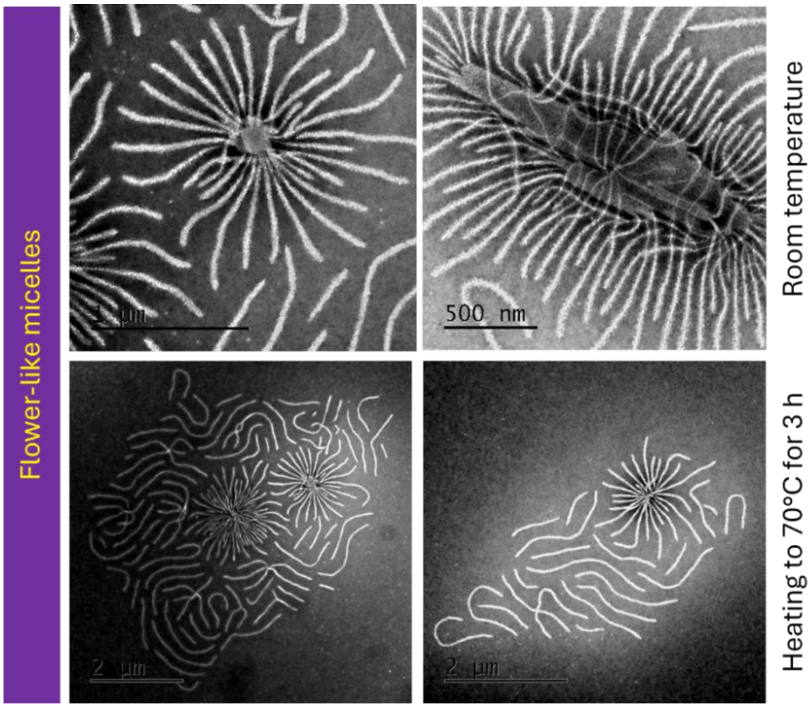

Figure S9. Flower and scarf-like micelles formed from self-nucleation in DMSO/EtOH (5:95 *v*/*v*). All samples were stained with uranyl acetate (3 wt% in EtOH).

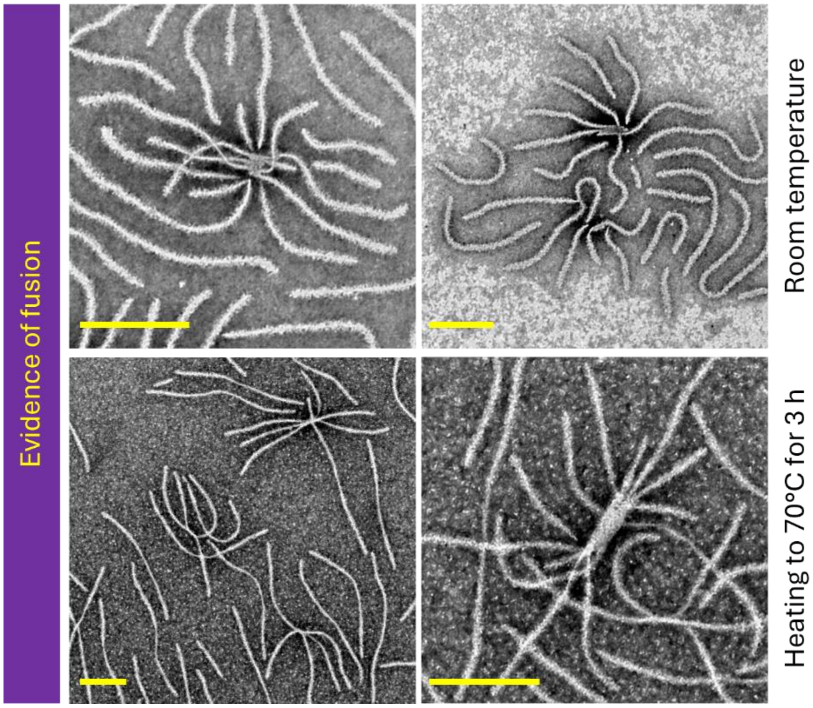

Figure S10. Evidence of nanofiber fusion via side-to-side coupling after self-nucleation in DMSO/EtOH (5:95 *v*/*v*).). Scale = 500 nm. All samples were stained with uranyl acetate (3 wt% in EtOH).

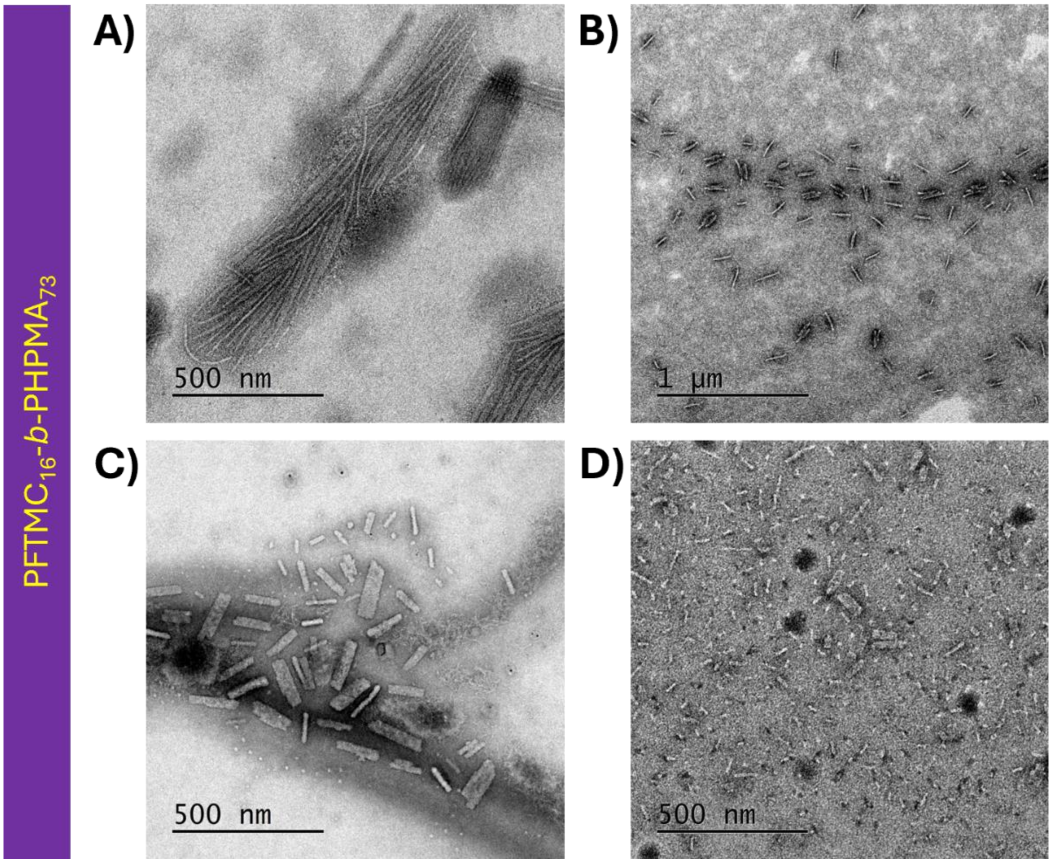

Figure S11. TEM micrographs of PFTMC$_{16}$-*b*-PHPMA$_{73}$ self-assembly in: (A) DMSO/EtOH (5:95 *v*/*v*) after annealing at 70 °C for 1.5 h and ageing at 23 °C for 22.5 h; (B) precision 106 ± 29 nm nanofibers in water after dialysis from DMSO/EtOH (5:95 *v*/*v*); (C) DMSO unimer solution (20 mg/mL) showing the formation of nanoplatelets; (D) neat MeOH after dissolution and ageing at 23 °C for 24 hours (100 mg/mL, 10 wt%). All samples were stained with uranyl acetate (3 wt% in EtOH).

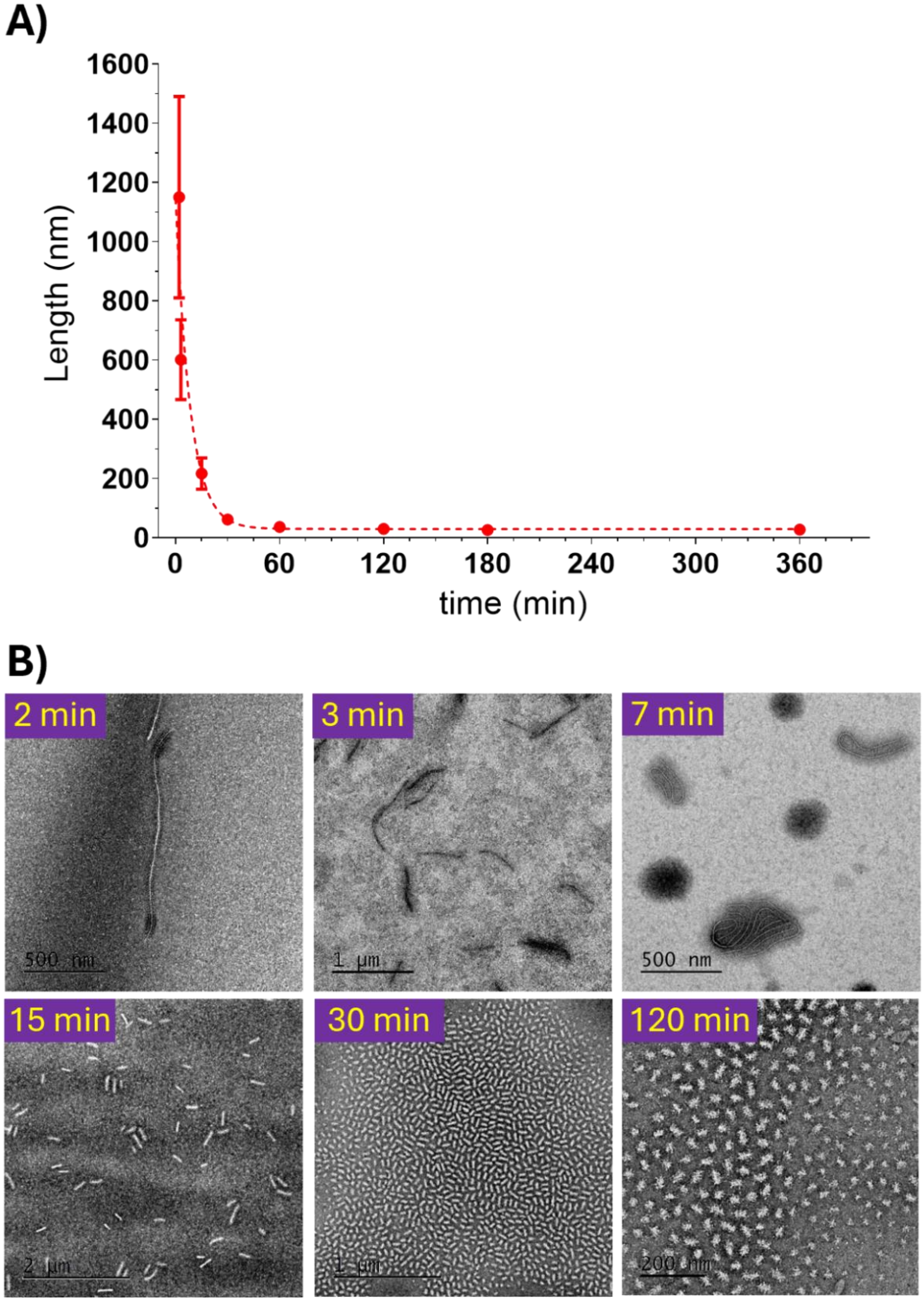

Figure S12. (A) Sonication kinetics of disperse PFTMC$_{17}$-*b*-PHPMA$_{439}$ nanofibers, with fitted curve from non-linear regression. Equation = L_n=(1,144-29.59)*exp(-0.1182*t)+29.59 (where t is time, R^2 = 0.76); (B) TEM micrographs from selected timepoints. All samples were stained with uranyl acetate (3 wt% in EtOH).

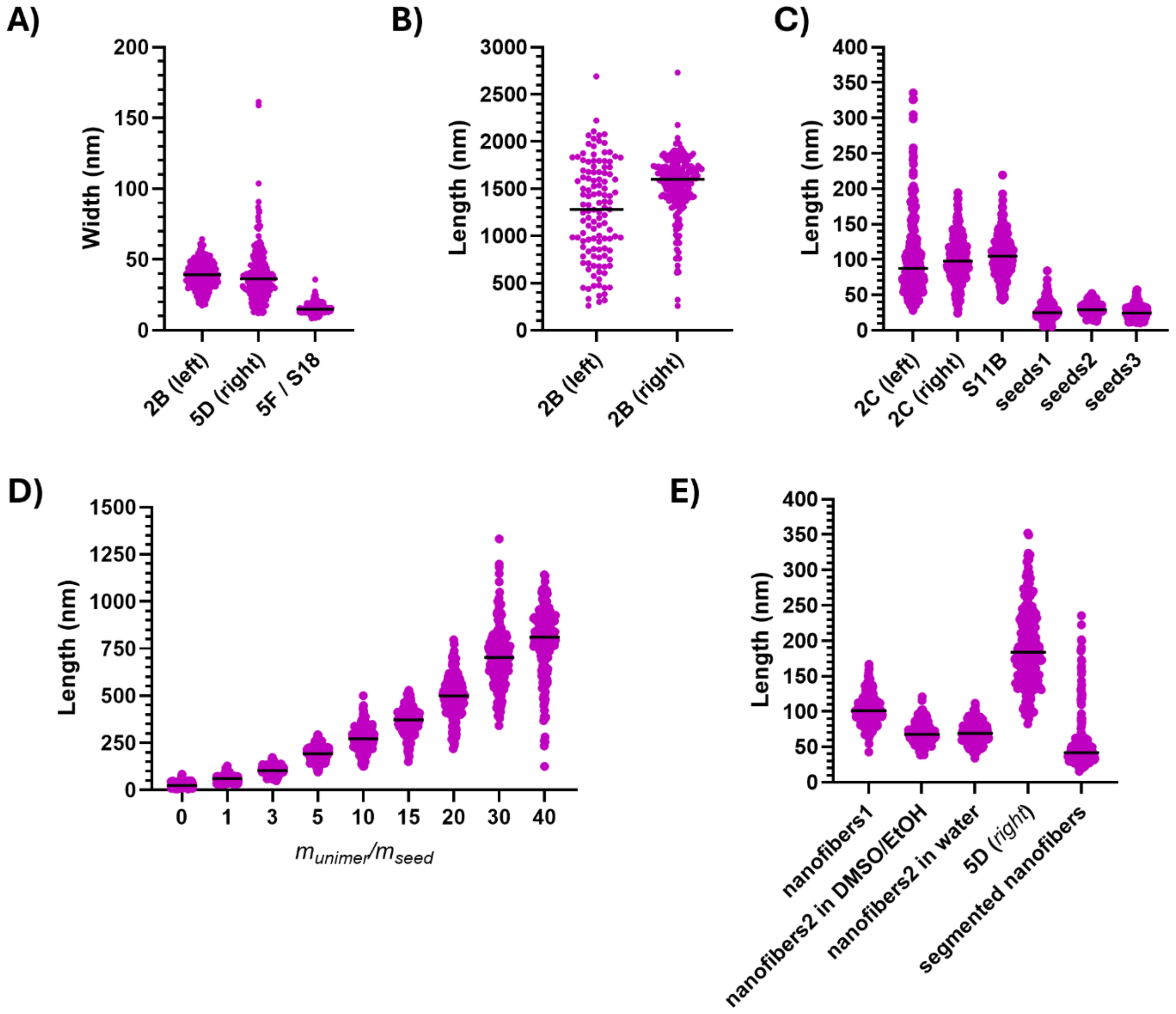

Figure S13. Micelle length and width data for the self-assembled nanoparticles reported in this work. The horizontal line denotes the median length (as opposed to L_n). For samples with no formal name, the figure location for the TEM micrographs are given instead (e.g. 2B (*left*) is the left TEM micrograph from subfigure 2B). (A) micelle width analysis; (B) micelle length analysis for spontaneous nucleation of PFTMC$_{17}$-*b*-PHPMA$_{439}$; (C) micelle length analysis for PFTMC$_{16}$-*b*-PHPMA$_{73}$ and all seed nanofibers; (D) micelle length analysis from the living CDSA of PFTMC$_{17}$-*b*-PHPMA$_{439}$ from nanofiber **seeds1**; (E) micelle length analysis from the precision nanofibers used in biological and AFM analysis.

Table S3. Statistical analysis of the contour length and length-dispersity of the nanofibers reported in this work.

TEM Micrograph[a]	Name/Condition	m_{unimer} / m_{seed}	*n*	L_n (nm)	L_w (nm)	$Đ_L$	σ_L (nm)	σ_L / L_n
2C (*left*)	Crude reaction mixture (MeOH)[b]	-	275	102.08	134.39	1.32	57.5	0.56
2B (*left*)	DMSO/EtOH (rt)[c]	-	220	1149.19	1397.69	1.22	534.4	0.47
2B (*right*)	DMSO/EtOH (70°C)[c]	-	220	1540.55	1605.41	1.04	316.1	0.21
2C (*right*)	DMSO/EtOH (rt)[b]	-	225	99.18	108.70	1.10	30.7	0.31
S11B	Fig. 2C (*right*)[b] after transfer into water[b]	-	249	106.10	114.00	1.07	28.9	0.27
3A	**seeds1**[c]	-	221	27.19	32.41	1.19	11.9	0.44
3B	**seeds2**[c]	-	242	30.16	32.25	1.07	7.9	0.26
3C	**seeds3**[b]	-	226	25.46	28.32	1.11	8.5	0.34
3F	u/s=1[c]	1	254	61.19	65.09	1.06	15.4	0.25
3F	u/s=3[c]	3	223	102.33	106.83	1.04	21.5	0.21
3F	u/s=5[c]	5	248	192.58	199.65	1.04	36.9	0.19
3F	u/s=10[c]	10	204	275.90	289.78	1.05	61.9	0.22
S14A	u/s=15[c]	15	265	367.47	379.48	1.03	66.4	0.18
3F	u/s=20[c]	20	227	494.57	516.91	1.05	105.2	0.21
3F	u/s=30[c]	30	203	706.77	740.75	1.05	155.0	0.22
S14B	u/s=40[c]	40	216	786.97	825.69	1.05	174.6	0.22

S15B	**nanofibers1**[c]	2	238	102.76	106.74	1.04	20.2	0.20
S16A	**nanofibers2** in DMSO/EtOH[c]	2	260	69.87	72.86	1.04	14.4	0.21
3D / S16B	**nanofibers2** in water[c]	2	248	69.91	72.87	1.04	14.4	0.21
5D (*right*)	DMSO/EtOH (70°C)[d]	-	241	189.51	204.61	1.08	53.5	0.28
5E)	**segmented nanofibers 1**[c, d]	1	248	54.60	82.86	1.52	39.3	0.72

[a] This is the location of the figure with the TEM micrograph.
[b] This sample contains PFTMC$_{16}$-*b*-PHPMA$_{73}$.
[c] This sample contains PFTMC$_{17}$-*b*-PHPMA$_{439}$.
[d] This sample contains PFTMC$_{16}$-*b*-(PHPMA$_{95}$-*co*-PFlMA$_{5}$)

Table S4. Statistical analysis of the contour width and width-dispersity of the nanofibers reported in this work.

TEM Micrograph[a]	Name/Condition	m_{unimer} / m_{seed}	n	W_n (nm)	W_w (nm)	$Đ_W$	σ_W (nm)	σ_W / L_n
2B (*left*)	DMSO/EtOH (rt)[a]	-	220	39.07	41.50	1.06	9.75	0.25
5D (*right*)	DMSO/EtOH (70°C)[a, b]	-	214	39.27	49.44	1.26	20.0	0.51
5F / S19	**blended nanospheres 1**[a, b, c]	-	217	15.68	16.62	1.06	3.84	0.24

[a] This sample contains PFTMC$_{17}$-*b*-PHPMA$_{439}$.
[b] This sample contains PFTMC$_{16}$-*b*-(PHPMA$_{95}$-*co*-PFlMA$_{5}$)
[c] This sample is spherical, so W_n, W_w, $Đ_W$, and σ_W correspond to the diameter (D_n, D_w, $Đ_D$, and σ_D), not width.

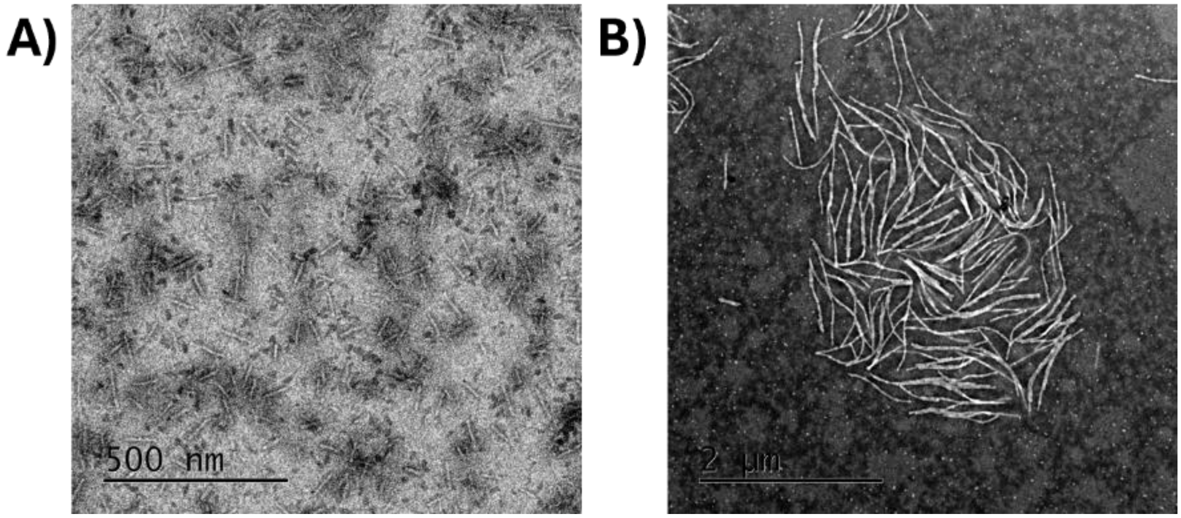

Figure S14. PFTMC$_{17}$-*b*-PHPMA$_{439}$ nanofibers from living CDSA studies: (A) u/s = 15 and (B) u/s = 40. All samples were stained with uranyl acetate (3 wt% in EtOH).

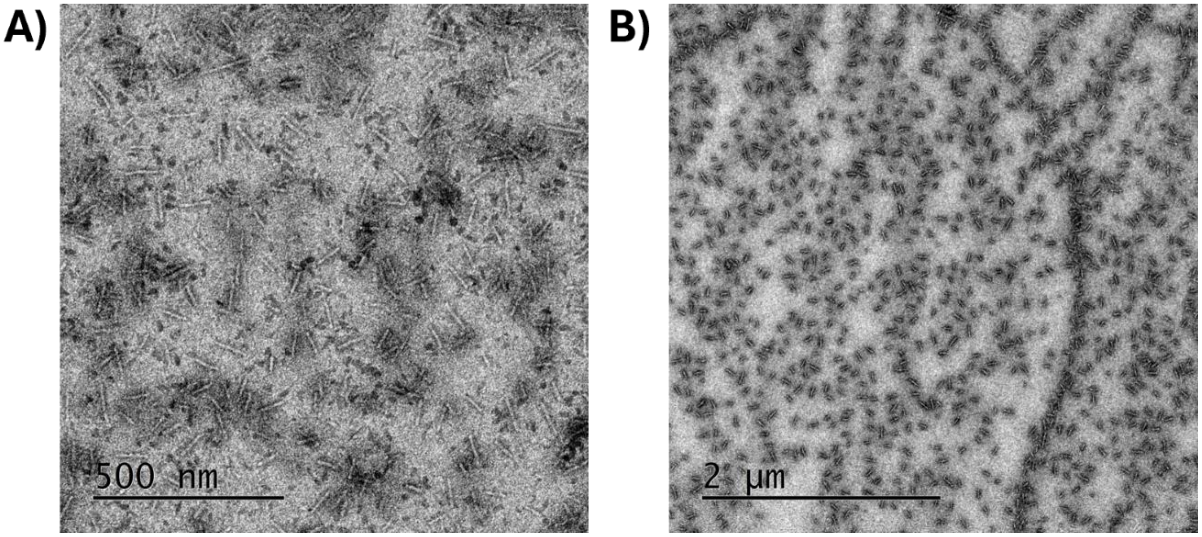

Figure S15. TEM micrographs of 70 ± 14 nm **nanofibers2** used for cytotoxicity studies in (A) DMSO/EtOH (5:95 *v*/*v*) after formation, and (B) in water at low magnification. All samples were stained with uranyl acetate (3 wt% in EtOH).

Table S5. Change in reductive metabolism of WI-38 and HeLa cells upon addition of 70 ± 14 nm **nanofibers2** as measured by a 72 h alamarBlue™ assay. Values are expressed as % of control.

[**nanofibers2**] (µg/mL)		Control	100	75	50	25	10	5	2.5	1	Staurosporine (1 µM /mL)
WI-38	Mean	100	119	116	113	105	107	110	96.7	111	-3.12
	σ	4.01	8.97	7.72	4.94	10.1	5.87	12.0	10.9	6.53	6.06
HeLa	Mean	100	101	105	100	101	103	101	93.0	107	-1.65
	σ	8.77	11.2	11.8	10.6	12.2	11.4	11.7	12.6	15.6	3.66

Table S6. Change in cell viability of WI-38 and HeLa cells upon addition of 70 ± 14 nm **nanofibers2** as measured by a 72 h calcein AM assay. assay. Values are expressed as % of control.

[**nanofibers2**] (µg/mL)		Control	100	75	50	25	10	5	2.5	1	Staurosporine (1 µM /mL)
WI-38	Mean	100	80.7	87.8	80.5	81.8	80.9	87.8	91.2	94.3	0.45
	σ	6.2	10.0	8.57	7.15	7.87	10.3	11.9	8.3	8.18	0.63
HeLa	Mean	100	94.9	99.7	95.7	101	99.3	97.1	93.0	100	-0.44
	σ	9.00	11.2	11.1	15.8	11.8	11.5	14.7	12.6	17.0	0.25

Table S7. Tabulated statistical significance of the cytotoxicity experiments conducted with 70 ± 14 nm **nanofibers2** with HeLa cells after 72 h incubation (Figure 4, *top*).

**Dunnett's multiple comparisons test**	**Predicted (LS) mean diff.**	**95.00% CI of diff.**	**Below threshold?**	**Summary**	**Adjusted P Value**
**Calcein**					
control vs. blank	100.0	90.75 to 109.2	Yes	****	<0.0001
control vs. 100	5.065	-4.284 to 14.41	No	ns	0.6239
control vs. 75	0.3201	-8.929 to 9.569	No	ns	>0.9999
control vs. 50	4.311	-4.938 to 13.56	No	ns	0.7782
control vs. 25	-1.065	-10.31 to 8.185	No	ns	>0.9999
control vs. 10	0.6830	-8.566 to 9.932	No	ns	>0.9999
control vs. 5	2.947	-6.302 to 12.20	No	ns	0.9696
control vs. 2	7.005	-2.244 to 16.25	No	ns	0.2388
control vs. 1	-0.08589	-9.335 to 9.163	No	ns	>0.9999
control vs. Staurosporine	100.4	89.11 to 111.8	Yes	****	<0.0001

**Alamar Blue**					
control vs. blank	100.0	90.75 to 109.2	Yes	****	<0.0001
control vs. 100	-1.621	-10.87 to 7.627	No	ns	0.9997
control vs. 75	-4.909	-14.16 to 4.338	No	ns	0.6464
control vs. 50	-0.4918	-9.740 to 8.756	No	ns	>0.9999
control vs. 25	-1.708	-10.96 to 7.540	No	ns	0.9995
control vs. 10	-3.664	-12.91 to 5.584	No	ns	0.8936
control vs. 5	-1.257	-10.50 to 7.991	No	ns	>0.9999
control vs. 2	-2.014	-11.26 to 7.234	No	ns	0.9981
control vs. 1	-6.810	-16.06 to 2.438	No	ns	0.2670
control vs. Staurosporine	101.7	90.33 to 113.0	Yes	****	<0.0001

Table S8. Tabulated statistical significance of the cytotoxicity experiments conducted with 70 ± 14 nm **nanofibers2** with WI-38 cells after 72 h incubation (Figure 4, *bottom*).

Dunnett's multiple comparisons test	Predicted (LS) mean diff.	95.00% CI of diff.	Below threshold?	Summary	Adjusted P Value
**Calcein**					
control vs. blank	100.0	90.74 to 109.3	Yes	****	<0.0001
control vs. 100	19.34	10.08 to 28.60	Yes	****	<0.0001
control vs. 75	12.22	2.954 to 21.48	Yes	**	0.0035
control vs. 50	19.52	10.26 to 28.78	Yes	****	<0.0001
control vs. 25	18.20	8.934 to 27.46	Yes	****	<0.0001
control vs. 10	19.08	9.625 to 28.53	Yes	****	<0.0001
control vs. 5	12.22	2.956 to 21.48	Yes	**	0.0035
control vs. 2	8.807	-0.4559 to 18.07	No	ns	0.0705
control vs. 1	5.691	-3.572 to 14.95	No	ns	0.4493
control vs. Staurosporine	99.56	90.29 to 108.8	Yes	****	<0.0001

**Alamar Blue**					
control vs. blank	100.0	91.09 to 108.9	Yes	****	<0.0001
control vs. 100	-19.42	-28.76 to -10.08	Yes	****	<0.0001
control vs. 75	-16.31	-25.41 to -7.202	Yes	****	<0.0001
control vs. 50	-13.34	-22.44 to -4.232	Yes	***	0.0007
control vs. 25	-4.939	-13.85 to 3.967	No	ns	0.5901
control vs. 10	-7.215	-16.32 to 1.891	No	ns	0.1941
control vs. 5	-9.751	-18.66 to -0.8444	Yes	*	0.0241
control vs. 2	3.268	-5.638 to 12.17	No	ns	0.9259
control vs. 1	-10.53	-19.43 to -1.619	Yes	*	0.0116
control vs. Staurosporine	103.1	94.22 to 112.0	Yes	****	<0.0001

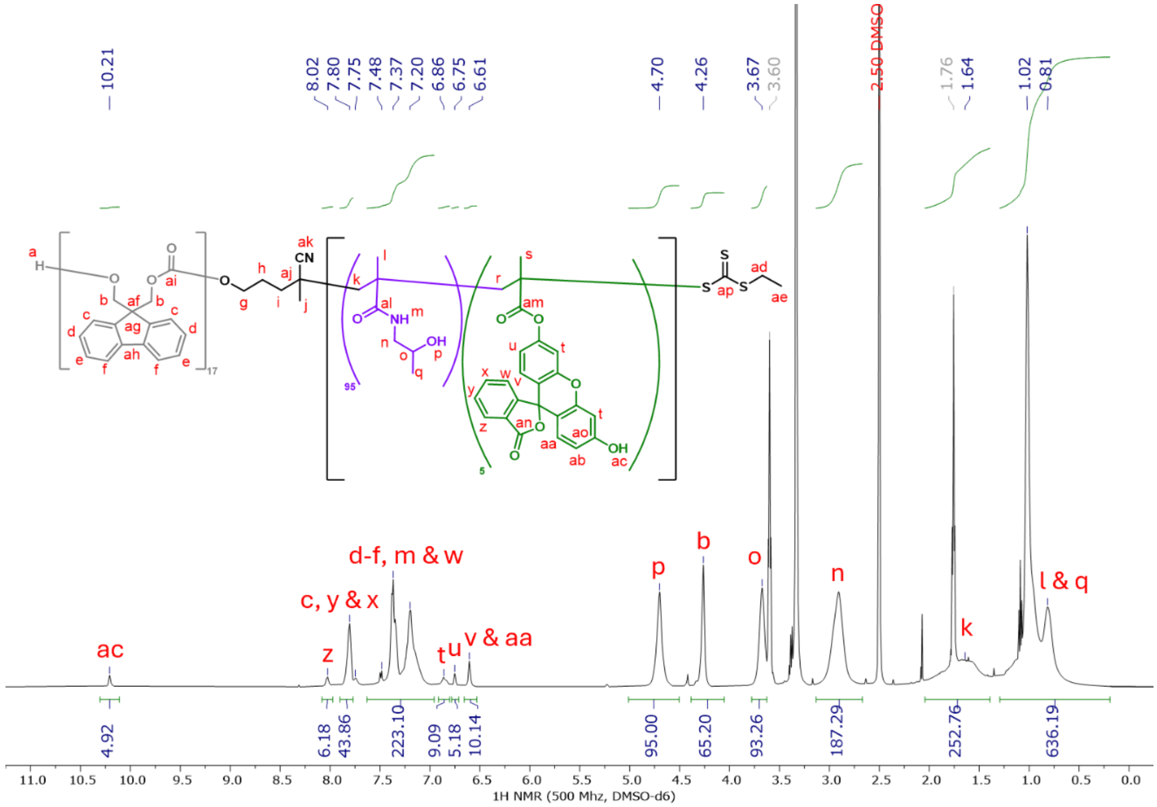
1H NMR (500 Mhz, DMSO-d6)

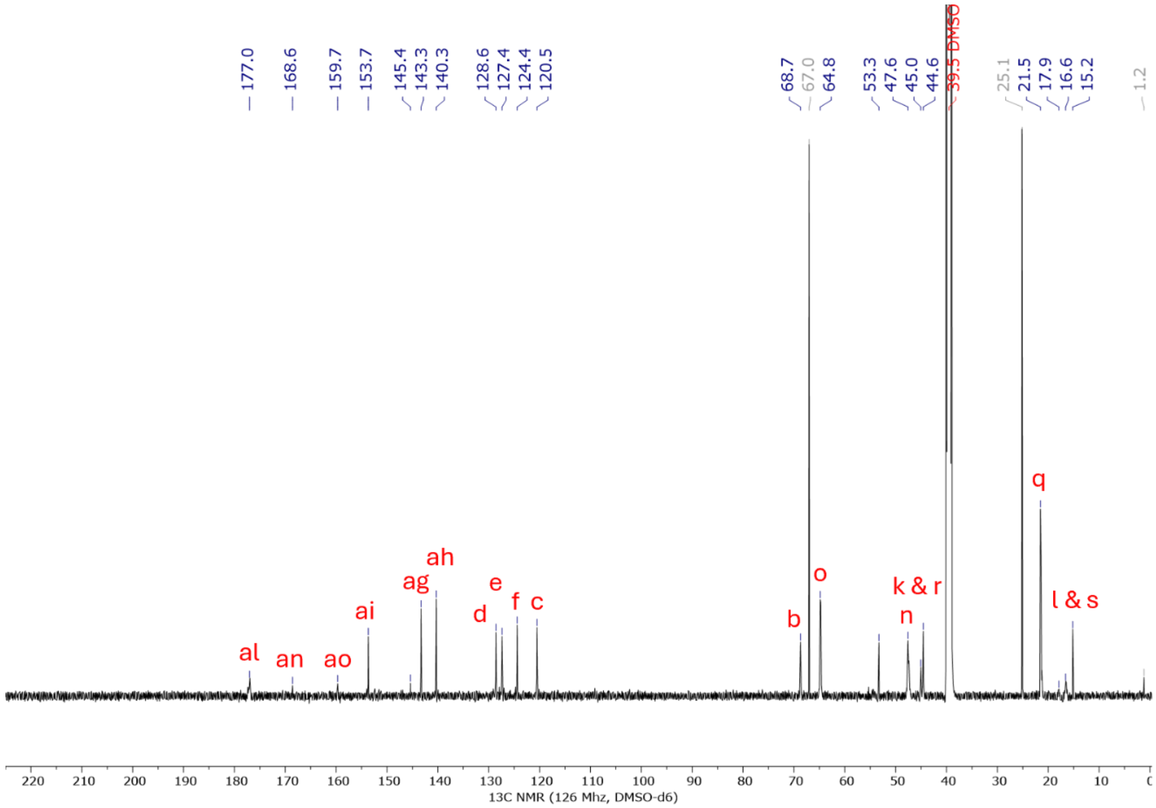
13C NMR (126 Mhz, DMSO-d6)

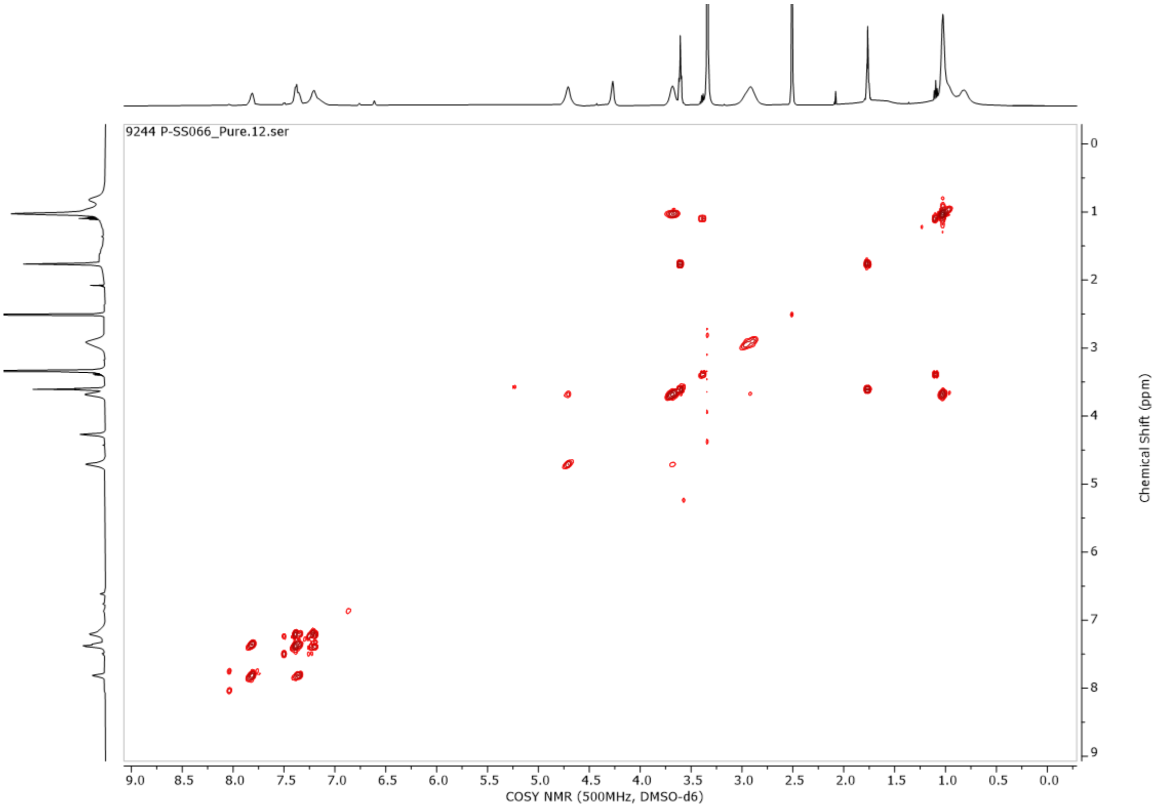
9244 P-SS066_Pure.12.ser
Chemical Shift (ppm)
COSY NMR (500MHz, DMSO-d6)

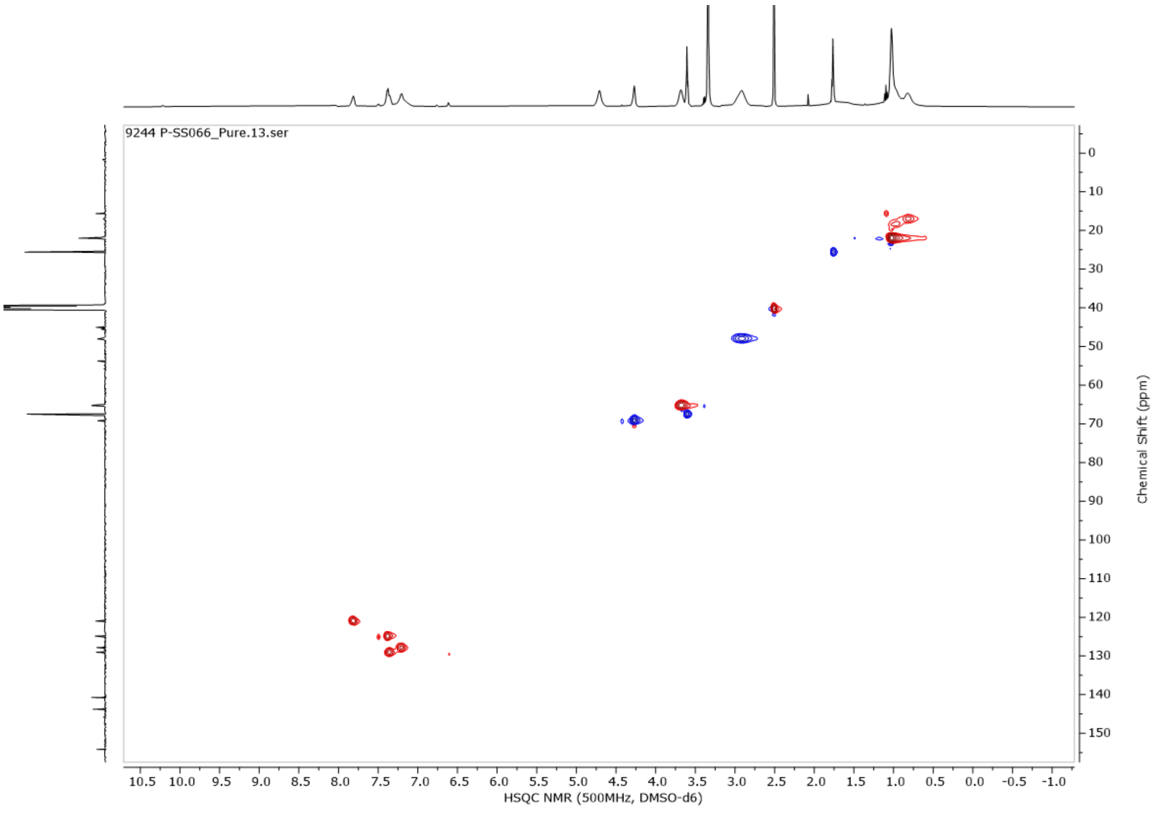
9244 P-SS066_Pure.13.ser
Chemical Shift (ppm)
HSQC NMR (500MHz, DMSO-d6)

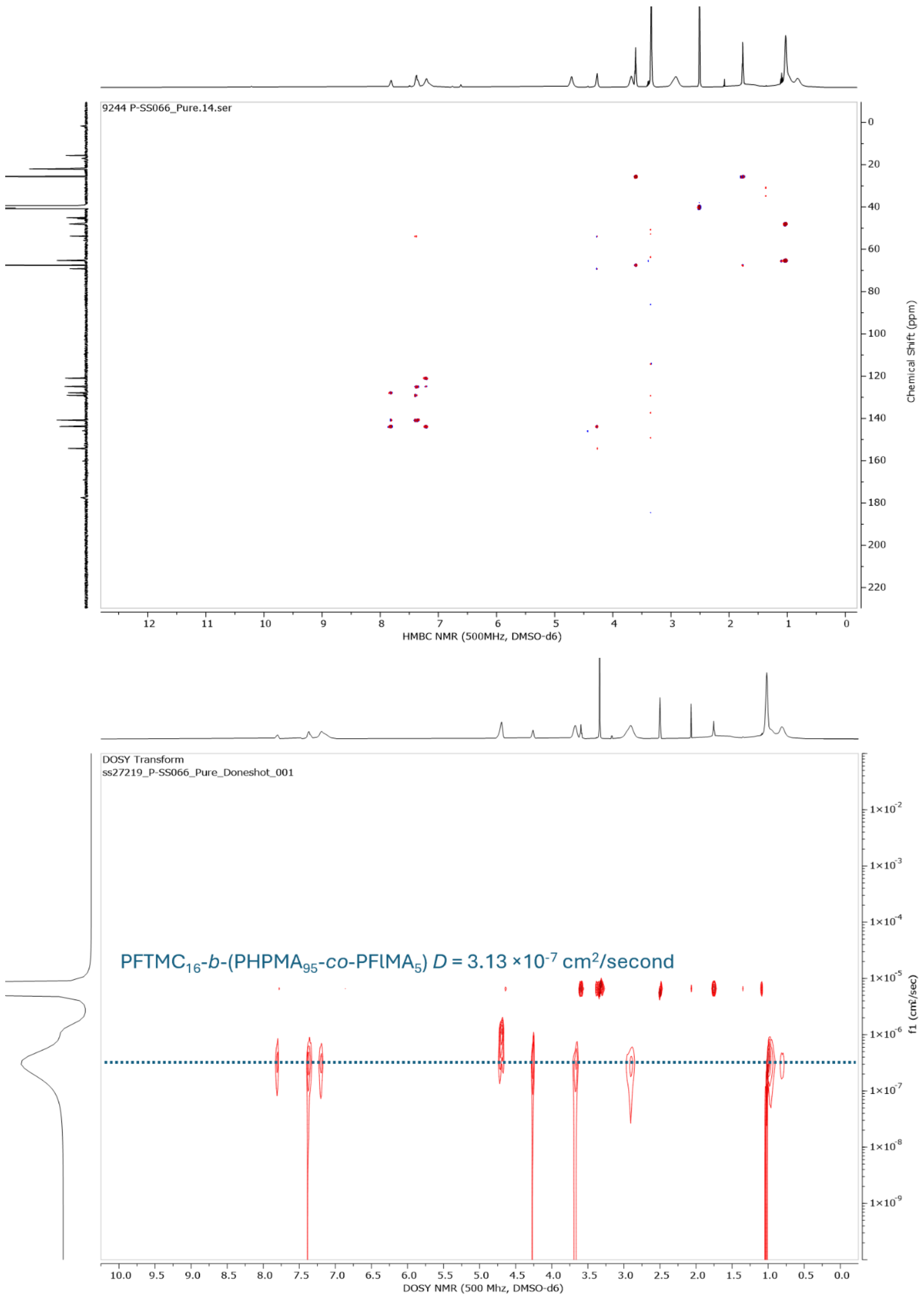

Figure S16. ^{1}H, ^{13}C, COSY, HSQC, HMBC, and DOSY NMR of PFTMC-(PHPMA$_{95}$-*co*-PFlMA$_{5}$).

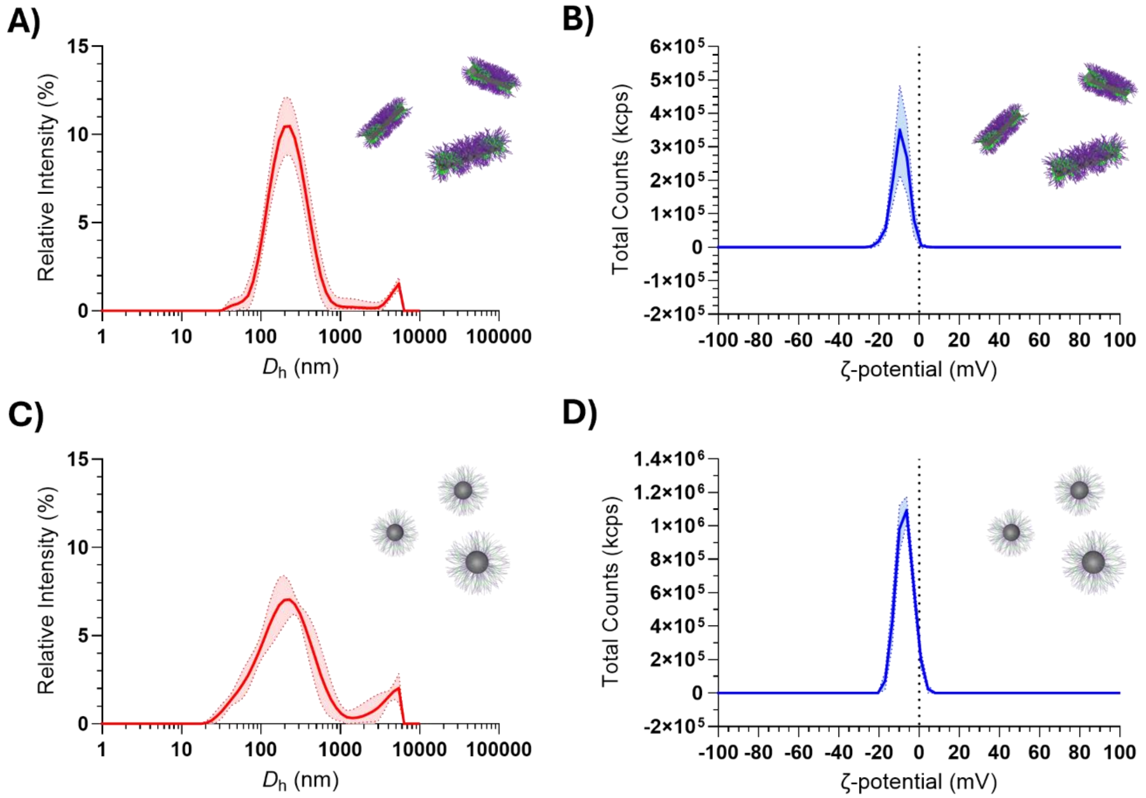

Figure S17. (A, C) DLS and (B, D) ζ-potential data for (A-B) 55 ± 40 nm [PFTMC$_{16}$-*b*-(PHPMA$_{95}$-*co*-PFlMA$_5$)]-*m*-[PFTMC$_{17}$-*b*-PHPMA$_{439}$]-*m*-[PFTMC$_{16}$-*b*-(PHPMA$_{95}$-*co*-PFlMA$_5$)] triblock **segmented nanofibers 1** and (C-D) 16 ± 4 nm **blended nanospheres 1**.

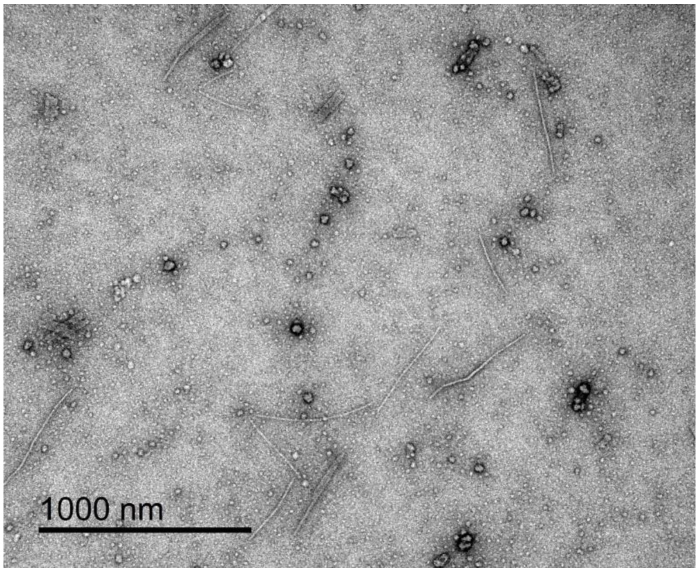

Figure S18. 16 ± 4 nm **blended nanospheres 1** were also observed to contain a small number of nanofibers. The sample was stained with uranyl acetate (3 wt% in EtOH).

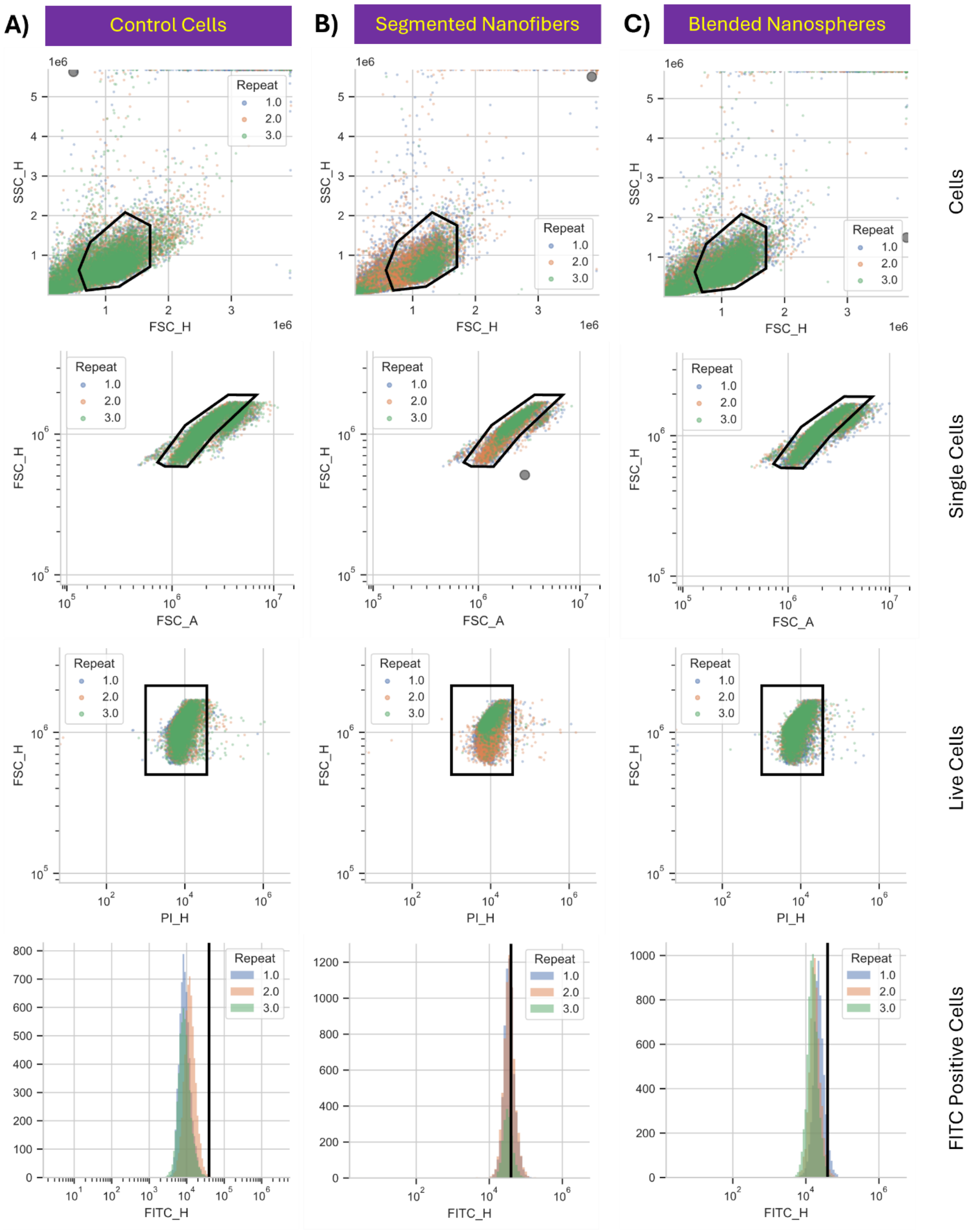

Figure S19. Flow cytometry gating history for U-87 MG cells incubated with (A) no nanoparticles (control cells), (B) FITC labelled 55 ± 40 nm **segmented nanofibers 1** and (C) 16 ± 4 nm **blended nanospheres 1** for 1 h. $n = 3$.

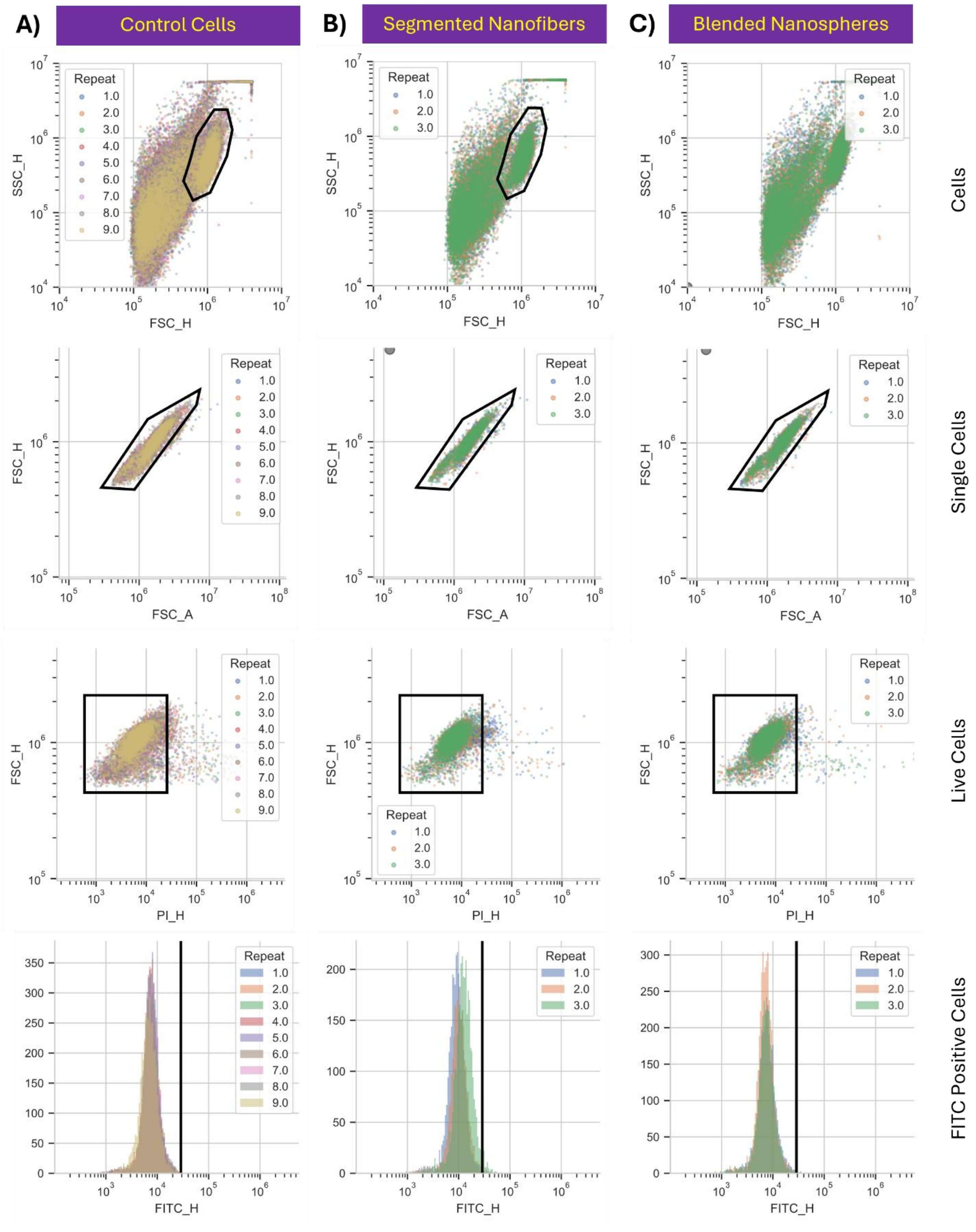

Figure S20. Flow cytometry gating history for U-87 MG cells incubated with (A) no nanoparticles (control cells), (B) FITC labelled 55 ± 40 nm **segmented nanofibers 1** and (C) 16 ± 4 nm **blended nanospheres 1** for 24 h. n = 3.

Table S9. Tabulated flow cytometry statistics for the cellular uptake of FlMA labelled 55 ± 40 nm **segmented nanofibers 1** and 16 ± 4 nm **blended nanospheres 1** after incubation with U-87 MG cells for 1 h (Figure 6C-D). Where units are not specified, the values represent the number of events.

Sample	Control	Segmented nanofibers 1	Blended nanospheres 1
**Cells counted**	30,000	22,429	30,000
**'Cells'**	19,332	15,700	20,551
**'Single cells'**	17,514	15,220	19,370
**'Live cells' (PI negative)**	17,369	15,073	19,271
**FlMA positive cells**	6	5,121	525
**Median fluorescence intensity of FlMA (a.u.)**	9,654.6	34,716.9	18,047.4
***σ* of FlMA fluorescence (a.u.)**	4,481.05	15,756.7	8,216
**Median Side Scatter Height (SSC-H) of live cells**	670,068	649,087	654,611
**σ of (SSC-H) of live cells**	265,711	256,412	265,350

Table S10. Tabulated flow cytometry statistics for the cellular uptake of FlMA labelled 55 ± 40 nm **segmented nanofibers 1** and 16 ± 4 nm **blended nanospheres 1** after incubation with U-87 MG cells for 24 h (Figure 6C-D). Where units are not specified, the values represent the number of events.

Sample	Control	Segmented nanofibers 1	Blended nanospheres 1
**Cells counted**	90,000	30,000	30,000
**'Cells'**	34,722	9,478	11,240
**'Single cells'**	34,577	9,443	11,217
**'Live cells' (PI negative)**	34,071	9,134	11,034
**FlMA positive cells**	21	107	12
**Median fluorescence intensity of FlMA (a.u.)**	7,391.2	10,469.1	7,347.9
**σ of FlMA fluorescence (a.u.)**	3,474.09	5,424.1	3,631.6
**Median Side Scatter Height (SSC-H) of live cells**	500,265	482,914	474,488
**σ of (SSC-H) of live cells**	234,163	219,645	218,847

Table S11. Tabulated statistical significance of the % FlMA positive cells from flow cytometry experiments of 55 ± 40 nm **segmented nanofibers 1** and 16 ± 4 nm **blended nanospheres 1** with U-87 MG cells after incubation for either 1 h or 24 h (Figure 6C).

Tukey's multiple comparisons test	Predicted (LS) mean diff.	95.00% CI of diff.	Below threshold?	Summary	Adjusted P Value
1 h					
Control vs. Nanofibers	-33.94	-37.96 to -29.92	Yes	****	<0.0001
Control vs. Nanospheres	-2.690	-6.705 to 1.326	No	ns	0.2404
Nanofibers vs. Nanospheres	31.25	27.23 to 35.27	Yes	****	<0.0001
24 h					
Control vs. Nanofibers	-1.110	-3.428 to 1.209	No	ns	0.4740
Control vs. Nanospheres	-0.04712	-2.366 to 2.271	No	ns	0.9986
Nanofibers vs. Nanospheres	1.063	-1.256 to 3.381	No	ns	0.5035

Šídák's multiple comparisons test	Predicted (LS) mean diff.	95.00% CI of diff.	Below threshold?	Summary	Adjusted P Value
1 h - 24 h					
Control	-0.02709	-3.390 to 3.336	No	ns	>0.9999
Nanofibers	32.80	29.44 to 36.17	Yes	****	<0.0001
Nanospheres	2.616	-0.7473 to 5.978	No	ns	0.1655

Table S12. Tabulated statistical significance of the median FlMA intensity from flow cytometry experiments of 55 ± 40 nm **segmented nanofibers 1** and 16 ± 4 nm **blended nanospheres 1** with U-87 MG cells after incubation for either 1 h or 24 h (Figure 6D).

**Tukey's multiple comparisons test**	**Predicted (LS) mean diff.**	**95.00% CI of diff.**	**Below threshold?**	**Summary**	**Adjusted P Value**
1 h					
Control vs. Nanofibers	-25062	-25262 to -24862	Yes	****	<0.0001
Control vs. Nanospheres	-8393	-8581 to -8205	Yes	****	<0.0001
Nanofibers vs. Nanospheres	16670	16474 to 16865	Yes	****	<0.0001
24 h					
Control vs. Nanofibers	-3078	-3289 to -2866	Yes	****	<0.0001
Control vs. Nanospheres	43.30	-153.4 to 240.0	No	ns	0.8635
Nanofibers vs. Nanospheres	3121	2867 to 3375	Yes	****	<0.0001

Šídák's multiple comparisons test	Predicted (LS) mean diff.	95.00% CI of diff.	Below threshold?	Summary	Adjusted P Value
1 h - 24 h					
Control	2263	2093 to 2434	Yes	****	<0.0001
Nanofibers	24248	24005 to 24490	Yes	****	<0.0001
Nanospheres	10700	10481 to 10918	Yes	****	<0.0001

References

[1] B. Teague, *bioRxiv* **2022**, DOI 10.1101/2022.07.22.501078.

[2] S. T. G. Street, Y. He, R. L. Harniman, J. D. Garcia-Hernandez, I. Manners, *Polym. Chem.* **2022**, *13*, 3009–3025.

[3] M. C. Arno, M. Inam, Z. Coe, G. Cambridge, L. J. Macdougall, R. Keogh, A. P. Dove, R. K. O'Reilly, *J. Am. Chem. Soc.* **2017**, *139*, 16980–16985.

[4] N. Corrigan, A. Almasri, W. Taillades, J. Xu, C. Boyer, *Macromolecules* **2017**, *50*, 8438–8448.

[5] G. Guérin, H. Wang, I. Manners, M. A. Winnik, *J. Am. Chem. Soc.* **2008**, *130*, 14763–14771.
